# A developmental shift in glucocorticoid receptor expression preserves glucocorticoid sensitivity in the adult suprachiasmatic nucleus

**DOI:** 10.1371/journal.pbio.3003870

**Published:** 2026-07-07

**Authors:** Kristian Händler, Varun K. A. Sreenivasan, Violetta Pilorz, Celia Martinez-Perez, Iratxe Elorduy, Tomas J. Casas, Marianne Lehmann, Jon Olano Bringas, Laura Escobar Castañondo, Nora Bengoa-Vergniory, Federico N. Soria, Henrik Oster, Malte Spielmann, Mariana Astiz

**Affiliations:** 1 Institute for Medical and Human Genetics, Charité-Universitätsmedizin Berlin, Berlin, Germany; 2 Human Molecular Genetics Group, Max Planck Institute for Molecular Genetics, Berlin, Germany; 3 German Center for Child and Adolescent Health (DZKJ), Partner Site Berlin, Berlin, Germany; 4 Institute of Neurobiology, Center of Brain, Behavior and Metabolism (CBBM), University of Lübeck, Lübeck, Germany; 5 Laboratory of Circadian Physiology, Achucarro Basque Center for Neuroscience, Leioa, Spain; 6 Laboratory of Aggregation and Glial Response, Achucarro Basque Center for Neuroscience, Leioa, Spain; 7 Imaging Facility, Achucarro Basque Center for Neuroscience, Leioa, Spain; 8 IKERBASQUE, Basque Foundation for Science, Bilbao, Spain; 9 Laboratory of Glia and Matrix Biology, Achucarro Basque Center for Neuroscience, Leioa, Spain; INSERM, FRANCE

## Abstract

The circadian system synchronizes physiology, improving the adaptation to daily environmental changes. In mammals, the central pacemaker, in the suprachiasmatic nuclei (SCN) of the hypothalamus, coordinates “wake” functions by inducing the circadian release of glucocorticoids (GCs). GCs entrain the clocks of a wide variety of tissues through GC receptor (GR) activation, however, the influence of GCs on the SCN is unclear and seems to depend on the maturity of the circuit. During the perinatal period, the mouse SCN express GR and respond directly to GCs while the adult SCN express low GR and have been traditionally considered resistant to GCs. To understand the change of sensitivity to GCs we followed the developmental trajectory of the mouse SCN, and found that while GR is expressed in all SCN cells early in life, it remains expressed mainly in astrocytes in the adult. Using a model of prenatal exposure to GCs, we found that offspring from treated mothers, adapt slower to shifted light–dark cycle and shows reduced expression of GR in SCN astrocytes. The adult SCN astrocytes can indeed sense and respond to GCs with rapid astrocytic Ca^2+^ events that propagate across neighboring cells, an effect that is prevented by the specific inhibition of astrocyte–astrocyte communication. Our findings provide a conceptual advance on how the mouse clock develops and on the influence that GCs have on the SCN. This might be relevant to understand how circadian synchrony is restored in conditions of temporal misalignment, such as jet lag.

## Abstract 2

The suprachiasmatic nucleus (SCN) is the central circadian pacemaker, and is responsive to glucocorticoid signaling early in development but thought to become resistant to glucocorticoids in adulthood. This study shows the adult SCN retains glucocorticoid sensitivity, but that receptor expression shifts during development and becomes astrocyte-specific in adulthood.

## Introduction

Most organisms on Earth harbor a 24-hour circadian timing system, which anticipates daily recurring changes in the environment and ensure physiological fitness [1]. In mammals, the organization of the circadian system is hierarchical, with a central pacemaker located in the hypothalamic suprachiasmatic nuclei (SCN) [2–4]. The SCN receive environmental light input from the retina and synchronize hormonal and neuronal output pathways to convey time to every single cell of the body [5–7]. The circadian regulation of glucocorticoid (GC) release, one of the main hormonal outputs, requires a complex cooperation between the SCN and subordinate clocks along the hypothalamus–pituitary–adrenal (HPA) axis [8,9]. The SCN induces corticotropin-releasing hormone (CRH) and arginine vasopressin (AVP) release by the paraventricular nucleus of the hypothalamus (PVN). In turn, the PVN induces the rhythmic secretion of adrenocorticotropic hormone (ACTH) by the pituitary and, consequently, GC production by the adrenal gland. Via autonomic pathways, the SCN–PVN synchronize adrenal clocks by regulating the time-of-day-dependent sensitivity of the adrenal to ACTH stimulation [9]. As a result, GC levels are peaking at the beginning of the active phase to coordinate complex awake functions through the activation of the GC receptor (GR) in a wide variety of tissues [8,9].

In rodents, GR is expressed in the SCN during the perinatal period, thus GCs have direct effects on the SCN clock during this critical period of development [10–12]. In contrast, the adult SCN has been largely considered resistant to GCs due to its low expression of GR [13,14]. Interestingly, experiments challenging the SCN by a shift in the light–dark (LD) cycle (a jet lag paradigm) have shown that peripheral GCs play a key role in the resynchronization of circadian locomotor activity [15–18]. Here, we aimed to explore how the expression of GR changes along development and how GCs may continue to influence the SCN in the adult.

The knowledge about the development of the mouse SCN is relatively limited, it is assumed that the fetal/neonate clock starts to tick at both cellular/circuit and molecular levels during the perinatal period, under the influence of early rhythmic cues (e.g., maternal GCs, melatonin) [19,20]. This period is considered a critical developmental window because maternal circadian disruptions (e.g., exposure to an altered photoperiod, sleep deprivation, miss-timed stress) can negatively impact the offspring’s physiological fitness later in life [10,21–24]. The SCN develops from undifferentiated cells expressing clock genes with low amplitudes and with weak intercellular synchrony [25] to a highly differentiated, interconnected, and synchronized multicellular network around the time when afferent retinal projections reach the SCN and the eyes open (postnatal day (PND) 10) [26]. By PND30, the SCN circuit reaches adult-like features (i.e.,: heterogeneous cell composition, anatomical organization, mature input/output connections) [27,28]. The adult SCN is formed by ca. 20,000 mainly GABAergic neurons that exhibit precise and high-amplitude circadian cycles of gene expression and synaptic communication. The circuit is also characterized by extensive paracrine communication mediated by neuropeptides including vasoactive intestinal polypeptide (VIP) and AVP, among many others, which are believed to confer the network-level properties necessary to maintain robust oscillations [5]. Astrocytes are an integral part of the SCN as well, with a cellular abundance estimated at roughly one fourth that of the neurons [27–29]. Astrocytes interact tightly with neurons to modulate the rhythmic output of the central circadian pacemaker [30–36].

Since GR expression changes along the development of the SCN, we hypothesize that the nature of the influence that GCs have on circadian pacemaking also changes. In mice prenatally exposed to corticosterone (CORT, the main GC in rodents) out-of-phase compared to the maternal physiological rhythms, we found that the re-adaption of locomotor activity rhythms during simulated jet lag was slower compared to naïve mice and to mice exposed to the same CORT dose, but in-phase. The re-adaptation to a shift in the LD cycle, depends on the interaction along the SCN–HPA axis and on the homeostasis of GC, which is altered in these mice [10] and in similar models of early GC exposure [37,38]. Interestingly, when assessing the expression of GR in the SCN, we found a downregulation in mice exposed to prenatal CORT out-of-phase. Importantly, astrocytes seem to be the main contributors to this difference between groups. To further understand how GR expression changes along SCN development and whether it is modulated in a cell type-specific manner, we followed the maturation of the mouse SCN at single-cell level. We found that GR is expressed in all SCN cells early in development while it becomes more pronounced in the astrocyte population in the adult. We then show that in the adult SCN, GR responds to circulating GCs and that the communication between astrocytes is involved in the effects of the hormone. Our findings provide a conceptual advance on how the central clock develops and on how GCs influence the SCN during the prenatal period and later in adult life, essential to understand how the clock keeps time.

## Results

### Mice prenatally exposed to CORT out-of-phase re-adapt slower to a shift in the LD cycle

**Fig 1:**
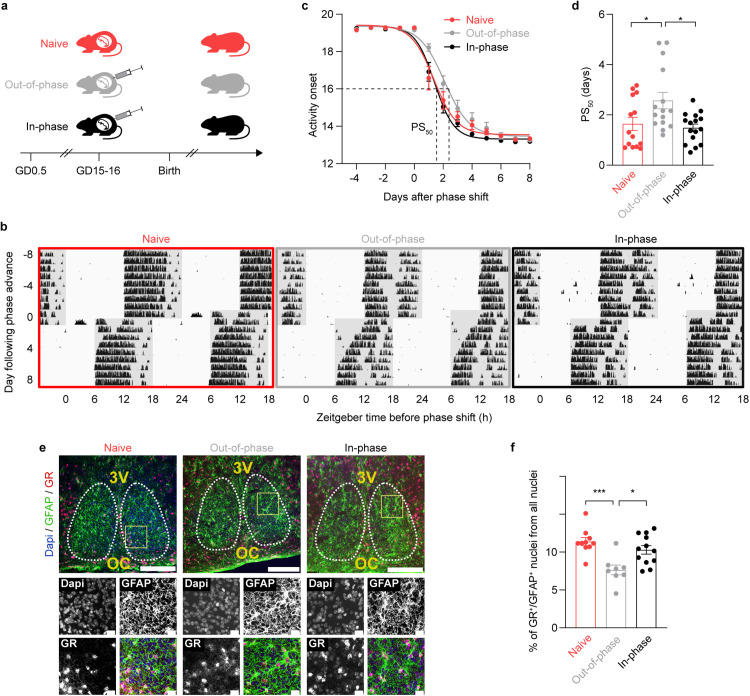
Mice prenatally exposed to CORT out-of-phase re-adapt slower to a shift in the LD cycle. a) Scheme of the gestational intervention, pregnant mice received subcutaneous (s.c.) injections of corticosterone (CORT) at Zeitgeber time 0 (ZT0, out-of-phase) or at ZT12 (in-phase) on gestational days (GD) 15 and 16. A naive group of adult mice whose mothers were left undisturbed during their whole pregnancy was included and the effects on the offspring’s re-adaptation after a 6-hour phase advance in the LD cycle (jet lag) were assessed from postnatal day 60–80. **b)** Representative double-plotted running wheel actograms before and after jet lag, dark phase is shaded in gray. **c)** Average of the activity onset time before and after the phase-shift, dotted line indicate phase-shift 50 (PS_50_) defined as the time at which half of the phase shift is completed (naïve offspring (*n* = 14, 8 males and 6 females), out-of-phase offspring (*n* = 15, 7 males and 8 females) and in-phase group (*n* = 16, 8 males and 8 females). **d)** For PS_50_ comparisons between groups, data are expressed as mean ± SEM, passed normality test and statistical difference was assessed by one-way ANOVA, *F*(2,42) = 5.382, *p* = 0.0083, followed by Holm–Sidak’s multiple-comparison test (naïve vs. out-of-phase, *p = 0.0291, out-of-phase vs. in-phase, *p = 0.0114). **e)** Representative double immunohistochemistry (GR and GFAP) confocal pictures. The dotted white regions demarcate the SCN and the magnified area is indicated with yellow squares, scalebars correspond to 100 µm, except for the zoomed images, where it is 25 µm. **f)** Quantification of the expression of GR specifically in SCN astrocytes. Data are expressed as mean ± SEM (*n* = 8–12/group), passed normality test and statistical difference was assessed by one-way ANOVA, *F*(2,27) = 9.648, *p* = 0.0007, followed by Holm–Sidak’s multiple comparison test (naïve vs. out-of-phase, ***p = 0.0005, out-of-phase vs. in-phase, *p = 0.0104). Numerical data can be found in S1 Data file.

Previous evidence from our lab showed that the maternal exposure to prenatal GCs at the “wrong” time of day (i.e., out-of-phase, misaligned to the maternal physiological rhythms) altered the offsprings’ GCs homeostasis [10]. Interestingly, if the mother is exposed to the same CORT dose but at the beginning of the active phase (i.e., in-phase, aligned to the maternal physiological rhythms), the offspring behavior is comparable to that of naïve mice, whose mothers were undisturbed during pregnancy [10]. Since the out-of-phase offspring produced overall higher levels of GCs [10] and the resynchronization after jet lag depends on the levels and circadian phase of adrenal GCs [18,39], we asked whether the re-entrainment capacity to a 6-hour phase advance in the LD cycle (jet lag) would be affected in our model (Fig 1a). The representative actograms show the re-adaption of the locomotor activity onset during jet lag in adult mice exposed prenatally to CORT in- and out-of-phase and age-matched naive mice (Figs 1b and S1). To quantify the speed of re-entrainment, we calculated phase-shift 50 (PS_50_), defined as the time at which half of the 6-hour phase shift is completed. We observed that the kinetics of the re-adaptation to the new LD cycle was slower in out-of-phase exposed mice compared to in-phase and naive mice (Fig 1c and 1d). This slower re-adaptation did not seem to depend on a weaker entrainment to the LD prior to the shift (S1a–S1c Fig) or on the sex of the mice (S1d Fig). Interestingly, in mice prenatally exposed to CORT out-of-phase, we found a reduced expression of GR in the SCN. Astrocytes seem to be the main contributor of this difference (Figs 1e, 1f, and S1e).

These data suggest that the exposure to CORT out-of-phase during a critical window of development has long-term consequences on the expression of GR in SCN astrocytes and on the resynchronization during jet lag. While the SCN sensitivity to GCs has been already shown during the perinatal period in rodents [10–12], the adult SCN has been considered resistant to GCs [12–14]. Importantly, the activity/rest patterns are not affected by the injection/manipulation at different times of day suggesting that CORT is the main contributor to the offspring’s phenotype (S2 Fig and S1 Data). To understand our observation in this context, we decided to follow the expression of GR along SCN development at single-cell level.

### Single-nuclei transcriptional profiling of the SCN along maturation

**Fig 2:**
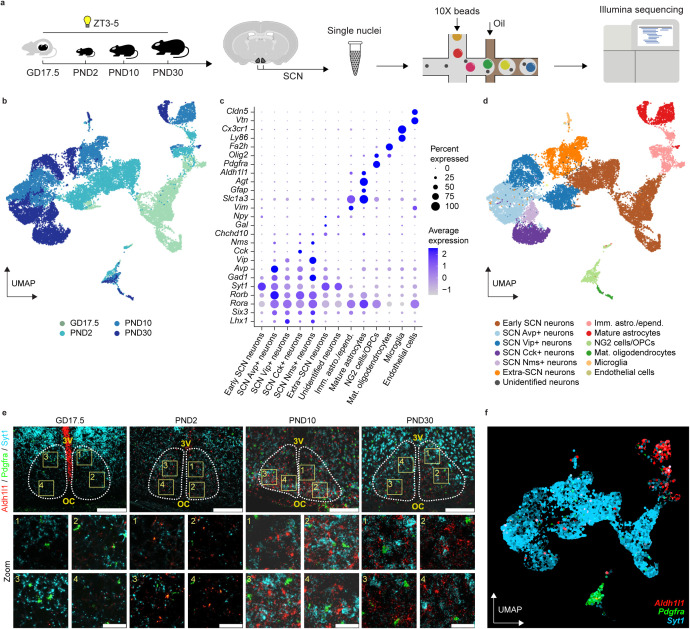
Single-nuclei transcriptional profiling of the SCN along maturation. a) Overview of the experimental workflow. SCN was dissected from 5 male and 5 female fetuses/newborns at three key developmental stages (GD17.5, PND2, PND10) and from adults at PND30 when the SCN reach adult-like maturity at ZT3-5 (3–5 hours after “lights on”). Single-nuclei RNA-sequencing was performed on the pooled extracted nuclei suspensions. **b)** UMAP embedding colored based on developmental stage. **c)** Dot plot showing expression of the markers used to identify the main clusters. **d)** Same UMAP embedding as shown in b but colored based on main cluster annotations using markers in c. **e)** Representative confocal images of in situ hybridization to detect markers of the main cell clusters (*Pdgfra*, NG2 cells/OPCs/oligodendrocytes, green; *Syt1*, Neurons/neuronal progenitors, cyan; *Aldh1l1*, mature/immature astrocytes and ependymal cells, red). The dotted white regions demarcate the SCN and the yellow squares the four magnified areas; scale bars on top and bottom pictures correspond to 100 µm and 20 µm, respectively. OC, optic chiasm, 3V, third ventricle. **f)** Same UMAP embedding as in b and d, but colored for expression of *Pdgfra* (green), *Syt1* (cyan), and *Aldh1l1* (red). Raw transcriptomic data can be found under GEO accession number GSE240803.

We performed single-nuclei RNA sequencing (snRNA-seq) of micro-dissected mouse SCN at three key developmental stages and at maturity. The developmental stages were selected to match milestone events during the maturation of the mouse SCN that were reported in the literature [25,40]: the neurogenic-gliogenic switch (gestational day (GD) 17.5) [41], the peak of astrocyte proliferation (PND2) [42], the maturation of the main photic input pathway, the retinohypothalamic tract (PND10) [26,43,44] and the adult-like mature circuit (PND30) [27,28]. To generate a comprehensive transcriptomic data set of SCN cells, we dissected brains from male and female mice between ZT 3 and 5 (i.e., *Zeitgeber* time, 3–5 hours after “lights on”) and prepared 250-μm thick coronal sections to precisely identify the SCN under the microscope (Fig 2a, S3a, and S3b). To visualize the transcriptional signature of the SCN during development, we generated an UMAP embedding colored by timepoints (Fig 2b), which shows that the SCN gains transcriptional heterogeneity with maturation. We annotated 13 main clusters based on known molecular markers in the developing anterior hypothalamus and the mature SCN [45–48] (Fig 2c and 2d). These clusters were named according to their association with cell lineages: neuronal (*Avp*^*+*^, *Vip*^*+*^, *Cck*^*+*^, and *Nms*^*+*^ mature neurons, early SCN neurons, and extra-SCN neurons), astroglial (immature/ependymal cells and mature astrocytes), oligodendroglial (NG2 cells/oligodendrocyte precursor cells (OPCs) and mature oligodendrocytes), microglial, and endothelial cells. We were unable to identify a small cluster of neuronal cells (79 cells) mainly present at GD17.5, PND2 and PND10 (Figs 2c, 2d, and S5a). Even though it is possible that certain clusters are under represented due to the gene expression levels at the sample collection time point (ZT 3–5), the total number of cells per cluster that we identified at PND30 fits with the expected proportion of cell types in the adult SCN (i.e., a neuron:astrocyte ratio of 3:1, around 5% of NG2 cells/oligodendrocytes and below 2%–3% of endothelial cells and microglia) [27–29,45]. We further validated the presence of cell types corresponding to the main clusters by in situ hybridization on age-matched mouse brain sections. Representative confocal images and snRNA-seq gene expression plots (Fig 2e and 2f) show the main cell clusters: oligodendroglial lineage (*Pdgfra,* green), neuronal lineage (*Syt1*, cyan) and astroglial lineage (*Aldh1l1*, red). These marker genes were chosen to validate the main clusters because they are expressed across all developmental stages.

Overall, our data show that during maturation, the SCN gradually gains cellular and transcriptional heterogeneity and that distinct transcriptional signatures define developmental stages, corresponding to the previously described temporal window of SCN circuit maturation. Therefore, we used this data set to follow cell type-specific changes of glucocorticoid receptor *Gr* (*Nr3c1)* expression along the development of the SCN.

### Cell-type-specific changes in *Gr* expression along SCN maturation

**Fig 3:**
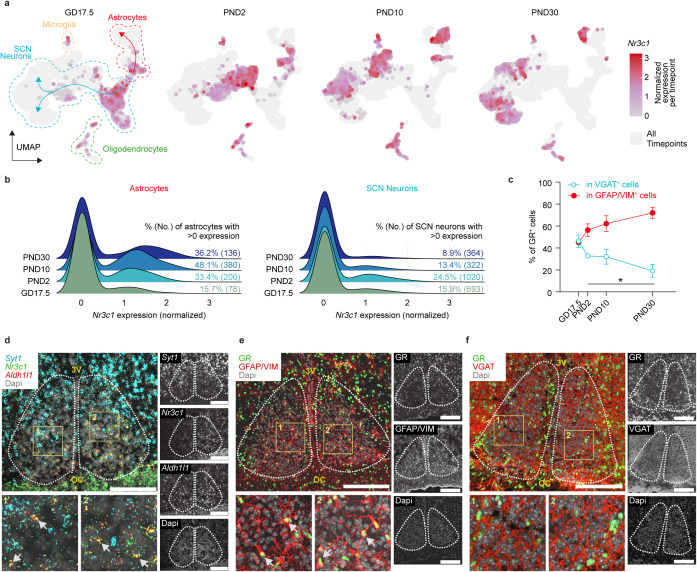
Cell-type-specific changes in *Gr* expression along SCN maturation. a) Expression of Gr in the snRNA-seq dataset in the same UMAP embedding as Fig 2b across different developmental stages including all cell clusters. Cells across all time points including those that do not expressed are shown in the background in light gray. **b)** Density plots of the *Gr* expression in astrocytes and SCN neurons segregated by developmental stage. The percentage (and absolute numbers) of cells with non-zero expression of *Gr* are indicated for each cell cluster. Note that since the total number of cells was different across the time points, the cell numbers have been normalized to a total of 5,000 cells per developmental time point. **c)** The percentage of GR^+^ cells co-expressing astrocytic markers (GFAP/VIM^+^ in red) or neuronal marker (VGAT^+^ in cyan) are plotted for each developmental stage as mean ± SEM (*n* = 4–6 independent samples). Statistical differences were assessed by multi-comparison Mann–Whitney test, * *p* < 0.05. **d)** Representative in situ hybridization confocal images showing expression of *Syt1* (cyan), *Gr* (green), and *Aldh1l1* (red) of the mouse SCN at PND30. White arrows highlight the co-expression of *Gr* and *Aldh1l1*. **e)** Representative immunohistochemistry confocal images of the SCN (DAPI), for GR (green) in astrocytes (GFAP/VIM^+^; red) of the mouse SCN at PND30. White arrows highlight the co-expression of GR and GFAP/VIM. **f)** Representative immunohistochemistry confocal images of the SCN (DAPI), for GR (green) in neurons (VGAT; red) of the mouse SCN at PND30. In d–f, the dotted white regions demarcate the SCN and the two yellow squares, the magnified areas. Scalebars correspond to 200 µm, except for the zoomed images, where it is 20 µm. OC, optic chiasm, 3V, third ventricle. Raw transcriptomic data can be found under GEO accession number GSE240803, numerical data can be found in S1 Data file.

Interestingly, the data displayed in Fig 3a and quantified in Fig 3b, show that *Gr* is expressed in most clusters at GD17.5 and PND2, but later, at PND10 and PND30, the expression becomes more pronounced in astrocytes. Although, microglia and oligodendrocyte clusters also express the receptor, they make up a small percentage of all SCN cells. In Fig 3b, the second peak corresponding to cells with non-zero expression increases in magnitude (indicating higher number of cells with non-zero *Gr* expression) and shifts to the right (indicating increased *Gr* expression) in the case of astrocytes, while it displays an opposite trend in SCN neurons. Interestingly, at PND10 the absolute number of astrocytes expressing *Gr* exceeds the number of neurons expressing the gene, despite there being roughly three times as many neurons as there are astrocytes in the SCN from this developmental time point on. In comparison, at PND30, while the absolute number of astrocytes expressing *Gr* is lower than that of neurons, astrocytes express it at higher levels. Density plots also show the expression of *Gr* in microglia and mature oligodendrocytes clusters (S6a and S6b Fig). Although the expression of *Gr* during perinatal development was already reported [10–12], here we show that *Gr* expression is not simply down-regulated as the SCN matures, but rather modulated in a cell type-specific manner. To quantify this observation, we performed a trajectory analysis on our snRNA-seq data, which also indicated an up-regulation of *Gr* expression in astrocytes along developmental pseudo-time (S6c and S6d Fig). To assess this developmental trend at the protein level, we performed a co-staining of GR with markers of both, mature and immature astrocytes (expressing GFAP and vimentin, VIM) and neurons (expressing the vesicular GABA transporter, VGAT) (S7 Fig). Quantification shows that the number of cells expressing GR co-stained with astrocytic markers tend to increase (Figs 3c and S7a) while those co-stained with VGAT tend to become less frequent with age (Figs 3c and S7b). Although we cannot compare the transcriptional and immunohistochemical data in a quantitative way, the observed developmental changes follow a similar trend with both approaches (Figs 3c and S6e).

We then assessed whether the up-regulation of *Gr* expression in astrocytes was aligned with a greater activation of GR-dependent downstream pathways. Transcripts associated with the GO biological process term *Cellular response to glucocorticoid stimulus* were maintained at low levels in neurons, while there was a tendency for increased enrichment in astrocytes along SCN maturation (S6f Fig). Overall, our data suggest that throughout development the expression of *Gr* in the SCN is modulated depending on the cell type. This finding suggests that the SCN could remain responsive to GCs in the adult stage.

### Adult SCN astrocytes respond to circulating GCs in vivo

**Fig 4:**
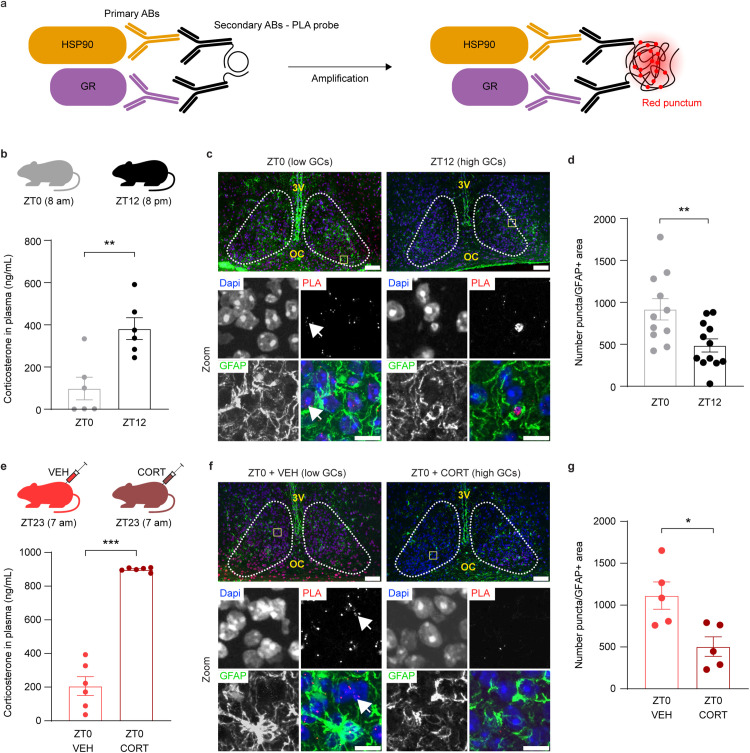
Adult SCN astrocytes respond to circulating GCs in vivo. a) Schematic showing proximity ligation assay (PLA) setup. b) Corticosterone levels (in ng/mL) in plasma of mice kept in 12-hour:12-hour light:dark (LD) conditions at ZT0 (lights on) and ZT12 (lights off). Data are expressed as mean ± SEM (*n* = 6 mice/group), passed normality test and statistical difference was assessed by two-tailed *T* test, *t* = 3.801, df = 10, **p = 0.0035. **c)** Proximity ligation assay in the SCN showing the interaction between HSP90 and GR (PLA puncta), in astrocytes (GFAP+) with nuclei counterstained with DAPI. Top: reconstruction of the SCN from pictures taken at 40× magnification (scale bar 50 µm). Bottom: zoom in all channels separately and merged (scale bar 10 µm). The dotted white regions demarcate the SCN and the magnified areas are indicated with yellow squares, arrows highlight PLA puncta. **d)** Quantification of the number of GR-HSP90 signal (puncta)/GFAP^+^ area at both timepoints. Data are expressed as mean ± SEM (1 section/animal, 11–12 mice/time point), passed normality test and statistical difference was assessed by two-tailed *T* test, *t* = 2.969, df = 21, **p = 0.0073. **e)** Corticosterone levels (in ng/mL) in plasma of mice kept in 12-hour:12-hour light:dark (LD) conditions at ZT0, one hour after subcutaneous injection either with VEH or CORT (5 mg/Kg b.w. at ZT23). Data are expressed as mean ± SEM (*n* = 6 mice/group), passed normality test and statistical difference was assessed by two-tailed *T* test, *t* = 12.35, df = 10, ***p < 0.0001. **f)** Proximity ligation assay in the SCN showing the interaction between HSP90 and GR (PLA puncta), in astrocytes (GFAP^+^) with nuclei counterstained with DAPI. Top: reconstruction of the SCN from pictures taken at 40× magnification (scale bar 50 µm). Bottom: zoom in all channels separately and merged (scale bar 10 µm). The dotted white regions demarcate the SCN and the magnified areas are indicated with yellow squares, arrows highlight PLA puncta. **g)** Quantification of the number of GR-HSP90 signal (puncta)/GFAP^+^ area at both timepoints. Data are expressed as mean ± SEM (1 section/animal, 5 mice/group), passed normality test and statistical difference was assessed by two-tailed *T* test, *t* = 3.036, df = 8, *p = 0.0162. Numerical data can be found in S1 Data file.

To follow-up on a potential responsiveness to GCs by the adult SCN, we further confirmed by in situ hybridization at PND30 the presence of *Gr* transcript in astrocytes labeled with *Aldh1l1* probes in red (showing co-expression, white arrows). However, we did not observe co-expression with *Syt1* probes, a gene exclusively expressed in neurons (Fig 3d). At the protein level, we found a significantly higher percentage of GR^+^ cells co-expressing the astrocytic markers GFAP and VIM (Fig 3e) than co-expressing the neuronal marker VGAT (Fig 3f). To evaluate whether GR was responsive to circadian GC changes in the SCN, we used a proximity ligation assay (PLA) to visualize GR activation in vivo in PND30 mice [49]. HSP90 is a well-known chaperone protein that binds inactive GR in the cytosol, and this interaction can be visualized with PLA (Fig 4a) [49,50]. We reasoned that at ZT0 (*Zeitgeber* time 0, time of day when the lights were switched on) the GC levels are low (Fig 4b) and the inactive GR would be bound to HSP90 (i.e., maximal interaction generating maximal signal). In contrast, at ZT12 (*Zeitgeber* time 12, time of day when the lights were switched off) murine GC levels are at their physiological peak (Fig 4b) and active GR would not be bound to HSP90 (i.e., minimal interaction generating minimal signal). We coupled immunofluorescence against GFAP with PLA to quantify GR-HSP90 interaction in GFAP+ astrocytes on brain sections from mice at ZT0 and ZT12. We observed a significant difference in the PLA signal (red puncta, white arrow) detected in SCN GFAP+ astrocytes between ZT0 and ZT12 (Fig 4c and 4d) indicating that GR in SCN astrocytes is activated by circadian GC levels. As a positive control and to validate this approach, we compared PLA signals in the PVN, a component of the HPA axis that strongly responds to GC feedback, finding similar effects (S9a and S9c Fig). To further confirm that GR in the SCN responds to circulating GC changes, we performed the same experiment in brain sections from mice obtained at ZT0, but one hour after subcutaneous injection with either vehicle (VEH, PEG-400 50% in PBS) or corticosterone (CORT, 5 mg/kg of b.w.). We monitored plasma corticosterone levels and found low and high GC levels in the VEH and CORT groups, respectively (Fig 4e). We detected a significantly higher PLA signal in SCN GFAP+ astrocytes at ZT0-VEH when compared to ZT0-CORT (Fig 4f and 4g).

**Fig 5:**
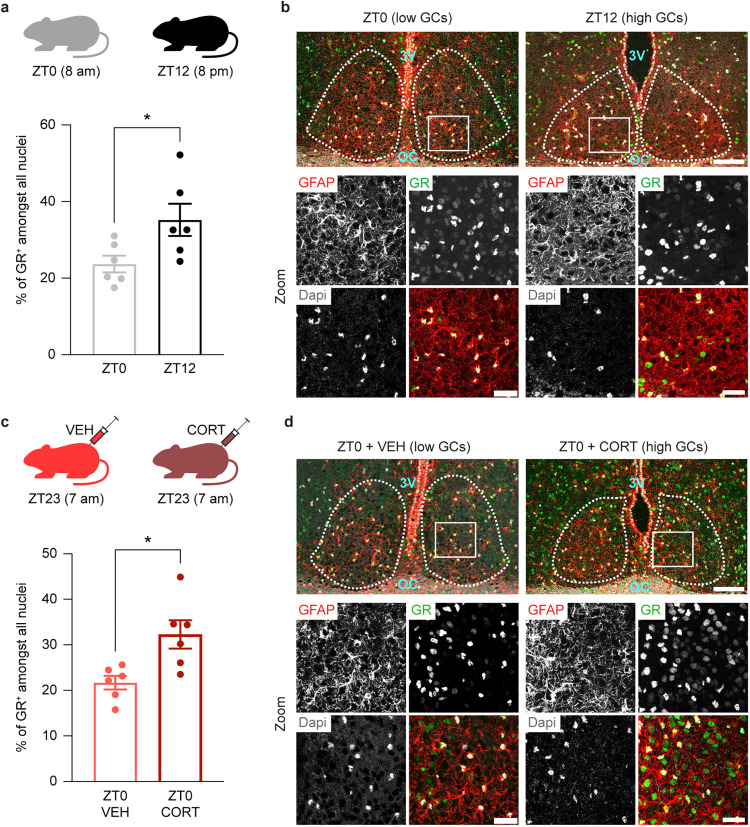
SCN astrocytes express GR and respond to circulating GCs in vivo. a) Quantification of active GR in the nuclei at both ZT0 (low circulating GCs) and ZT12 (high GCs). Data are expressed as mean ± SEM (1 section/animal, *n* = 6 mice/time point), passed normality test and statistical difference was assessed by two-tailed *T* test, *t* = 2.43, df = 10, *p = 0.035. **b)** Representative confocal pictures of the SCN with a triple immunostaining for GR (green), GFAP (red), and SOX9 (gray). Overview of the SCN from pictures taken at 20× magnification (scale bar 100 µm) and zoomed in (scale bar 25 µm). The dotted white regions demarcate the SCN, and the white squares indicate the zoomed areas. **c)** Quantification of active GR (in the nuclei) at ZT0 1 hour after subcutaneous injection with either vehicle (VEH, PEG-400 50% in PBS) or corticosterone (CORT 5 mg/kg b.w.) at ZT23. Data are expressed as mean ± SEM (1 section/animal, *n* = 6 mice/group), passed normality test and statistical difference was assessed by two-tailed *T* test, *t* = 3.07, df = 10, *p = 0.012. **d)** Representative confocal pictures of the SCN with a triple immunostaining for GR (green), GFAP (red), and SOX9 (gray). Overview of the SCN from pictures taken at 20× magnification (scale bar 100 µm) and zoomed in (scale bar 25 µm). The dotted white regions demarcate the SCN, and the white squares indicate the zoomed areas. Numerical data can be found in S1 Data file.

Since we also observed the above-described changes in GFAP− areas (S9d and S9e Fig), we could not confirm astrocytic-specific responses with this approach. This could be due to other SCN cells expressing GR that respond to peripheral GCs or, alternatively, to the fact that not all SCN astrocytes express GFAP. We discarded the first option because in our transcriptional data (at PND30), we found that only around 9% of SCN cells express *Gr* and no astrocytic markers. On the other hand, we also found in our transcriptional data that only around 37% of the cells clustered as SCN astrocytes expressed *Gfap* (S9g Fig). Therefore, we decided to assess the activation of GR in the SCN by a second approach that allows us to capture a higher proportion of astrocytes. We assessed the percentage of nuclear GR (active) by triple immunohistochemistry combining two antibodies to label SCN astrocytes. The combination of GFAP and SRY-Box transcription factor 9 (SOX9) allowed us to detect more of these cells (S9g Fig). With this approach we found a significantly higher percentage of GR+ nuclei at ZT12 (Fig 5a and 5b) and at ZT0, 1 hour after CORT injection (Fig 5c and 5d) compared to respective controls. On average, around 85% of the detected GR^+^ nuclei co-stained with either GFAP or SOX9 or both astrocytic makers (S9f Fig). These data align well with the PLA suggesting that GR is activated by circulating GCs in the SCN with a major contribution, although not exclusively, from astrocytes.

Finally, we sought to assess in previously published snRNA-seq data sets, whether the expression of GR related transcripts shows circadian changes in a cell type-specific manner in the adult SCN. We selected two data sets: Morris *and colleagues*, 2021 [45], generated from PND10 to 12 mouse SCN organotypic explants in culture and collected at two timepoints, circadian time (CT) 7.5 (i.e., when low physiological GC levels are expected) and CT15.5 (i.e., when high GC levels are expected) [45]. Wen *and colleagues*, 2020 [28], generated from SCN samples obtained from PND56 mouse at different circadian timepoints, at which we analyzed (CT26 and CT50; i.e., at low circulating GC levels, and CT14 and CT38; i.e., at high circulating GC levels). First, we annotated the clusters based on known cell types in the mature SCN (S10a and S10d Fig), we then ran a single-sample gene set enrichment analysis to compare the enrichment of transcripts in each cell type at low- and high-GC conditions (both, expected and present). We could statistically confirm an enrichment of genes associated with the GO molecular function term *GR binding* in SCN astrocytes, ependymal cells, and in extra-SCN neurons when high GCs levels are expected (S10b Fig) in the Morris *and colleagues* data set. We also found an enrichment of genes associated with the GO molecular function term *GR pathway* in SCN astrocytes, ependymal cells and in extra-SCN neurons when circulating GCs are high in the Wen *and colleagues* data set (S10e Fig). Moreover, we observed a significant enrichment of ligand-receptor (LR) pairs involved in extracellular matrix remodeling (e.g., *Angptl4* -> *Sdc2-4*) and cholesterol uptake (e.g., *Apoe* -> *Scarb1*) in SCN astrocytes, when we restricted LRs to GR-regulon member genes (S10c Fig). These data could potentially explain previous observations suggesting that the structural plasticity of SCN astrocytes changes in a circadian manner and it is influenced by circulating GCs [51–55]. Interestingly, the comparative analysis of both data sets suggest that even in the absence of circulating GCs the SCN still expects to receive the signal. This could be a feature of a daily program of the SCN that requires further investigation. Overall, our data show that adult SCN astrocytes can sense circulating GCs through GR activation. Thus, we further explored the effect of GCs on SCN astrocytes.

### Calcium signaling is activated by GCs in SCN astrocytes

**Fig 6:**
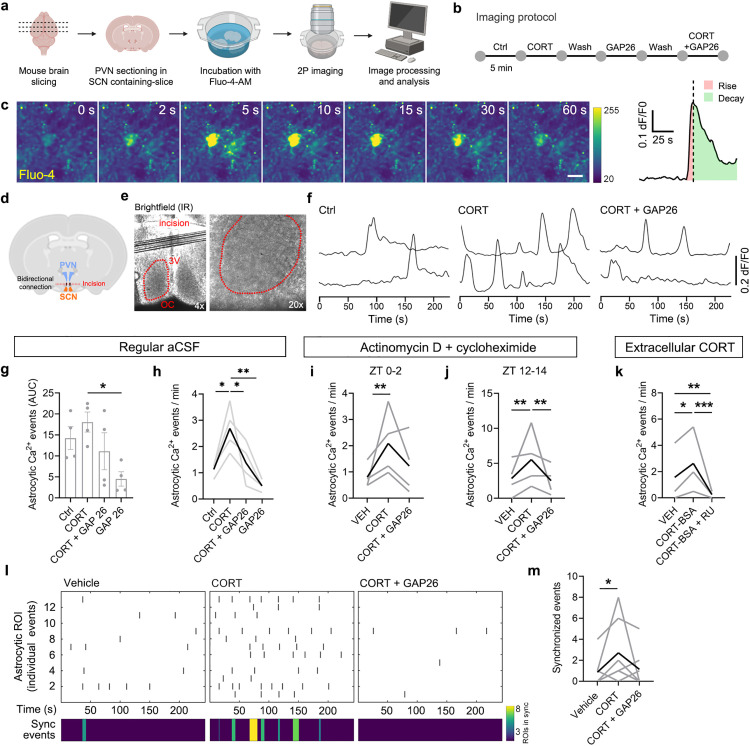
Calcium signaling is activated by GCs in SCN astrocytes. a) Overview of the experimental workflow. *Created in BioRender. Administrator,* S. *(2026)* https://BioRender.com/ql4wtok*.* **b)** Imaging protocol depicting the sequence of treatments performed to the same slice. Ca^2+^ signal was quantified in Fluo-4-stained SCN slices perfused at 0.5 mL/min with oxygenated aCSF at 37 °C. Pharmacological treatments were done perfusing the solutions to the recording chamber at the following working concentrations (in aCSF): 1 μM CORT, 400 μM GAP26, or the mixture of the two, a 5-min wash period was done in between. Baseline (control, Ctrl) and vehicle (VEH) recordings were done at the beginning and at the end (recovery) and the Z-plane was maintained between recordings. **c)** Representative image of a typical astrocytic Ca^2+^ event, identified according to the following criteria: a typical astrocytic morphology, a duration of at least 10 s and a signal decay longer than the signal rise. **d)** Schematic of how the connection between the SCN and the PVN was interrupted with a scalpel cut between both regions and perpendicular to the third ventricle (https://biorender.com/ql4wtok). **e)** Representative image of the localization of the SCN and the SCN–PVN incision in the acute slice imaged in brightfield with infrared illumination. **f)** Representative traces of the three conditions (Control, CORT, and CORT + GAP26) with examples of calcium transients. **g)** Integrated Ca^2+^ response represented as the area under the curve (AUC) (independent experiments, *n* = 4 mice), data passed normality test and statistical differences were assessed by repeated-measures one-way ANOVA (*F*(3,9) = 3.941, *p* = 0.0476) followed by Holm–Sidak’s multiple comparison test (CORT vs. GAP26, *p = 0.052). **h)** Number of astrocytic Ca^2+^ events is represented (independent experiments, *n* = 4 mice, in gray) and as mean (in black), data passed normality test and statistical differences were assessed by repeated measures one-way ANOVA (*F*(3,9) = 9.847, *p* = 0.0033) followed by Holm–Sidak’s multiple comparison test (Ctrl vs. CORT, *p = 0.022; CORT vs. CORT + GAP26, *p = 0.0451; CORT vs. GAP26, **p = 0.0031). SCN slices from g and h were prepared from mice at ZT0-2 and incubated before and during the recording with regular aCSF. **i)** Number of astrocytic Ca^2+^ events is represented (independent experiments, *n* = 4 mice, in gray) and as mean (in black). SCN slices were prepared from mice at ZT0-2 and incubated before and during the recording in aCSF containing actinomycin D (a transcription inhibitor) and cycloheximide (a protein synthesis inhibitor). Data were analyzed by a negative binomial generalized linear model to assess statistical differences between the treatments accounting for ROIs nested within slices (animals), VEH vs. CORT, **p = 0.0027. **j)** Number of astrocytic Ca^2+^ events is represented (independent experiments, *n* = 4 mice, in gray) and as mean (in black). SCN slices were prepared from mice at ZT12-14 and incubated before and during the recording in aCSF containing actinomycin D and cycloheximide. Data were analyzed by a negative binomial generalized linear model, VEH vs. CORT, **p = 0.0028, CORT vs. CORT + GAP26, **p = 0.0001. **k)** Number of astrocytic Ca^2+^ events is represented (independent experiments, *n* = 3 mice, in gray) and as mean (in black). SCN slices were prepared from mice at ZT0-2 and with CORT-BSA followed by the GR antagonist RU486 in regular aCSF. Data were analyzed by a negative binomial generalized linear model, VEH vs. CORT-BSA, *p = 0.028, CORT-BSA vs. CORT-BSA + RU, ***p = 0.00042 and VEH vs. CORT-BSA + RU, **p = 0.021. **l)** Representation of all Ca^2+^ transients from astrocytic-like ROIs were followed during the 200 s of recording while perfusing VEH, CORT and CORT + GAP26 to the SCN slice. Heatmaps show a temporal sequence of activation with color indicating the number of ROIs that fired in synchrony. **m)** Number of synchronous Ca^2+^ events across all treatments. Each gray line represents one independent experiment showing synchronyc events (*n* = 7) and the mean is shown in black. All treatments were performed on the same slice. Poisson generalized linear mixed-effects model detects a significant increase in synchronic events during CORT treatment (*p = 0.037), the offset model revealed no significant effect of the treatment on the synchronization rate (Wald *χ*² = 1.00, df = 2, *p* = 0.608). Numerical data can be found in S1 Data file.

We monitored calcium transients during CORT treatment in acute SCN slices loaded with the cell-permeable calcium sensor Fluo-4-AM. We imaged fluorescence emission, as a readout of intracellular calcium release, using two-photon imaging and then performed image analysis to evaluate astrocytic calcium events (Fig 6a). All pharmacological treatments were done sequentially by perfusing the solutions through the recording chamber. Baseline (Ctrl) and vehicle (VEH, aCSF with 0.1% DMSO) recordings were done first, followed by 5-min recordings during treatment with: 1 μM CORT, 400 μM GAP26 and a mixture of the two. GAP26 blocks hemichannels as well as intercellular communication through gap junctions specifically containing connexin 43 (Cn43) [56,57]. GAP26 was the inhibitor of choice to prevent astrocyte–astrocyte communication, because Cn43 is expressed in astrocytes in the SCN (S11a Fig) [32,58–60]. Since we found that circulating GCs activate GR in the SCN with an important contribution of astrocytes, we used step-by-step selection criteria to quantify the calcium transients from astrocyte-like regions of interest (ROIs) [61–65]. In a first step, we distinguished Fluo-4-loaded astrocyte-like cells based on their morphology (i.e., cells with small soma and complex radial arborizations) (S11b Fig) [63]. In a second step, we filtered only those Ca^2+^ events longer than 10 s that are typically observed in the astrocytic soma and main processes (Fig 6c and S1 Data) [66]. In a third step, we further filtered events with signal decay longer than the signal rise, a well-described feature of Ca^2+^ events in astrocytes (Fig 6c and S1 video) [63,66–68]. Once all regions of interest were selected, we assessed whether these Ca^2+^ events occurred in synchrony, a unique feature of astrocytic activation. Therefore, faster and sharper events, with a lower decay:rise duration ratio were not included in the analysis and were classified as non-astrocytic events (S11c–S11e Fig and S1 Data). To block a possible indirect astrocytic activation through the connections between the SCN and the PVN, we performed a scalpel cut between both regions, perpendicular to the third ventricle (Figs 6d and S11f–S11h). In Fig 6e, we show a representative image of the localization of the SCN and the SCN–PVN incision in the acute slice imaged in brightfield with infrared illumination.

Representative traces of the three conditions (Ctrl, CORT and CORT + GAP26) showed that CORT treatment increases the number of astrocyte-like Ca^2+^ events in the SCN depending, at least in part, on astrocyte–astrocyte communication through gap junctions (Fig 6f and S1 video). Applying our analysis pipeline, we quantified the integrated Ca^2+^ response to CORT by calculating the area under the curve (AUC) of all detected events. We did not found a significant effect of CORT compared to baseline control (Fig 6g). The total AUC depends on the number of events as well as the duration and amplitude of each of them. While we did not find a significant difference on the duration and the amplitude (S11i and S11j Fig), we found that CORT significantly increased the number of astrocyte-like Ca^2+^ events (Fig 6h). In four independent experiments (in gray lines experiments and in black the mean), we found that the CORT-induced Ca^2+^ events were significantly reduced when astrocyte–astrocyte communication was blocked by addition of GAP26 (Fig 6h). We also found that the total number of active ROIs tended to decrease when CORT binding to the GR was antagonized by co-incubation with the GR antagonist mifepristone (RU486) and that the SCN–PVN incision in the acute slice tended to reduce the duration and the amplitude of all Ca^2+^ events, suggesting that we have reduced the influence from the PVN with our approach (S11f–S11h Fig).

While genomic effects and slow responses were expected, we observed Ca^2+^ events shortly after CORT was perfused into the recording chamber. Then, we sought to understand better how CORT might act on the SCN astrocytes. Therefore, we performed the same sequence of treatments in slices that were incubated before and during the recording with an aCSF containing actinomycin D (a transcription inhibitor which intercalates into DNA, preventing the unwinding of the DNA double-helix and inhibiting the RNA polymerase activity) and cycloheximide (a translation inhibitor which interferes with the translocation step of translation elongation). Applying the same analysis pipeline we quantified the number of active astrocytes in four independent experiments (in gray lines experiments and in black the mean) and found a similar result: an increase in the number of astrocytic Ca^2+^ events with CORT treatment which was significantly reduced when astrocytic communication was blocked with GAP26 (Figs 6i and S11l). These data suggest that transcription and translation are not necessary for the rapid effects of CORT. Moreover, we performed the same sequence of recordings in slices obtained at ZT12–14 to assess CORT effects at a more physiologically relevant time of day. Again, applying the same analysis pipeline we found a similar result (Figs 6j and S11m). Interestingly, we observed that at ZT12–14 the effect of CORT was stronger (i.e., yielding a higher frequency of Ca^2+^ events/min) and more susceptible to the treatment with GAP26. These findings are in line with previous data suggesting that, in mice, astrocytes might be more active during the dark phase [32], when circulating GCs are higher [9] and that the structural plasticity of SCN astrocytes including the expression of Cn43 changes in a circadian manner [51–53,55,58]. These data are also in line with our comparative analysis of published RNA-seq data sets (S10 Fig), strenghthening the idea that adult SCN astrocytes can sense circadian GCs through GR activation. Finally, to check whether CORT might be acting through GR on the cell membrane, we treated the SCN slice with CORT-BSA (corticosterone conjugated with bovine serum albumin) which is unable to enter the cell and found again a similar result: a significant increase in the number of astrocytic Ca^2+^ events with CORT treatment, prevented by the GR antagonist RU486 (Figs 6k and S11n).

In Fig 6l, all Ca^2+^ events from astrocyte-like ROIs were followed during 200 s while perfusing VEH, CORT, and CORT + GAP26 to the SCN slice. Heatmaps show a temporal sequence of activation with the color indicating the number of ROIs that fired in synchrony from a representative experiment. Synchronic events were identified when ≥2 astrocytes exhibited Ca^2+^ events within a 3-second window. When additional active astrocytes were detected in a subsequent 3 s interval, the window expanded iteratively until no new participants were found. Fig 6m shows that from the 8 slices (experiments shown in Fig 6i and 6j), synchronic events were detected in 7 of them. Slices treated with CORT showed significantly more synchronic events (mean ± SEM = 2.71 ± 1.14) than VEH (0.86 ± 0.55, *p* = 0.037). Co-perfusion of GAP26 alongside CORT attenuated this effect (1.14 ± 0.70). However, a Poisson generalized linear mixed-effects model (GLMM) analysis revealed that the rate of synchronized events per total events did not differ significantly across conditions (*χ*² = 1.00, df = 2, *p* = 0.608), suggesting that the increase in synchronized events likely reflects the overall CORT-driven rise in astrocytic calcium activity rather than a specific enhancement of network synchronization. Considering that a key component of astrocytic signaling is the propagation of Ca^2+^ waves within the astroglial syncytia expanding through neighboring cells, the above-mentioned observation further validates the effect of GAP26 as a blocker of astrocyte–astrocyte communication.

Overall, our data suggest that CORT activates SCN astrocytes, increasing the number of Ca^2+^ events in these cells independently of the PVN input. Interestingly, when we specifically blocked a key feature of astrocytic activity, the propagation of Ca^2+^ waves, with GAP26, CORT induced less and more isolated events. Our data also suggest that the effect of CORT is stronger at ZT12, providing a physiologicaly relevant information to the observed effect. Additionally, CORT induced rapid events in the SCN that are independent of transcriptional and translational programs and seem to be due to the activation of GR located at the cell membrane.

## Discussion

Our study presents two main findings: (1) Changes of GR expression along the mouse SCN development showing that the receptor is not simply down-regulated with age. (2) In the adult SCN, astrocytes continue to express GR and respond to CORT.

As the central pacemaker of the circadian system, the SCN integrates timing signals and coordinates the synchrony at the systemic level through neuronal and hormonal pathways. GC production is under circadian control peaking at the beginning of the active phase to coordinate complex awake functions [8,9]. GCs effectively mediate the communication of circadian time between the SCN and peripheral clocks because GR is widely expressed across tissues [69,70]. The strong resetting effect of GCs shifts the phase of the molecular clock machinery and synchronizes the transcriptome of target cells [13]. However, the influence of GCs on the SCN itself remains unclear and seems to depend on the maturity of the circuit.

During the perinatal period, the rodents’ SCN express GR and respond directly to GCs [10–12]. In fetal SCN explants, CORT and other GR agonists entrain the circadian clock, with specific effects on both the period and phase [11,12], suggesting that maternal GCs could provide an essential input signal from the circadian environment to the fetus/neonate [71–73]. Indeed, the perinatal period represents a critical developmental window, it has been shown that maternal circadian disruption (e.g., exposure to an altered photoperiod, sleep deprivation, stress) can negatively impact the offspring’s physiological fitness later in life. We show here that CORT exposure at the “wrong” time of day during late pregnancy (out-of-phase) has long-term consequences on the homeostasis of GCs [10] and on the kinetics of locomotor activity phase-resetting after a 6-hour shift of the LD cycle (jet lag paradigm). Interestingly, offspring prenatally exposed to the same CORT dose but in-phase re-entrain with a speed comparable to naïve mice. Jet lag arises from a transient misalignment between the external time (i.e., light) and the endogenous circadian timing. The SCN gradually drives the resynchronization of locomotor activity to the new LD cycle, where the speed of this process is influenced by the levels and circadian phase of GCs [16–18]. We previously showed that prenatal CORT exposure out-of-phase alters the offspring’s GC homeostasis (i.e., showing higher GC levels during the rest phase, impaired negative feedback, and lower expression of GR in the PVN [10]). Here, we found that these mice also express lower levels of GR in the SCN, with astrocytes being the main contributors to this difference. To contextualize our observation, we followed the expression of GR along SCN development at single-cell level.

We described and validated developmental changes in GR expression along SCN maturation. While most SCN cells express GR early during development, the expression becomes more prominent in the astrocytic cluster after PND10. Previous studies have found little or no expression of the receptor in the adult SCN [13,14]. However, its presence in astrocytes (i.e., in only a fourth of the 20,000 SCN cells [27–29]) likely explains why the expression of *Gr* has been found to be so low in previous in situ hybridization studies [13]. Additionally, the detection of the GR protein proved to be easier when the receptor is active and localized in the nuclei, as we show here. We believe that the dispersed distribution of inactive GR in the cells might explain why the expression has been found to be low in previous immunohistochemistry studies [14].

It is possible that early during development (i.e.,: before eye opening) all cells need to express GR because GCs are maternal entrainment signals. However, in the adult circuit, light becomes the predominant time-cue for the SCN, while GCs could provide essential information about the internal synchrony. In the context of the misalignment caused by jet lag, GCs might mediate an organized resetting of the circadian synchrony. Interestingly, our observations of the effects of GCs in the SCN (i.e., maternal entrainment, changes over development, feedback to the SCN and potential involvement on the locomotor activity phase resetting) resembles those described for another circadian hormone, melatonin [74,75]. We reasoned that if astrocytes would respond to peripheral GCs, they might be in a unique position to integrate (i.e., compute) different inputs within the circuit (i.e., GCs and light), such as during circadian misalignment. Their morphology, their distribution, their capacity to respond to circulating signals and their extensive connection through gap junctions [32,76–78] make them ideal modulators of individual synapses and, importantly, long-range integrators of various signals within the SCN circuit [79]. With that in mind we explored the possibility that the SCN could remain responsive to GCs in the adult and that astrocytes could mediate the effects.

In acute slices from adult mice, we observed that CORT activates SCN astrocytes, increasing the number of Ca^2+^ events. Interestingly, when we specifically blocked a key feature of astrocytic activity, the propagation of Ca^2+^ waves through gap junctions containing Cn43, CORT induced less and more isolated events. Effects of CORT and other GCs have been shown in astrocytes from other brain regions both in vitro [80,81] and in vivo [82]. While genomic effects and slow responses were expected, we observed responses in the range of minutes. In neurons, rapid GC actions have been described in excitatory/inhibitory pre- and post-synapses, through voltage-gated calcium and potassium channels or indirectly though the cannabinoid pathway [reviewerd by 83]. In astrocytes, these rapid actions are under-studied [80–82]. Therefore, we blocked transcriptional and translational programs and observed that CORT induced Ca^2+^ events in SCN astrocytes. These rapid effects seem to, at least in part, depend on the activation of membrane GR. From the physiological point of view, it is interesting to see that the effect of CORT is stronger at ZT12 when the SCN expects higher concentrations of circulating GCs, when its neuronal activity is low and astrocytic activity high [32,33]. Although we are showing that adult SCN astrocytes indeed express GR and are able to sense and respond to GCs, the interpretation of our data should consider the limitations of our experimental approaches.

In the jetlag experiment we found that offspring with a reduced expression of GR in SCN astrocytes re-entrain slower after a shift of the LD cycle. However, we previously showed that offspring prenatally exposed to CORT out-of-phase also produce significantly higher levels of circulating GC compared to the naïve group [10]. In this context, the re-synchronization might depend on the resulting GR–CORT interaction in the SCN. Furthermore, in the in vivo situation, the connection with the PVN and other GR-responsive brain regions could also have an influence [18]. The possibility that the SCN in vivo remains responsive to GCs, integrating this endogenous signal with external light input, could be the reason why in organotypic cultures, carefully dissected from surrounded tissue and exposed to constant culture conditions, GR agonists have failed to shift the expression rhythm of PER2 in adult tissue [12,13].

An effect of GCs directly on the SCN could explain, at least in part, the previously reported effects of this hormones on light entrainment [16,17], locomotor activity [18], and SCN cellular/molecular changes both in vivo and in vitro [51–55,84–86]. SCN astrocytes are considered essential for circadian timekeeping [32–36], controlling extracellular levels of GABA [35,36] and glutamate [32,33], and our study shows that they respond to circulating GCs, and likely contribute to integrating and computing several timing signals for the SCN pacemaker. Further experiments are necessary to functionally link the specific action of GCs on SCN astrocytes and dissect the machanistics of how time cues get integrated in vivo.

Another important limitation to consider is that we cannot directly compare absolute numbers of cells expressing the receptor from the transcriptional and immunohistochemical data. While absolute cell numbers expressing *Gr* are biased by the single-cell sample processing procedure [87], the absolute numbers of cells expressing GR assumes that the whole cell can be visualized in the staining. Although a high proportion of astrocytes express GR in the SCN, it is possible that some SCN neurons, microglia and oligodendrocytes also receive and respond to peripheral GCs. Finally, another limitation of our study lies in the approach used to measure Ca^2+^ events in acute SCN slices which only allowed us to detect events in the soma and main processes. Since we aimed at scanning the largest area possible of the SCN slice to capture the effects of CORT, 3D scanning was not feasible reducing even more the capacity to detect faster events. Genetically encoded Ca^2+^ sensors, although technically challenging to implement in young mice, could provide higher sensitivity and specificity.

Despite these limitations, our findings provide a novel framework for understanding how GCs influence the central pacemaker along development and may have important physiological and therapeutic implications. For example, they could inform strategies to reduce the long-term effects of the antenatal administration of GCs (indicated to 90% of pregnancies at risk of premature delivery) and also restore circadian synchrony in conditions of temporal misalignment, such as in jet lag or shift work.

## Materials and methods

### Ethics statement

All experiments in mice were ethically approved by the Committee on Animal Health and Care of the Government of Schleswig-Holstein, Germany (protocol approval number 4_2017_08_30_Oster and 86-10/22_Oster) and the Ethical Committee of the Basque Country University, Spain (protocol approval number M20_2023_064_Astiz). Experiments were performed according to international guidelines (2010/63/EU) on the ethical use of animals.

### Animals

All mice used in this study were kept in light (L):dark (D) 12:12 conditions in which light intensity was fixed at ca. 300 lux with abrupt LD/DL transitions. Temperature was maintained at 22 ± 2 °C and at a relative humidity of 60 ± 5% with *ad libitum* access to standard chow food and water.

For the prenatal manipulation, C57BL6/J males and females were purchased from Janvier labs, France. Females of 2–4 months of age were group-housed, estrous cycles were synchronized by adding male bedding to the females’ cage. After 4 days (when most of them reach proestrus) females were individually housed overnight (ON) in presence of a fertile and experienced male. The next day was considered GD0.5 if a vaginal plug was present. Females were separated, single-housed and left undisturbed until gestational day (GD) 12.5 when successful pregnancy was confirmed by weight increase. On GD 15.5 and 16.5, mice were subcutaneously injected with corticosterone (CORT, S1 Table) at a concentration of 5 mg/kg body weight (dissolved in polyethyleneglycol-400 (PEG-400) 50% in PBS, S1 Table). These time points during mouse pregnancy were selected to mimic the time window in which human fetuses receive antenatal GCs (i.e., when an elevated risk of premature birth is detected between gestational week 24–34). The dose was selected to override the capacity of the placenta to inactivate maternal CORT. One hour after injection, the levels of CORT in the fetal blood increased to a concentration comparable to the maternal GCs circadian peak [10]. Both days the injections were performed at *zeitgeber* (ZT) 0, when the lights were switched on in the animal facility (i.e.,: 6 a.m., out of phase with the maternal physiological peak) or at ZT12, when the lights were switched off (i.e.,: 6 p.m, in phase with the maternal physiological peak). The naïve group was generated from females that were undisturbed during pregnancy with the only intention of comparing the effect of the manipulation timing to an undisturbed condition. Between postnatal day (PND) 60–80, a jet-lag experiment (i.e.,: 6-hour phase advance in the LD cycle) was performed in CORT exposed and naive offspring to assess the long-term effect of the prenatal intervention in the response of the circadian system. Brain samples of these mice were collected at ZT0 and ZT12 by the end of the behavioral assessment for immunohistochemistry.

For the snRNA-seq experiments and all validation experiments, the same protocol was followed to generate the samples from pregnant mice. After mating, females were separated, single-housed and left undisturbed until the appropriate GD/PND to obtain the samples between ZT3 and 5 (i.e., 3–5 hours after lights were switched on) to prevent an influence of the collection time on the expression of clock or clock-controlled genes.

For the proximity ligation assay and the characterization of GR activation by immunohistochemistry, male and female C57BL/6J mice at PND30 were used. Brains and plasma were collected at ZT0 and ZT12 and at ZT0 one hour after injecting them subcutaneously with either vehicle (PEG-400 50% in PBS) or corticosterone (CORT 5 mg/kg b.w. PEG-400 50% in PBS) at ZT23.

For the calcium imaging experiment, male and female C57BL/6J mice at PND 20–27 were used for the preparation of acute SCN slices between ZT0–2 and between ZT12–14.

### Jet lag experiments

Circadian locomotor activity was assessed using running wheels in LD conditions (300 Lux) with an abrupt LD/DL transition. Mice were housed in individual cages, wheel revolutions were counted every minute (n = 14–16/group). On day 8, the dark phase was advanced for 6 hs while recording the activity. Data collection and analysis were conducted using ClockLab software. The average of the activity onset time before and after the phase shift was calculated to quantify the time at which half of the phase shift was completed (PS_50_). After the behavioral analysis mice were euthanized at ZT0 and ZT12. Brain sections from ZT12 mice were used to assess the expression of GR in SCN astrocytes by immunohistochemistry (see below). In all the groups, both males and females were included (S1 Fig).

### Samples for snRNA-seq and validation

To reduce sex bias and litter effects, SCNs were collected from only 1 male and 1 female fetus/pup per mother of at least 5 different mothers. Then, 10 samples were pooled per developmental stage. Brains were dissected and sliced in 250 μm-thick coronal sections with a vibratome in ice-cold HBSS 1× (S1 Table). GD17.5 and PND2 brains were placed in a block of low-melting agarose 4% (S1 Table) and PND10 and 30 brains were glued directly on the vibratome platform. At least 3 sections were placed on RNase-free glass slides, incubated for 2 min with a nuclei fluorescent marker (DAPI 300 nM, S1 Table) and observed under the microscope. The SCN from the medial section was chosen, dissected with scalpel under the microscope and snap frozen in liquid nitrogen (S3 Fig). For validation experiments (in situ hybridization and immunohistochemistry), brains from fetuses (GD17.5), pups (PND2 and PND10) and adult mice (PND30) from the same litters were collected and immediately frozen in OCT embedding matrix (S1 Table) on dry ice. Fetal sex was determined by PCR as previously described to pool samples from 5 males and 5 females [88]. Briefly, DNA was isolated from the tail by incubating 1 hour at 55 °C with shaking in 20 µL of 50 mM Tris (pH 8), 2 mM NaCl, 10 mM EDTA, 1% SDS containing 0.5 mg/mL of proteinase K. After 1:10 dilution with water, proteinase K was inhibited by incubation for 10 min at 95 °C. One µL of DNA was used in 20 µL of PCR reaction containing 1× ammonium buffer, dNTPs (S1 Table), MgCl_2_, and Taq polymerase (S1 Table). PCR was run as follows 10 min at 94 °C, 33 cycles of (40 s at 94 °C, 60 s at 50 °C, 60 s at 72 °C), and 5 min 72 °C. The presence of IL-3 indicated females (544 bp) and the presence of IL-3 (544 bp) and SRY (402 bp) indicated males. The following primers were used at a concentration of 20 µM IL-3 (5′-GGGACTCCAAGCTTCAAT- 3′ and 5′-TGGAGGAGGAAGAAAAGCAA- 3′) and SRY (5′-TGGGACTGGTGACAATTGTC- 3′ and 5′-GAGTACAGGTGTGCAGCTCT- 3′). In newborns and adults, sex was determined by visually inspecting the ano-genital distance.

### snRNA-seq

Nuclei were isolated from fresh frozen SCN tissue in a total of 6 batches corresponding to the respective developmental timepoints (10 fetuses/pups per developmental stage) in ice cold lysis buffer (10 mM Tris-HCl, pH 7.4, 10 mM NaCl, 3 mM MgCl_2_, 0.1% IGEPAL CA-630 (S1 Table), 1% Recombinant RNase Inhibitor (20 U/µL, S1 Table), 2% BSA). For the two later timepoints (PND10 and PND30) library preparation and sequencing was repeated to increase the number of cells recovered in the first run and to confirm the absence of batch effects (S4 Fig). After 10 min incubation on ice with intermediate mixing by pipette, the suspension was strained through a 20 µm cell strainer, pelleted (4 °C, 500*g*, 5 min), resuspended in ice-cold nuclei suspension buffer (10 mM Tris-HCl, pH7.4, 10 mM NaCl, 3 mM MgCl_2_, 1% Recombinant RNase Inhibitor (20 U/µL, S1 Table), 2% BSA) and quantified in a Neubauer chamber with trypan blue staining followed by snRNA-seq library preparation using Chromium Next GEM Single Cell 3′ Kit v3.1 chemistry (S1 Table) on a 10× Genomics device according to manufacturer’s recommendations. Libraries were quantified, and library fragment size distribution was determined using a Qubit 1× dsDNA HS assay and an Agilent High Sensitivity DNA Kit (S1 Table), respectively. Libraries were multiplexed and sequenced using P2 and P3 (100 Cycles) reagents on a NextSeq 2000 device (Illumina, S1 Table). Sequencing data was demultiplexed and converted to FASTQ format using bcl2fastq2 v2.20 (Illumina) and count matrices were generated with Cellranger v5.0.1 (10× Genomics) using the mouse reference transcriptome mm10-2020-A (10× Genomics). The rest of the analysis was performed using Seurat v4 [89] and standard R packages. Custom filtering at the cell level was performed with a minimum of 750 UMI counts and between 500 and 10,000 distinct features (genes), a maximum of 2.5% mitochondrial and 3% ribosomal reads, as well as a doublet score (scrublet-0.2.3 [90]) below 0.15. Cluster annotation was performed based on differentially expressed marker genes from published mouse anterior hypothalamus development and SCN data (S5a Fig) [27,28,46–48,91,92]. Single-nuclei suspensions resulted in a total of around 8,000 nuclei per snRNA-seq library preparation as determined by nuclei counting. The final sequencing output contained a median of 4,550 transcripts (unique molecular identifiers) and 2,326 distinct genes per nucleus after filtering out poorly sequenced nuclei, nuclei with abnormally high mitochondrial and ribosomal gene counts, and nuclei predicted to be doublets. The final filtered and merged dataset comprised a total of 22,796 high-quality single nuclei across the four stages defining a developmental trajectory. To assess whether the dissection protocol introduced a contamination with extra-SCN cells in our dataset, we implemented a bioinformatic strategy similar to others [45,46]. To classify nuclei as extra-SCN, we focused on marker genes expressed in the preoptic area (rostral to SCN), arcuate nucleus (caudal to SCN) and paraventricular nucleus (dorsal to SCN) at each developmental stage. Since the SCN neurons express particularly high levels of genes from the molecular clock machinery, we further confirmed that cells classified as extra-SCN neurons in our data set express low levels of clock genes (S5b Fig). It is interesting to note that the cluster we annotated as early SCN neurons (mainly present at GD17.5 and PND2, Fig 2c and 2d), showed expression of more than one marker defining different adult SCN neuronal populations (i.e., these are *Vip*^+^, *Avp*^+^, *Nms*^+^, *Cck*^+^, *Grp*^+^). These cells likely share the developmental origin but might not be fully mature yet. In support of this interpretation, the cluster we annotated as early SCN neurons showed low levels of clock gene expression (except for *Rora* and *Rorb*) as it has been described during the perinatal period (S5b Fig) [40,91,93]. The pseudotime trajectory along developmental time in the astroglial subset was manually selected to follow along the astrocyte and not the ependymal developmental trajectory (S6c and S6d Fig). To monitor cellular response to GC stimulus over developmental stages, single-sample Gene Set Enrichment Analysis (ssGSEA) was applied to snRNA-seq data using the escape R-package (https://doi.org/10.18129/B9.bioc.escape). The msigdbr and getGeneSets functions were used to fetch and filter the corresponding Ontology gene sets (C5) of *Mus musculus* from the MSigDB [94,95]. EnrichIt with default parameters, except for using 10,000 groups and variable number of cores, was performed on the seurat-object (S6f Fig). To follow *Gr* expression dynamics during development, trajectory analyses were performed for neuronal (excluding extra-SCN and unidentified neuron clusters) and astroglial subsets across developmental stages using monocle3 [96,97].

### snRNA-seq validation

To validate snRNA-seq data and assess regional distribution we performed in situ hybridization and immunohistochemistry. Each reaction was performed in three sections per mouse and a total of 4–6 different mice per time point of both sexes. Both validations were performed on 12 µm-thick cryosections, fixed with PFA 4% in PBS 1× for 20 min at RT.

In situ hybridization was performed using RNAscope Multiplex Fluorescent Assay (S1 Table) on brain tissue sections of mice at the same developmental stages (GD17.5, PND2, PND10 and PND30). The targets, negative and positive controls probes (representative pictures in S8a and S8b Fig) were hybridized following manufacturer’s instructions. This method allowed us to visualize three different RNAs simultaneously to validate the identity of the main cell clusters identified in the SCN throughout development. We used *Pdgfra*-C1 to label oligodendrocytes and NG2 cells, *Syt1*-C2 to label neurons and *Aldh1L1-*C3 to label astrocytes and ependymal cells (Fig 2e). To validate the presence of *Gr* we used *Gr*-C1 to label the receptor, *Syt1*-C2 to label neurons and *Aldh1L1-*C3 to label astrocytes (Fig 3d) in sections of PND30 mice. Slices were counterstained with a DAPI solution provided with the kit and mounted with Prolong Gold mounting media (S1 Table).

*Immunohistochemistry* was performed after blocking for 1 hour at RT with normal goat serum 4% and Triton X-100,0.4% followed by incubation with rabbit anti-GR (1:200) (S1 Table) for 2 or 1 days at 4 °C, respectively. In parallel, the negative control was incubated with blocking solution (representative picture in S8c Fig). After 5 washes of 5 min with TBS 1×, we incubated with mouse anti-GFAP (1:200), chicken anti-Vimentin (1:500), or mouse anti-Vgat (1:200) (S1 Table) or blocking solution in case of the negative control ON at 4 °C. Next day, after 5 washes of 5 min with TBS 1×, all slices were incubated with a mix of the three secondary antibodies (all in 1:500 dilution), a-rabbit Alexa 555, a-mouse Alexa 488 and a-chicken FITC (S1 Table) in TBS 1× solution for 2 hours at RT in a dark chamber. Slices were washed, counterstained with DAPI (300 nM in PBS) for 5 min and mounted with Prolong Gold media (S1 Table). Quantification of the percentage of cells expressing GR within the astrocytes (GFAP/VIM+) and GR within neurons (VGAT+) was assessed manually by an experimenter who was blinded to the identity of the pictures using the counting tool of ImageJ software (NIH, Bethesda, Maryland, USA). In 40× zoomed pictures, total nuclei were counted in the DAPI picture and total GR+ cells in the green channel. In the red and green merged image, the total GR+ cells that were also GFAP/VIM+ or VGAT+ were counted. For each time point, pictures from 4 to 6 mice were analyzed.

### Confocal imaging

Images at different magnifications were acquired using a Leica Stellaris5 confocal microscope (Leica Microsystems CMS GmbH, Germany). A HC PL APO 20×/0.75 Air CS2 and PL APO 40×/1.30 Oil CS2 objectives were used, with the latter also being used for images taken with optical zoom. The microscope features an AOBS device for fluorescence emission detection allowing free tuning and high-speed switching. The LAS X software, version 4.5.0.25531 as well as the open version (3.3.0.16799-Leica Microsystems CMS GmbH, Germany) were used for the analysis of the images. ImageJ software (NIH, Bethesda, Maryland, USA) was used to create the Z-stack and the merge images. Raw images and metadata are available upon request.

### Corticosterone in plasma

Corticosterone was measured in plasma by ELISA (S1 Table) according to the manufacturer’s instructions. Samples were collected in EDTA-coated tubes and plasma isolated by centrifugation at 240*g*, 20 min, 4 °C. Corticosterone concentration was assessed in duplicates from mouse plasma obtained at ZT0 and ZT12 and at ZT0 one hour after injection with VEH or CORT.

### Proximity ligation assay (PLA)

PLA was performed and quantified as previously described [49]. Five µm paraffin-embedded coronal sections containing the SCN and the PVN as a positive control (S9a and S9c Fig) were gradually rehydrated through incubation in graded alcohols and antigen retrieved in citrate buffer pH 6 in a 700W microwave. Alternatively, 12 µm fresh frozen sections were fixed in 4% PFA for 20 min at RT, and antigen retrieved in citrate buffer pH 6 in a 700W microwave. Thereafter, samples were blocked in PLA blocking buffer (S1 Table) for 1 hour at 37 °C, and subsequently incubated overnight with the primary antibodies anti-GR, anti-HSP90, and anti-GFAP (S1 Table) at 4 °C in Tris buffer containing 0.05% Tween-20 (TBST). The next day the samples were washed and incubated with secondary conjugates (S1 Table) ON at 4 °C. Samples were then washed and incubated with ligation solution (S1 Table) for 2 hours at 37 °C and washed again in TBST. The negative controls were run omitting either ligase, PLUS secondary probe, or MINUS secondary probe as appropriate (representative picture in S9b Fig). Finally, sections were incubated at 37 °C in polymerization solution (S1 Table) for 4 hours and then washed with TBS. Samples were counterstained by incubation with DAPI in TBS and secondary antibodies (S1 Table) for 30 min at RT. Sections were mounted with Fluorsave (S1 Table) and stored in the dark at 4 °C until imaging on a Leica Stellaris5 confocal microscope (Leica Microsystems CMS GmbH, Germany). Images were quantified automatically with ImageJ, PLA channel images were background-subtracted and thresholded. Puncta within the GFAP+ and GFAP− masked area of the SCN that were between 3 and 25 pixels in size were automatically counted as positive and averaged per animal and time point. We assessed *n* = 11–12 mice at each time point in control conditions and *n* = 5 mice in VEH or CORT injected conditions.

### Immunohistochemistry to assess GR activation by circulating GCs

Five µm paraffin-embedded coronal sections containing the SCN were gradually rehydrated through incubation in graded alcohols and antigen retrieved in citrate buffer pH 6 in a 700W microwave. Slides were blocked for 1 hour at RT with normal goat serum 4% and Triton X-100,0.4% followed by incubation with rabbit anti-GR (1:200) and mouse anti-GFAP (1:200) (S1 Table) for 2 or 1 days at 4 °C, respectively. In parallel, the negative control was incubated with blocking solution (representative picture in S8c Fig). After 5 washes of 5 min with TBS 1× all slices were incubated with a mix of the two secondary antibodies (in 1:500 dilution), a-rabbit Alexa 555 and a-mouse Alexa 488 (S1 Table) in TBS 1× solution for 2 hours at RT in a dark chamber. Slices were washed (5 times, 5 min) and incubated with normal rabbit serum 5% (S1 Table) and Triton X-100,0.4% for 1 hour at RT in a dark chamber and followed by incubation with rabbit anti-SOX9 conjugated with Alexa 647 (1:100) (S1 Table) for 2 days at 4 °C. After 5 washes of 5 min with TBS 1× slices were counterstained with DAPI (300 nM in PBS) for 5 min and mounted with Prolong Gold media (S1 Table). Quantification of the percentage of GR+ nuclei (Fig 5a and 5c) and GR+ nuclei co-stained either with GFAP, with SOX9 or with both (S9f Fig) was assessed by an experimenter who was blinded to the identity of the pictures using the counting tool of ImageJ software (NIH, Bethesda, Maryland, USA). In 40× zoomed pictures, total nuclei were counted in the DAPI picture and total GR+ cells in the green channel. In the red and green merged image, the total GR+ cells that were also GFAP+ or in the red and gray merged image total GR+ cells that were also SOX9+ were counted. For each time point, pictures from 6 mice were analyzed.

### *In silico* validation of GR binding and activation in SCN astrocytes

Published data (Morris and colleagues, 2021 [45]) obtained from PND10 to 12 mouse SCN organotypic explants was used to compare gene expression profiles under conditions of expected high (CT15.5) and expected low (CT7.5) GCs. We also used another data set (Wen and colleagues, 2020 [28]) generated from PND56 mouse SCN obtained at different circadian timepoints, and we analyzed CT26 and CT50 (when circulating GCs are low) and CT14 and CT38 (when circulating GCs are high) [28]. Fastq files were downloaded and processed in the same fashion as described in Materials and methods, including cluster annotation by published identity markers. The dataset was filtered to only contain neurons (SCN and extra-SCN) and glial cell clusters and ssGSEA was performed as described above. Single-sided Wilcoxon test (*rstatix::wilcox_test*) with default parameters was applied to statistically compare scores between cells with high and low expected/circulating GCs (S10a–S10e Fig). Additionally, differential ligand receptor analysis was performed using the *Liana+* v1.0.4 package in *python* 3.11.7 [98]. Briefly, differentially expressed genes were identified between samples collected at CTs when GC were expected to be high and CTs when GC were expected to be low in a pseudo-bulk manner per cell type using *get_pseudobulk* function in the python version of *decoupler* v1.5.0, followed by *DeseqStats* function in *pydeseq2* v0.4.4. Ligand-receptor pairs containing these differentially expressed genes were identified using the *multi.df_to_lr* function, with “mouseconsensus” as the ligand-receptor database. These interactions were further filtered to only keep the ones where either the ligand or the receptor was a regulatory target of GR and the expression changed in the direction expected based on the mode of regulation (i.e., activation or repression). The mouse regulon data for GR was obtained using the CollecTRI database [98] using the *decoupleR::get_collectri* function with *split complexes* parameter set to *false* (S10c Fig).

### Two-photon calcium imaging

Since the time-of-day can affect the magnitude of the calcium signals Ca^2+^, we prepared all the acute slices from animals euthanized during the early light phase (ZT0–2) or the early dark phase (ZT12–14). Brains were rapidly removed from the skull and coronal sections (250 µm thick) containing the SCN were prepared in a Leica VT1200S vibratome. Slicing was performed in ice-cold low-Na^+^ low-Ca^2+^ high Mg^2+^ artificial cerebrospinal fluid (aCSF) solution gassed with carbogen (95% O_2_/5% CO_2_) and containing (in mM): 189 sucrose, 3 KCl, 1.25 KH_2_PO_4_, 5 MgSO_4_·7H_2_O, 26 NaHCO_3_, 10 glucose and 0.1 CaCl_2_·2H_2_O (pH 7.3–7.4). To interrupt the connection between the SCN and the PVN, a cut between both regions perpendicular to the third ventricle was performed with a scalpel under the magnifying glass (Fig 6d and 6e). Slices were then transferred to recovery aCSF containing (in mM): 124 NaCl, 2.7 KCl, 1.25 KH_2_PO_4_, 2 MgSO_4_·7H_2_O, 26 NaHCO_3_, 10 glucose, 2 CaCl_2_·2H_2_O and 0.4 ascorbic acid (pH 7.3–7.4) gassed with carbogen and containing the cell-permeant calcium fluorescent dye Fluo-4-AM (S1 Table) at 2 μM in 0.01% pluronic (S1 Table). Fluo-4 staining was performed in the dark for 1 hour at RT. Slices were transferred to oxygenated recovery aCSF and allowed to wash for at least 1 hour. For those experiments assessing CORT effects independent of transcriptional or post-transcriptional events, we supplemented the aCSF with actinomycin D 5 μM (S1 Table) and cycloheximide 10 μM (S1 Table). Slices were then transferred to an ultra-quiet imaging chamber (RC-27D, Warner Instruments), perfused at 0.5 mL/min with oxygenated aCSF at 37 °C for imaging in a Femto-2D two-photon microscope (Femtonics) equipped with a Ti:Sapphire MaiTai DeepSee laser (Spectra Physics) tuned at 820 nm. To locate the SCN and check the overall quality of the slice, slices were first imaged in brightfield with infrared illumination and a standard camera. Only one Fluo-4-stained SCN was imaged per animal with 2P-excitation between 30 and 60 µm from the surface as single plane (1 μm). The number of slices per experiment are indicated in the figure legend. Images were acquired with the MES software (Femtonics) at a speed of 1 Hz, 480 × 480 pixels (444 nm/pixel), a 520/60 filter and a water immersion 20× objective (NA 1.0, Olympus). Pharmacological treatments were perfused to the recording chamber at the following working concentrations (in aCSF): 1 μM CORT and CORT-BSA (S1 Table), 1 μM a glucocorticoid receptor antagonist (RU486, S1 Table), 400 μM GAP26 (S1 Table), a Connexin 43 mimetic gap junction inhibitor. Baseline (aCSF) and vehicle (aCSF + 0.1% DMSO) recordings were performed before the sequence of pharmacological treatments. Each recording lasted for 5 min, the Z-plane was maintained between recordings (Fig 6a and 6b).

### Calcium imaging processing and analysis

Images were cropped in FIJI [99], drift corrected with EZCalcium in MATLAB [100] and denoised with the deep learning-based software SUPPORT [101]. Active regions of interest (ROIs) were either computed in Aqua2 [102], or outlined using the dF/F0 images computed in ImageJ software. The resulting ROIs were analyzed with the program Calsee in MATLAB [103] to quantify calcium transients. Event detection was determined when the fluorescence signal (dF/F0) was at least three times the standard deviation of the baseline, calculated with a moving threshold of 10 frames. To assess calcium transients from astrocytes we used a step-by-step criterion: In a first step, we detected Fluo-4-loaded astrocyte-like ROIs (i.e., small soma with complex radial arborizations) based on their morphology (S11b Fig). In a second step, we filtered those with Ca^2+^ events longer than 10 s (Figs 6c and S11c). In a third step, we further filter for those events with signal decay longer than the signal rise (Fig 6c), a feature of astrocytic events in the soma and the main processes. All events with less than 0.1 dF/F0 were considered false positives, except in those experiments assessing CORT effects independent of transcriptional or post-transcriptional where the amplitude threshold was 0.05. These filtered ROIs outlined astrocytes with visible events, and thus represented the number of active astrocytes within the region during the recording period. In addition, we also computed for the area under the curve as an integrated metric of the response of each treatment. To analyze the presence of synchronic Ca^2+^ events, we created binary raster tables of individual astrocyte Ca^2+^ events in MATLAB. To this aim, we used an expanding temporal window: an initial 3-s window identified timepoints where ≥2 astrocytes exhibited Ca^2+^ events; when additional active astrocytes were detected in a subsequent 3 s interval, the window expanded iteratively until no new participants were found. The “level” of the synchronic event (number of ROIs in sync) was defined as the total number of co-active ROIs within the expanded window and was assigned to the time point of the first detected event. This approach captures the full temporal extent and participation level of coordinated astrocytic Ca^2+^ episodes while preventing fragmentation of sustained synchronized events into multiple lower-level detections.

### Statistics

Immunohistochemistry and in situ hybridization to validate snRNA-seq data were performed on three SCN sections per mouse and at least three different mice per time point of both sexes; only representative pictures are included. To assess statistical differences between two groups we used two-tailed *T* test and between three or more groups, we used one-way ANOVA followed Holm–Sidak multi-comparison test, after confirming normality by Shapiro–Wilk test. To assess statistical differences between three or more groups and two variables, we used two-way ANOVA followed Holm–Sidak multi-comparison test, after confirming normality by Shapiro–Wilk test. If normality was not confirmed, we used the corresponding non-parametric test. A negative binomial generalized linear model was used to assess statistical differences between treatments in Ca^2+^ events/min, this analysis accounts for ROIs nested within slices (animals) avoiding pseudoreplication. To assess synchronized Ca^2+^ events we used a Poisson GLMM with experiment (slice) as a random intercept, accounting for the repeated-measures design in which the three treatment conditions were applied sequentially to the same slice. To assess whether changes in synchronized events were independent of changes in total event frequency, we fitted an additional Poisson GLMM with log(total events) as an offset and experiment as a random intercept, effectively modeling the rate of synchronized events per total event across conditions. In all cases, the tests used and the results of the test as well as the size of the experimental groups are indicated in the figure captions. *P* values <0.05 were considered statistically significant. FDR adjusted *p* values <0.05 were considered statistically significant.

## Supporting information

S1 Fig Mice prenatally exposed to CORT out-of-phase re-adapt slower to a shift in the LD cycle. On the left: Double-plotted running wheel actograms before and after jet lag of all mice included in Fig 1, dark phase is shaded in gray and sex of the mice is indicated. Naïve offspring (*n* = 14, 8 males and 6 females), out-of-phase offspring (*n* = 15, 7 males and 8 females) and in-phase group (*n* = 16, 8 males and 8 females). **a,b)** Circadian pattern of locomotor activity in LD 12:12 before the shift of the LD cycle (a) and a zoomed-in on the onset of activity in the LD transition (b). In both cases, the running-wheel activity of 10 days prior to jetlag was averaged for each individual animal and then for each group. **c)** Onset of activity in the LD transition the day prior to jetlag averaged by each group. Data are expressed as mean ± SEM and analyzed by 2-way ANOVA, no significant effect of the interaction between the prenatal treatment group and the ZT was found. **d)** Average of the PS_50_ in all three groups separated by sex. Data are expressed as mean ± SEM. Data was analyzed by 2-way ANOVA with sex and prenatal treatment as factors. The statistical analysis showed no interaction of both variables (*F*(2,39) = 0.0081, *p* = 0.9919), no significant effect of sex (*F*(1,39) = 0.039, *p* = 0.842) and a significant effect of the treatment (*F*(2,39) = 5.017, *p* = 0.0115). **e)** Quantification of GR expression in the whole SCN. Data are expressed as mean ± SEM (*n* = 8–12/group), passed normality test and statistical difference was assessed by one-way ANOVA, *F*(2,27) = 2.447, *p* = 0.1055. Numerical data can be found in S1 Data file.
(TIFF)

S2 Fig Activity before and after injection/manipulation at ZT0 and ZT12 compared to naïve mice. **a)** Averaged locomotor activity bouts over 4 days from 7 female mice kept in LD 12:12 and left undisturbed (naïve), injected with VEH (PEG-400 50% in PBS) or injected with CORT (5 mg/Kg b.w in PEG-400 50% in PBS) at ZT0. The arrow indicates the injection time with VEH or CORT and the dotted area depicts the injection day, zoomed at the bottom. **b)** Averaged locomotor activity bouts over 4 days from 7 female mice kept in LD 12:12 and left undisturbed (naïve), injected with VEH (PEG-400 50% in PBS) or injected with CORT (5 mg/Kg b.w in PEG-400 50% in PBS) at ZT12. The arrow indicates the injection time with VEH or CORT and the dotted area depicts the injection day, zoomed at the bottom. **c)** Average of activity bouts the first 6 hours after the injection with VEH or CORT at ZT0 or at ZT12 in comparison to a naïve group. Data are expressed as mean ± SEM, normality was confirmed by Shapiro-Wilk test and analyzed by 2-way ANOVA using treatment (None, VEH or CORT) and time of day (ZT0 and ZT12) as factors. As expected, significant differences were found for the time of day *F*(1,36)=145.1, *p* < 0.0001 but the treatments did not change significantly the activity at any of the time points. The activity did not show any difference days after the manipulation. Numerical data can be found in S1 Data file.
(TIF)

S3 Fig SCN dissection strategy. **a)** Brains were dissected and sliced in 250-μm thick coronal sections with a vibratome in ice cold HBSS 1×. To prepare slices from GD17.5 and PND2 fetus/pups, the brains were placed in a block of low melting agarose 4%, while PND10 and 30 brains were glued directly on the vibratome platform. At least 3 sections were placed on RNase-free glass slides, incubated 2 mins with a nuclei fluorescent marker (DAPI 300 nM) and observed under the microscope. The SCN from the medial section was chosen, dissected with a scalpel and frozen in liquid nitrogen. *Created in BioRender. Administrator, S. (2026)* *https://BioRender.com/0y41lu2*. **b)** Examples of sections dissected from all developmental timepoints, scale bars 200 μm.
(TIFF)

S4 Fig Embeddings of merged snRNA-seq data reflect developmental changes and no technical batch effect. **a)** UMAP embeddings of two independent replicates of PND10 and PND30 merged without batch-correction, where cells are colored by individual experiment and developmental stage. **b)** Same UMAP embedding colored by developmental stage. Replicates are clustering together and within clusters timepoints are distinctly separated. Raw transcriptomic data can be found under GEO accession number GSE240803.
(TIFF)

S5 Fig Single-nuclei transcriptional profiling of the SCN along maturation. **a)** Cell type composition in the dataset. **b)** Violin plot showing the total expression of clock genes across the main clusters. Raw transcriptomic data can be found under GEO accession number GSE240803.
(TIFF)

S6 Fig Changes in *Gr* expression along SCN maturation. **a,b)** Density plot of the *Gr* expression in oligodendorcytes and microglia segregated by developmental stage. The percentage (absolute number) of cells with non-zero expression of *Gr* are indicated for each cell cluster. **c)** *Gr* expression in astrocytes and ependymal cells subset is represented along the developmental pseudotime trajectory (trajectory in the direction of ependymal cells was manually excluded from the analysis). **d)** *Gr* expression in the neuronal subset (excluding extra-SCN neurons and unidentified neuronal cluster) is represented along the developmental pseudotime trajectory. In c and d, colors represent developmental timepoints, except for cells with zero expression represented in gray, the solid line represents spline fit to the datapoints. **e)** Percentage of *Gr*^+^ cells co-expressing astrocytic markers (*Gfap/Vim*^+^ in red) or neuronal marker (*Slc32a1 (Vgat)*^*+*^ in cyan) are plotted for each developmental age, linear fit curves show the tendency of the developmental change. **f)** Median enrichment scores for the geneset segregated by the time points and cell types. Only astroglial cells (astrocytes, immature astrocytes/ependymal cells) and neurons (except extra-SCN neurons and unidentified neuronal cluster) are compared. EnrichIt with default parameters, except for using 10,000 groups and variable number of cores, was performed on the seurat-object. FDR adjusted p values <0.05 were considered statistically significant. Raw transcriptomic data can be found under GEO accession number GSE240803.
(TIFF)

S7 Fig Cell-specific changes in GR expression along SCN maturation. **a)** Representative immunohistochemistry confocal images of the SCN (DAPI), for GR (green) in astrocytes (GFAP/VIM^+^; red) for GD17.5, PND2 and PND10. The dotted white regions demarcate the SCN and the yellow squares the magnified areas. Scalebars correspond to 200 µm, except for the zoomed images, where it is 20 µm. Arrows highlight co-localization (GR and GFAP+VIM). **b)** Representative immunohistochemistry confocal images of the SCN (DAPI), for GR (green) in neurons (VGAT^+^; red) for GD17.5, PND2 and PND10. The dotted white regions demarcate the SCN and the yellow squares the magnified areas. Scalebars correspond to 200 µm, except for the zoomed images, where it is 20 µm. Arrows highlight co-localization (GR and VGAT). Staining was performed on at least three SCN sections per mouse and a total of three different mice per time point of both sexes. OC: optic chiasm, 3V: third ventricle.
(TIFF)

S8 Fig In situ validation controls. **a)** Representative confocal images showing the results of the RNAscope negative control performed following manufacturer’s instructions (negative probe followed by the three amplification steps and the three HRP-Channels/TSA fluorophore (TSA vivid 520, 570, and 650)/HRP-Blocker and counterstained with DAPI. **b)** Representative confocal images showing the results of the RNAscope positive control performed following manufacturer’s instructions (mixture of positive probes one/channel: Polr2a-C1, Ppib-C2 and Ubc-C3) followed by the three amplification steps and the three HRP-Channels/TSA fluorophore (TSA vivid 520, 570 and 650)/HRP-Blocker and counterstained with DAPI. **c)** Representative confocal images of the immunohistochemistry negative controls run without primary antibodies and incubation for 2 hours at RT in a dark chamber with the mixture of all three secondary antibodies (anti-mouse Alexa 488, anti-chicken FITC, and anti-rabbit Alexa 555). Scalebar correspond to 100 µm for all pictures.
(TIFF)

S9 Fig GR activation by circulating GCs (controls). **a)** Proximity ligation assay in the paraventricular nuclei (PVN) as a positive control. Top: reconstruction of the PVN from pictures taken at 40× magnification (scale bar 100 µm). Bottom: zoom in (scale bar 8 µm). The dotted white regions demarcate the PVN. **b)** The negative PLA controls were run omitting either ligase, MINUS secondary probe or PLUS secondary probe as appropriate, no signal was observed. **c)** Quantification of the number of GR-HSP90 signal (puncta)/GFAP+area in the PVN at both timepoints (*n* = 3–5 mice/time point). Statistical difference was assessed by two-tailed Mann–Whitney, *p = 0.0357. **d)** Quantification of the number of GR-HSP90 signal (puncta)/GFAP− area in the SCN at both timepoints ZT0 (low GCs) and ZT12 (high GCs). Data passed normality test and statistical difference between ZT0 and ZT12 was assessed by two-tailed *T* test, *t* = 2.443, df = 16, *p = 0.0265. **e)** Quantification of the number of GR-HSP90 signal (puncta)/GFAP− area in the SCN one hour after subcutaneously injecting either VEH or CORT (5 mg/Kg body weight at ZT23). Data passed normality test and statistical difference between ZT0 VEH and ZT0 CORT was also assessed by two-tailed *T* test, *t* = 4.043, df = 8, **p = 0.0037. **f)** The percentage of GR^+^ nuclei co-stained either with GFAP, with SOX9 or with both astrocytic makers in samples from Fig 5. **g)** Expression of *Aldh1l1*, *Gfap*, *Sox9*, and *Sparcl1* in the snRNA-seq dataset plotted in the same UMAP embedding as Fig 3a across different developmental stages (including only SCN neurons and astrocytes). Bottom: table showing the percentage of cells expressing each of the markers at PND30. Numerical data can be found in S1 Data file.
(TIFF)

S10 Fig Reanalysis of Morris and colleagues (2021) and Wen and colleagues (2020) single-cell RNA-seq dataset. **a)** Data from Morris and colleagues (2021) was downloaded from a public repository and processed in the same fashion as our own snRNA-seq dataset, before filtering for only glial and neuronal cell clusters. Dot plot showing expression of the same markers used in Fig 2c (except epithelial markers) in Morris and colleagues dataset showing the presence of a similar set of cell types/clusters. **b)** Median enrichment scores for the geneset segregated by cell types and expected GC levels. Single-sided Wilcoxon test was applied to statistically compare scores within cell types between cells expecting high and low GCs. Corresponding *p*-values are displayed above the heatmap. **c)** Predicted ligand-receptor interactions between the three cell types where the gene in bold is a regulatory target of *Gr* and is differentially expressed in the expected high GC condition based on activation/repression relationship. Asterisk indicates adjusted *p*-value <0.05. **d)** Data from Wen and colleagues (2020) was downloaded from a public repository and processed in the same fashion as our own snRNA-seq dataset, before filtering for only glial and neuronal cell clusters. Dot plot showing expression of the same markers used in Fig 2c (except epithelial markers) in Wen and colleagues dataset showing the presence of a similar set of cell types/clusters. **e)** Median enrichment scores for the geneset segregated by cell types and circulating GC levels. Single-sided Wilcoxon test was applied to statistically compare scores within cell types between cells with high and low GCs. Corresponding *p*-values are displayed above the heatmap. Raw transcriptomic data can be found under GEO accession number GSE240803.
(TIFF)

S11 Fig SCN astrocytes respond to GCs inducing Ca^2+^ waves. **a)** Representative immunohistochemistry confocal images of the SCN (DAPI), for Connexin 43 (Cn43 in red) and astrocytes (GFAP in green). The dotted white regions demarcate the SCN and the magnified area is indicated with a white square. Scale bar correspond to 100 µm. OC: optic chiasm, 3V: third ventricle. **b)** Astrocytes are identified based on their morphology. **c)** Further filtering was done at the analysis pipeline (i.e., cells with events shorter than 10 s are not considered). Representative overlapped astrocyte and non-astrocyte trace to better visualize the differential dynamics of the events included in the analysis. **d)** Decay (time from maximum to baseline)/rise (time from baseline to maximum) ratio of calcium events in astrocytes and non-astrocytes from the experiment in Fig 6g and 6h (*n* = 4 independent slices). Statistical difference was assessed by Mann–Whitney test, *p < 0.05. **e)** Duration of Ca^2+^ events from the experiment in Fig 6g and 6h. The dotted line indicates the duration threshold used to count astrocytic events (10 s). Statistical difference was assessed by Mann–Whitney test, ***p < 0.001 (*n* = 4 independent slices). **f)** Total number of active regions of interest (ROIs) in the SCN upon CORT treatment and when CORT binding to the GR is antagonized by co-incubation with RU486 1 μM (RU), in slices where the PVN–SCN connection was intact or cut (*n* = 3 independent slices per condition). **g)** Duration of Ca^2+^ events, the dotted line indicates the duration threshold used to count astrocytic events (10 s). **h)** area under the curve (AUC) of the amplitude of the fluorescence signal (dF/F0). Statistical differences were assessed by Kruskal–Walli’s test, ***p < 0.001 (*n* = 3 independent slices per condition). **i,j)** Mean amplitude and duration of astrocytic Ca^2+^ events from the experiment in Fig 6g and 6h (*n* = 4 independent slices). **k)** Number of astrocytic Ca^2+^ events comparison between baseline recording (Ctrl) and vehicle (aCSF containing 0.1% DMSO) is represented as independent experiments (*n* = 11 mice, in gray) and as mean (in black). No significant differences were detected. **l–n)** Integrated Ca^2+^ response represented as the area under the curve (AUC) (independent experiments, *n* = 3–4 mice, in gray) and as mean (in black) of experiments shown in Fig 6i–6k. Data are expressed as mean ± SEM, passed normality test and statistical difference was assessed by repreated measures ANOVA *F*(2,6) = 4.48, *p* = 0.064, VEH vs. CORT, *p* = 0.073 (for l) and *F*(2,6) = 1.78, *p* = 0.246 (for m). For the data presented in n, normality was not tested because n was too small, Friedman test was applied, *p* = 0.0556. Numerical data can be found in S1 Data file.
(TIF)

S1 Video Representative movie of an astrocytic Ca^2+^ event, lasting more than 10 s, with a longer signal decay than rise and typical morphology. Fluo-4-AM was used as calcium sensor, the fluorescence emission was measured as a readout of intracellular calcium rise using a two-photon microscope.
(AVI)

S2 Video Representative movie of astrocytic Ca^2+^ events in a followed during the 200 s of recording while applying CORT and CORT + GAP26. Astrocytic calcium transients during synchronized events are depicted with red arrows.
(AVI)

S1 Table Contains detailed information (name, catalogue number, company) of all reagents used in our manuscript. (DOCX)

S1 Data Contains numerical data from main and supplementary figures. (XLSX)

## Abbreviations

aCSF: artificial cerebrospinal fluid
ACTH: adrenocorticotropic hormone
AUC: area under the curve
CRH: corticotropin-releasing hormone
CT: circadian time
GCs: glucocorticoids
GD: gestational day
GLMM: generalized linear mixed-effects model
GR: GC receptor
HPA: hypothalamus–pituitary–adrenal
LD: light–dark
OPCs: oligodendrocyte precursor cells
PLA: proximity ligation assay
PND: postnatal day
PVN: paraventricular nucleus of the hypothalamus
ROIs: regions of interest
SCN: suprachiasmatic nuclei
snRNA-seq: single-nuclei RNA sequencing
VIP: vasoactive intestinal polypeptide
