# Supplementary figures and images for: A developmental shift in glucocorticoid receptor expression preserves glucocorticoid sensitivity in the adult suprachiasmatic nucleus
Source: PLoS Biol. 2026 Jul 7;24(7):e3003870. doi: 10.1371/journal.pbio.3003870 (PMC13340777; doi:10.1371/journal.pbio.3003870)

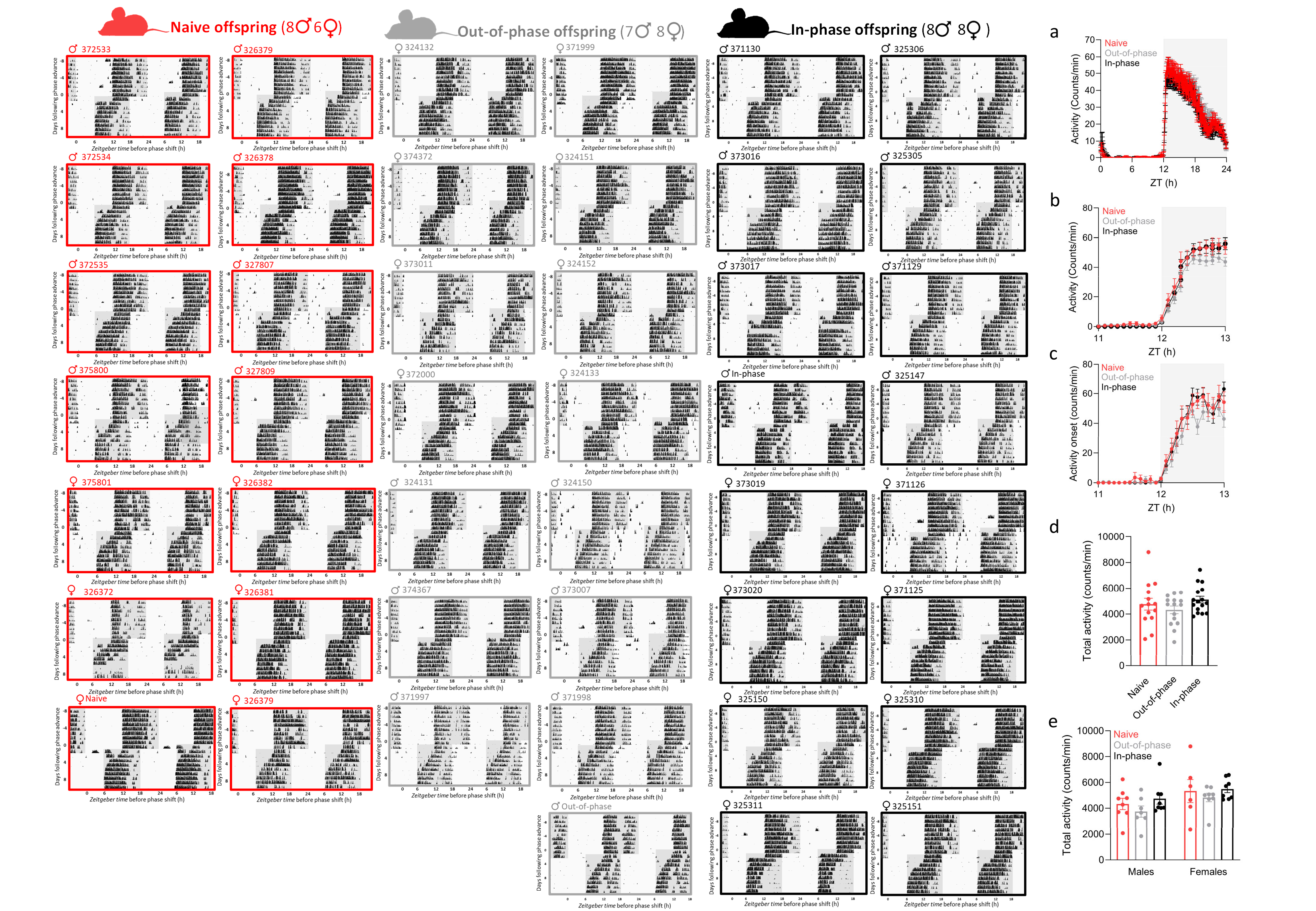

Supplement: S1 Fig — On the left: Double-plotted running wheel actograms before and after jet lag of all mice included in Fig 1, dark phase is shaded in gray and sex of the mice is indicated. Naïve offspring (n = 14, 8 males and 6 females), out-of-phase offspring (n = 15, 7 males and 8 females) and in-phase group (n = 16, 8 males and 8 females). a,b) Circadian pattern of locomotor activity in LD 12:12 before the shift of the LD cycle (a) and a zoomed-in on the onset of activity in the LD transition (b). In both cases, the running-wheel activity of 10 days prior to jetlag was averaged for each individual animal and then for each group. c) Onset of activity in the LD transition the day prior to jetlag averaged by each group. Data are expressed as mean ± SEM and analyzed by 2-way ANOVA, no significant effect of the interaction between the prenatal treatment group and the ZT was found. d) Average of the PS50 in all three groups separated by sex. Data are expressed as mean ± SEM. Data was analyzed by 2-way ANOVA with sex and prenatal treatment as factors. The statistical analysis showed no interaction of both variables (F(2,39) = 0.0081, p = 0.9919), no significant effect of sex (F(1,39) = 0.039, p = 0.842) and a significant effect of the treatment (F(2,39) = 5.017, p = 0.0115). e) Quantification of GR expression in the whole SCN. Data are expressed as mean ± SEM (n = 8–12/group), passed normality test and statistical difference was assessed by one-way ANOVA, F(2,27) = 2.447, p = 0.1055. Numerical data can be found in S1 Data file. (TIFF) [file pbio.3003870.s001.tiff]

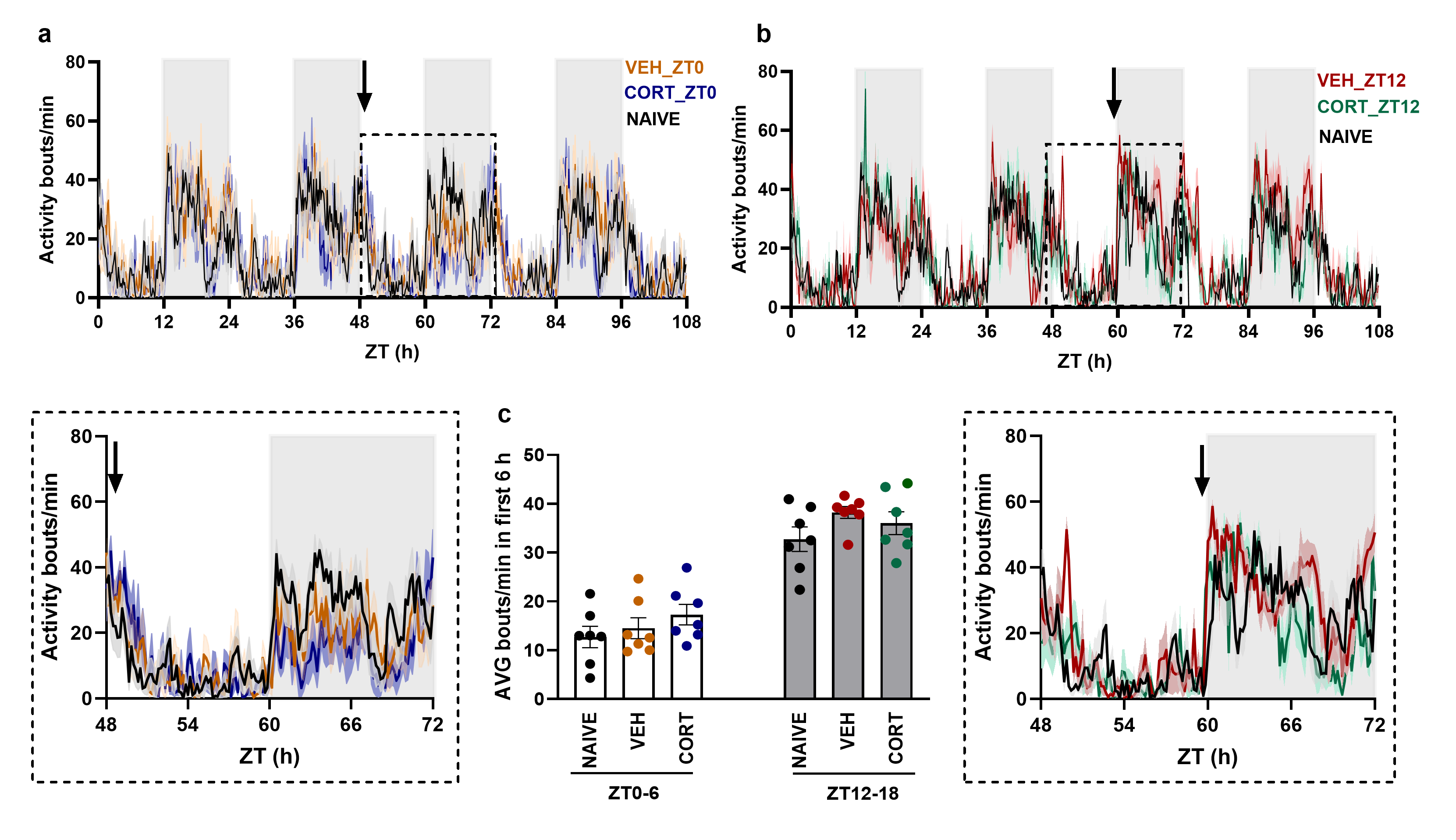

Supplement: S2 Fig — a) Averaged locomotor activity bouts over 4 days from 7 female mice kept in LD 12:12 and left undisturbed (naïve), injected with VEH (PEG-400 50% in PBS) or injected with CORT (5 mg/Kg b.w in PEG-400 50% in PBS) at ZT0. The arrow indicates the injection time with VEH or CORT and the dotted area depicts the injection day, zoomed at the bottom. b) Averaged locomotor activity bouts over 4 days from 7 female mice kept in LD 12:12 and left undisturbed (naïve), injected with VEH (PEG-400 50% in PBS) or injected with CORT (5 mg/Kg b.w in PEG-400 50% in PBS) at ZT12. The arrow indicates the injection time with VEH or CORT and the dotted area depicts the injection day, zoomed at the bottom. c) Average of activity bouts the first 6 hours after the injection with VEH or CORT at ZT0 or at ZT12 in comparison to a naïve group. Data are expressed as mean ± SEM, normality was confirmed by Shapiro-Wilk test and analyzed by 2-way ANOVA using treatment (None, VEH or CORT) and time of day (ZT0 and ZT12) as factors. As expected, significant differences were found for the time of day F(1,36)=145.1, p < 0.0001 but the treatments did not change significantly the activity at any of the time points. The activity did not show any difference days after the manipulation. Numerical data can be found in S1 Data file. (TIF) [file pbio.3003870.s002.tif]

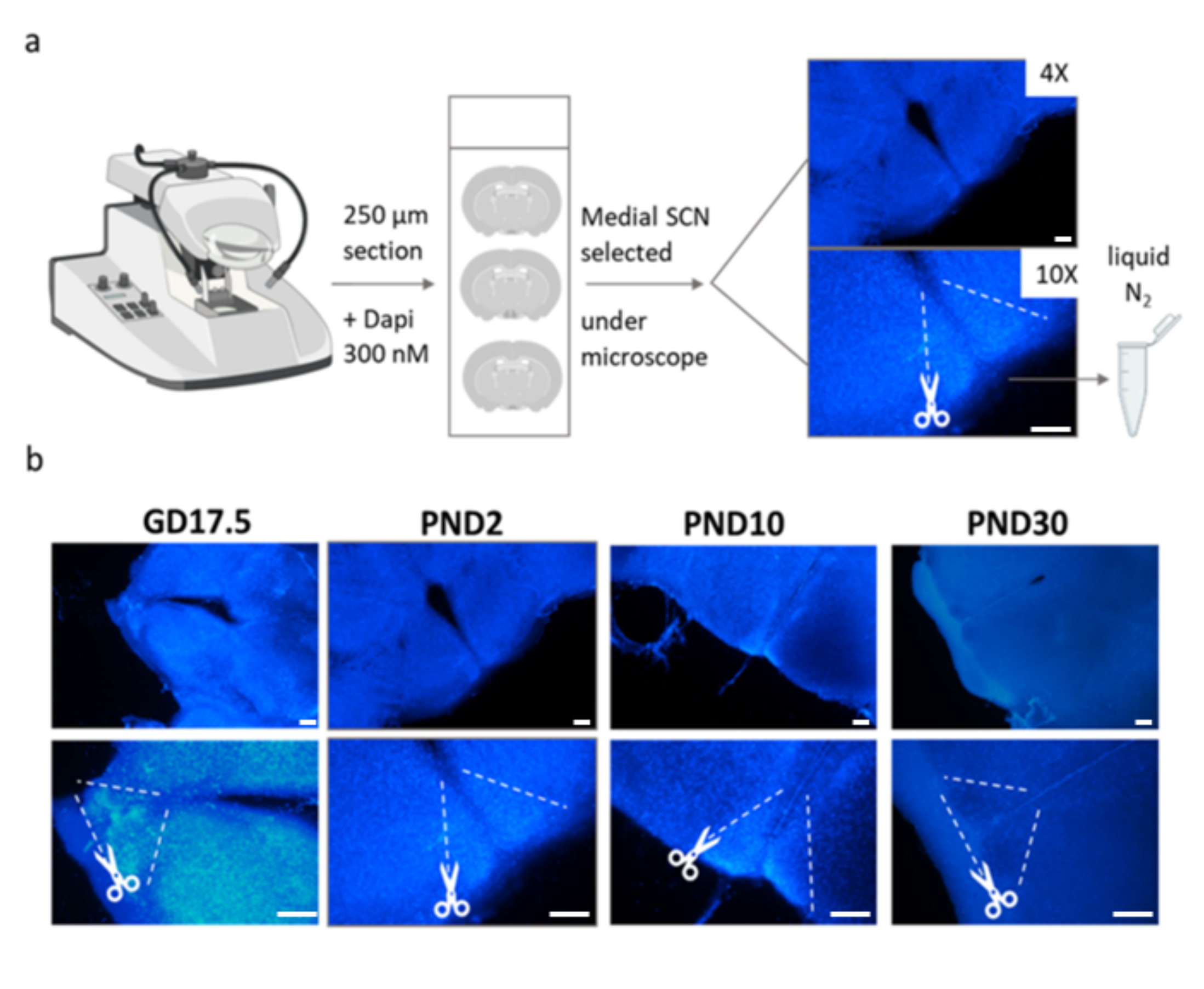

Supplement: S3 Fig — a) Brains were dissected and sliced in 250-μm thick coronal sections with a vibratome in ice cold HBSS 1×. To prepare slices from GD17.5 and PND2 fetus/pups, the brains were placed in a block of low melting agarose 4%, while PND10 and 30 brains were glued directly on the vibratome platform. At least 3 sections were placed on RNase-free glass slides, incubated 2 mins with a nuclei fluorescent marker (DAPI 300 nM) and observed under the microscope. The SCN from the medial section was chosen, dissected with a scalpel and frozen in liquid nitrogen. Created in BioRender. Administrator, S. (2026) https://BioRender.com/0y41lu2. b) Examples of sections dissected from all developmental timepoints, scale bars 200 μm. (TIFF) [file pbio.3003870.s003.tiff]

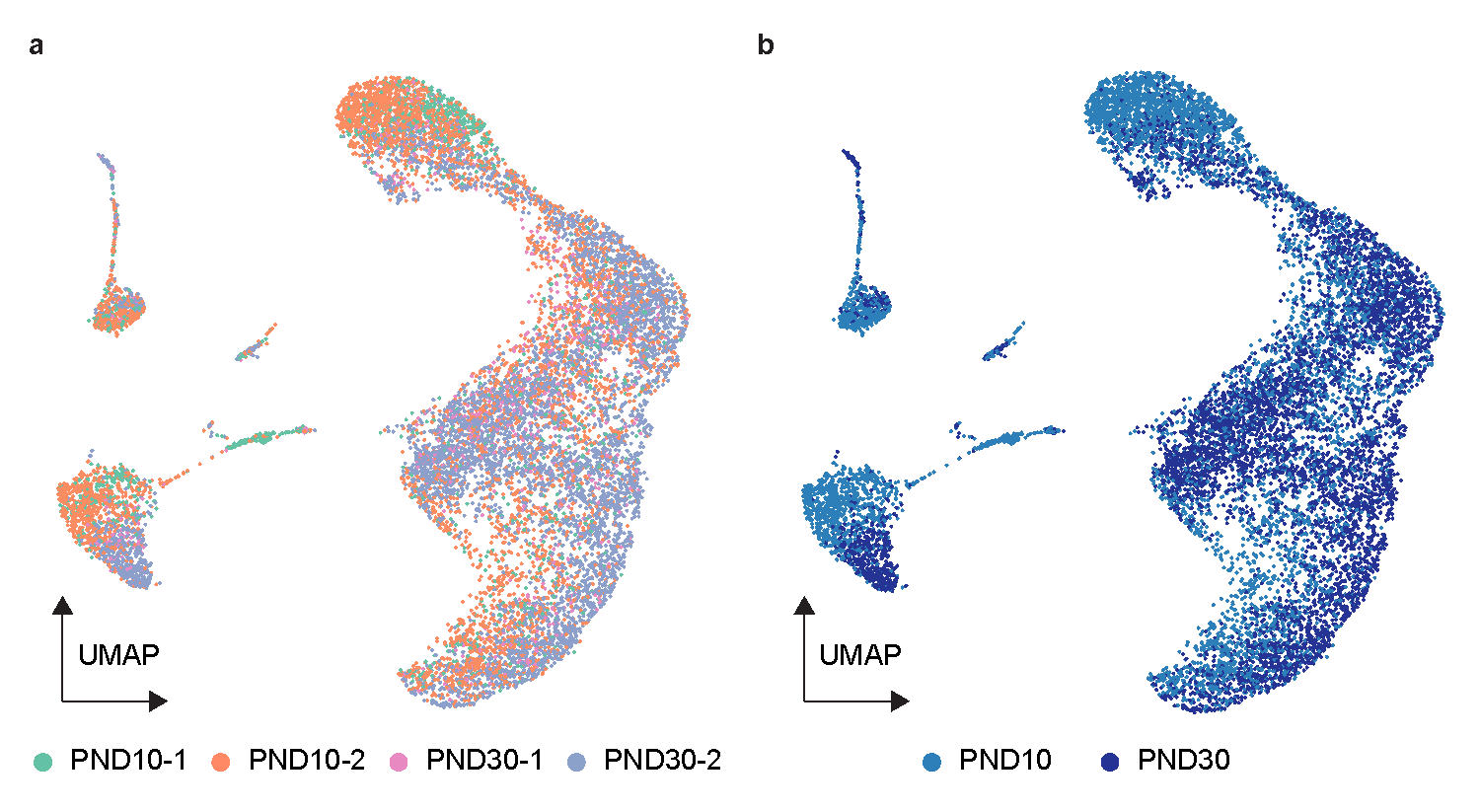

Supplement: S4 Fig — a) UMAP embeddings of two independent replicates of PND10 and PND30 merged without batch-correction, where cells are colored by individual experiment and developmental stage. b) Same UMAP embedding colored by developmental stage. Replicates are clustering together and within clusters timepoints are distinctly separated. Raw transcriptomic data can be found under GEO accession number GSE240803. (TIFF) [file pbio.3003870.s004.tiff]

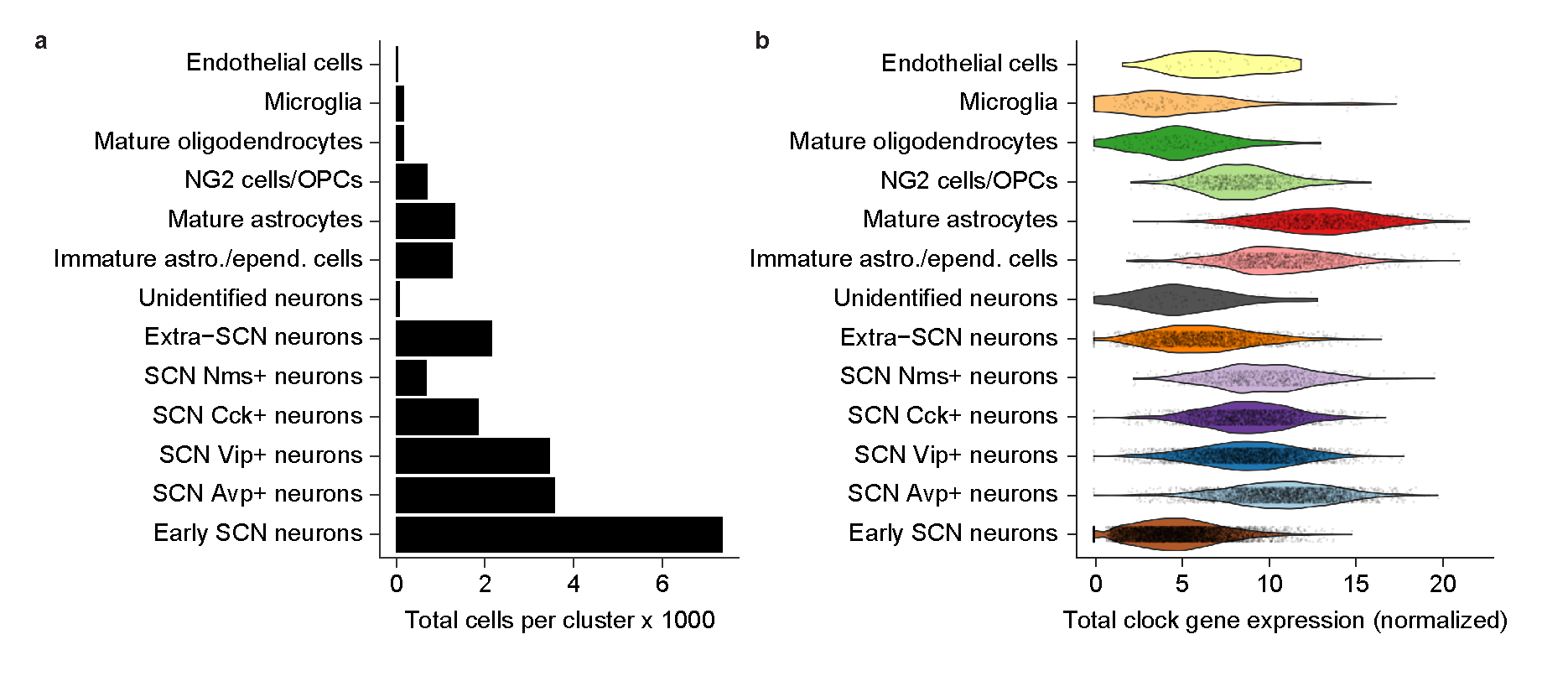

Supplement: S5 Fig — a) Cell type composition in the dataset. b) Violin plot showing the total expression of clock genes across the main clusters. Raw transcriptomic data can be found under GEO accession number GSE240803. (TIFF) [file pbio.3003870.s005.tiff]

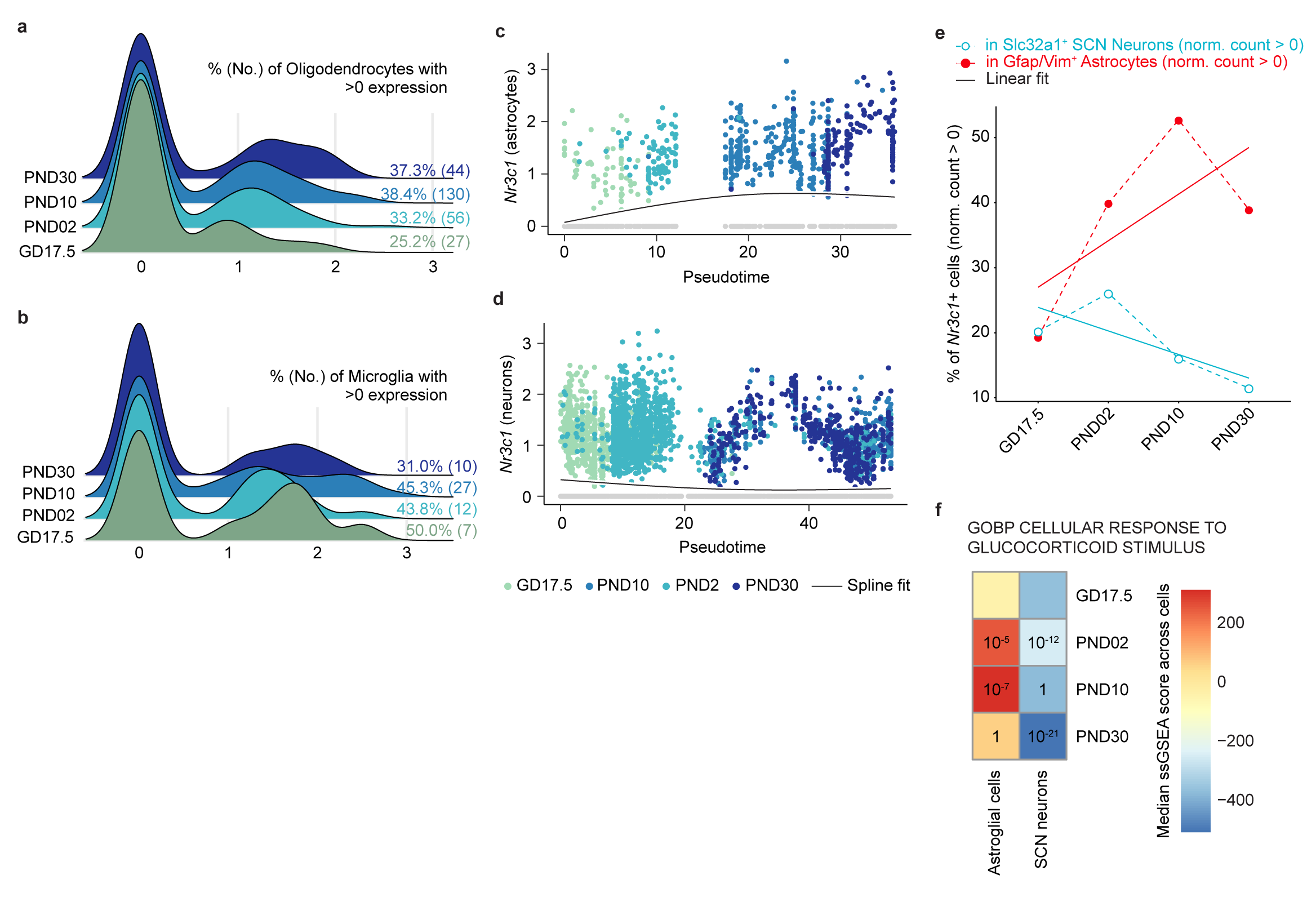

Supplement: S6 Fig — a,b) Density plot of the Gr expression in oligodendorcytes and microglia segregated by developmental stage. The percentage (absolute number) of cells with non-zero expression of Gr are indicated for each cell cluster. c) Gr expression in astrocytes and ependymal cells subset is represented along the developmental pseudotime trajectory (trajectory in the direction of ependymal cells was manually excluded from the analysis). d) Gr expression in the neuronal subset (excluding extra-SCN neurons and unidentified neuronal cluster) is represented along the developmental pseudotime trajectory. In c and d, colors represent developmental timepoints, except for cells with zero expression represented in gray, the solid line represents spline fit to the datapoints. e) Percentage of Gr+ cells co-expressing astrocytic markers (Gfap/Vim+ in red) or neuronal marker (Slc32a1 (Vgat)+ in cyan) are plotted for each developmental age, linear fit curves show the tendency of the developmental change. f) Median enrichment scores for the geneset segregated by the time points and cell types. Only astroglial cells (astrocytes, immature astrocytes/ependymal cells) and neurons (except extra-SCN neurons and unidentified neuronal cluster) are compared. EnrichIt with default parameters, except for using 10,000 groups and variable number of cores, was performed on the seurat-object. FDR adjusted p values <0.05 were considered statistically significant. Raw transcriptomic data can be found under GEO accession number GSE240803. (TIFF) [file pbio.3003870.s006.tiff]

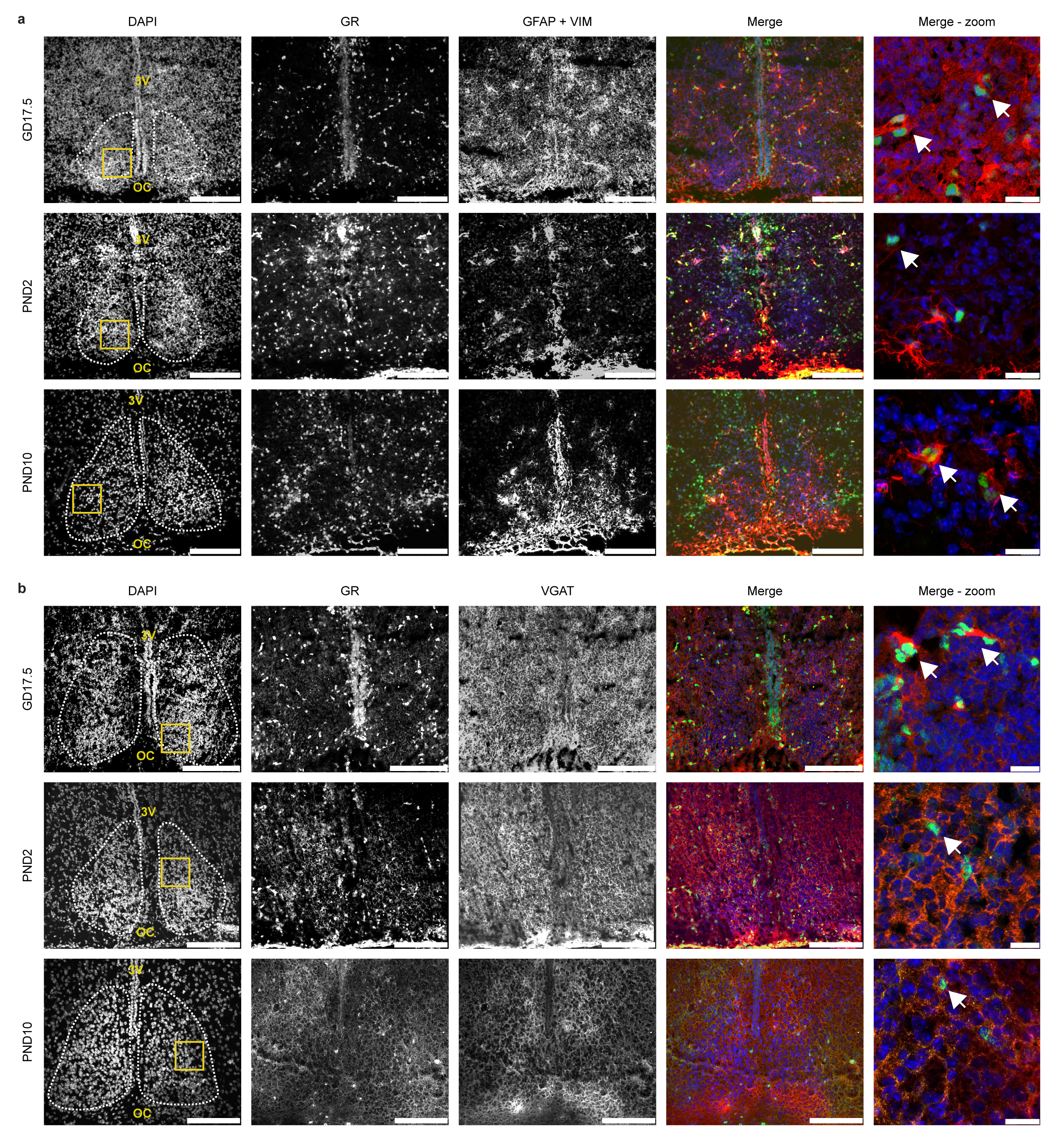

Supplement: S7 Fig — a) Representative immunohistochemistry confocal images of the SCN (DAPI), for GR (green) in astrocytes (GFAP/VIM+; red) for GD17.5, PND2 and PND10. The dotted white regions demarcate the SCN and the yellow squares the magnified areas. Scalebars correspond to 200 µm, except for the zoomed images, where it is 20 µm. Arrows highlight co-localization (GR and GFAP+VIM). b) Representative immunohistochemistry confocal images of the SCN (DAPI), for GR (green) in neurons (VGAT+; red) for GD17.5, PND2 and PND10. The dotted white regions demarcate the SCN and the yellow squares the magnified areas. Scalebars correspond to 200 µm, except for the zoomed images, where it is 20 µm. Arrows highlight co-localization (GR and VGAT). Staining was performed on at least three SCN sections per mouse and a total of three different mice per time point of both sexes. OC: optic chiasm, 3V: third ventricle. (TIFF) [file pbio.3003870.s007.tiff]

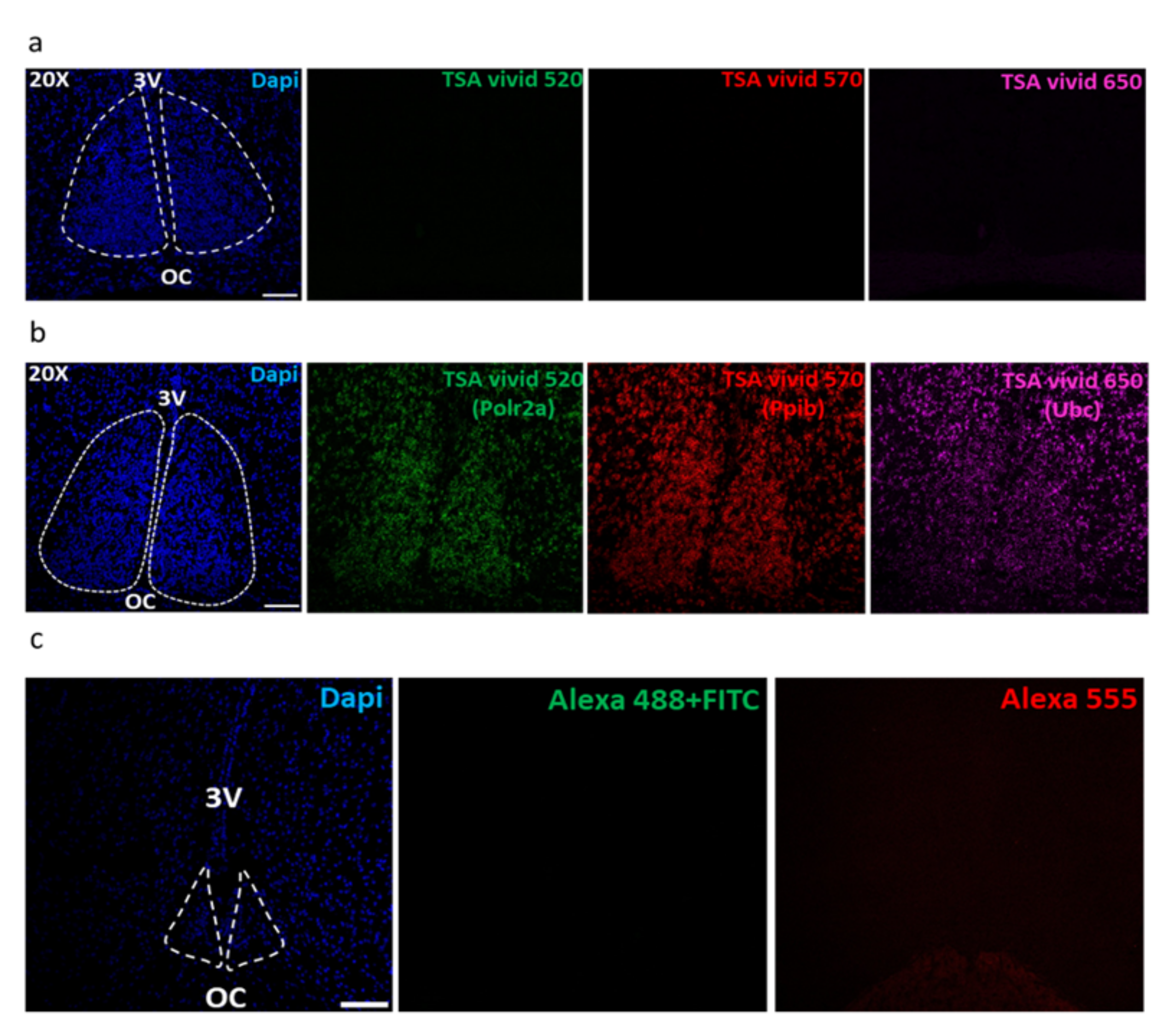

Supplement: S8 Fig — a) Representative confocal images showing the results of the RNAscope negative control performed following manufacturer’s instructions (negative probe followed by the three amplification steps and the three HRP-Channels/TSA fluorophore (TSA vivid 520, 570, and 650)/HRP-Blocker and counterstained with DAPI. b) Representative confocal images showing the results of the RNAscope positive control performed following manufacturer’s instructions (mixture of positive probes one/channel: Polr2a-C1, Ppib-C2 and Ubc-C3) followed by the three amplification steps and the three HRP-Channels/TSA fluorophore (TSA vivid 520, 570 and 650)/HRP-Blocker and counterstained with DAPI. c) Representative confocal images of the immunohistochemistry negative controls run without primary antibodies and incubation for 2 hours at RT in a dark chamber with the mixture of all three secondary antibodies (anti-mouse Alexa 488, anti-chicken FITC, and anti-rabbit Alexa 555). Scalebar correspond to 100 µm for all pictures. (TIFF) [file pbio.3003870.s008.tiff]

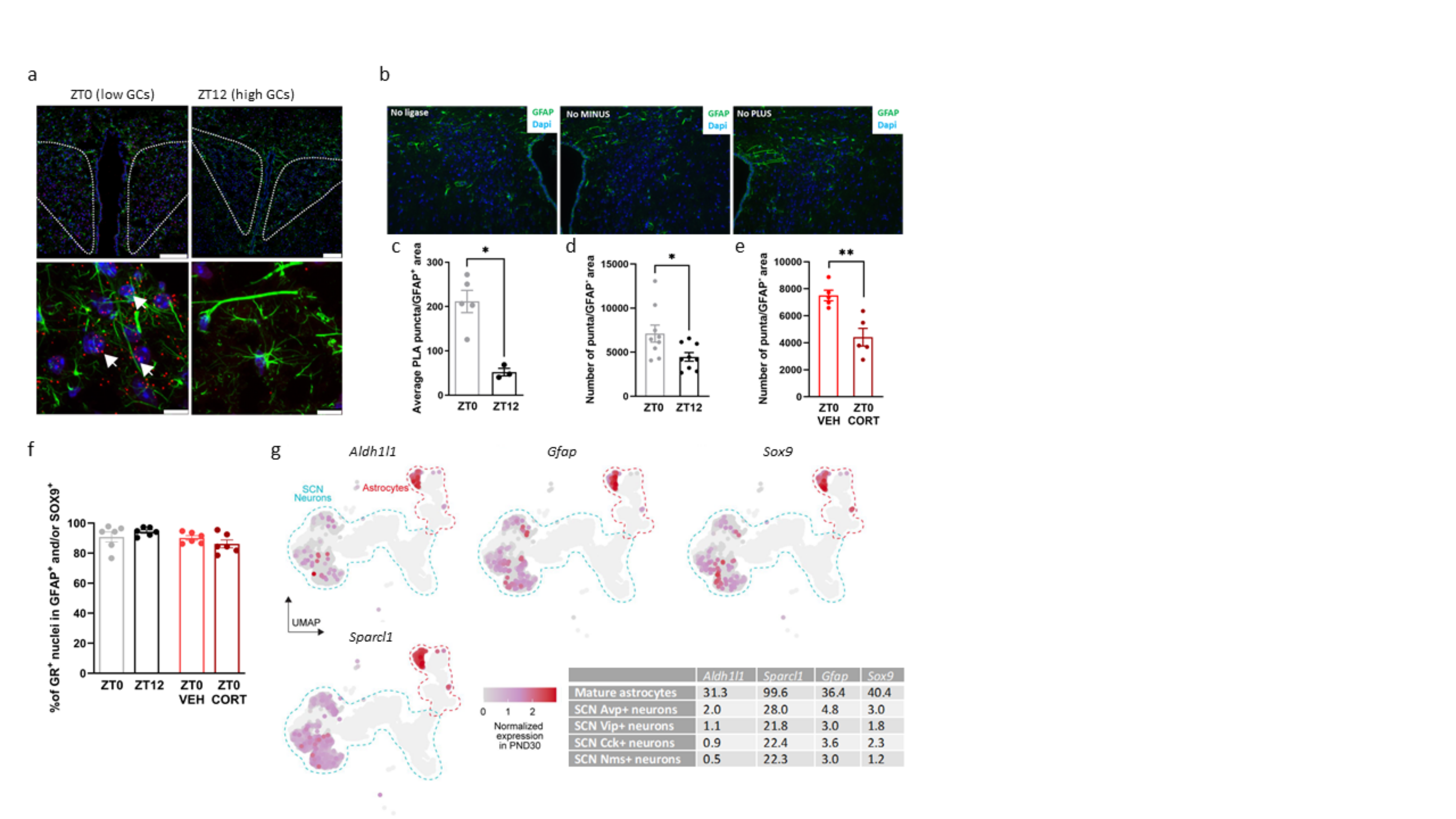

Supplement: S9 Fig — a) Proximity ligation assay in the paraventricular nuclei (PVN) as a positive control. Top: reconstruction of the PVN from pictures taken at 40× magnification (scale bar 100 µm). Bottom: zoom in (scale bar 8 µm). The dotted white regions demarcate the PVN. b) The negative PLA controls were run omitting either ligase, MINUS secondary probe or PLUS secondary probe as appropriate, no signal was observed. c) Quantification of the number of GR-HSP90 signal (puncta)/GFAP+area in the PVN at both timepoints (n = 3–5 mice/time point). Statistical difference was assessed by two-tailed Mann–Whitney, *p = 0.0357. d) Quantification of the number of GR-HSP90 signal (puncta)/GFAP− area in the SCN at both timepoints ZT0 (low GCs) and ZT12 (high GCs). Data passed normality test and statistical difference between ZT0 and ZT12 was assessed by two-tailed T test, t = 2.443, df = 16, *p = 0.0265. e) Quantification of the number of GR-HSP90 signal (puncta)/GFAP− area in the SCN one hour after subcutaneously injecting either VEH or CORT (5 mg/Kg body weight at ZT23). Data passed normality test and statistical difference between ZT0 VEH and ZT0 CORT was also assessed by two-tailed T test, t = 4.043, df = 8, **p = 0.0037. f) The percentage of GR+ nuclei co-stained either with GFAP, with SOX9 or with both astrocytic makers in samples from Fig 5. g) Expression of Aldh1l1, Gfap, Sox9, and Sparcl1 in the snRNA-seq dataset plotted in the same UMAP embedding as Fig 3a across different developmental stages (including only SCN neurons and astrocytes). Bottom: table showing the percentage of cells expressing each of the markers at PND30. Numerical data can be found in S1 Data file. (TIFF) [file pbio.3003870.s009.tiff]

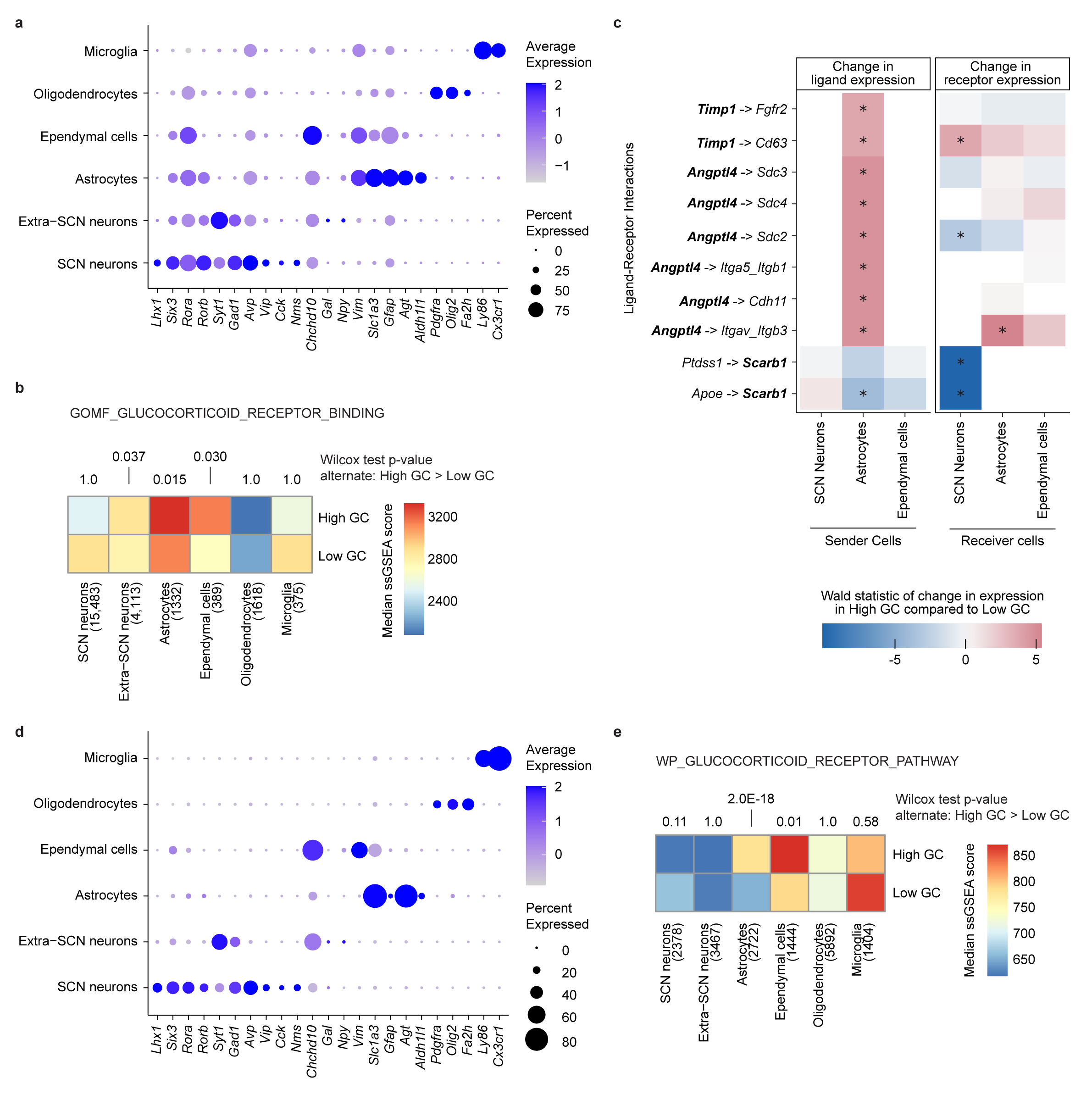

Supplement: S10 Fig — a) Data from Morris and colleagues (2021) was downloaded from a public repository and processed in the same fashion as our own snRNA-seq dataset, before filtering for only glial and neuronal cell clusters. Dot plot showing expression of the same markers used in Fig 2c (except epithelial markers) in Morris and colleagues dataset showing the presence of a similar set of cell types/clusters. b) Median enrichment scores for the geneset segregated by cell types and expected GC levels. Single-sided Wilcoxon test was applied to statistically compare scores within cell types between cells expecting high and low GCs. Corresponding p-values are displayed above the heatmap. c) Predicted ligand-receptor interactions between the three cell types where the gene in bold is a regulatory target of Gr and is differentially expressed in the expected high GC condition based on activation/repression relationship. Asterisk indicates adjusted p-value <0.05. d) Data from Wen and colleagues (2020) was downloaded from a public repository and processed in the same fashion as our own snRNA-seq dataset, before filtering for only glial and neuronal cell clusters. Dot plot showing expression of the same markers used in Fig 2c (except epithelial markers) in Wen and colleagues dataset showing the presence of a similar set of cell types/clusters. e) Median enrichment scores for the geneset segregated by cell types and circulating GC levels. Single-sided Wilcoxon test was applied to statistically compare scores within cell types between cells with high and low GCs. Corresponding p-values are displayed above the heatmap. Raw transcriptomic data can be found under GEO accession number GSE240803. (TIFF) [file pbio.3003870.s010.tiff]

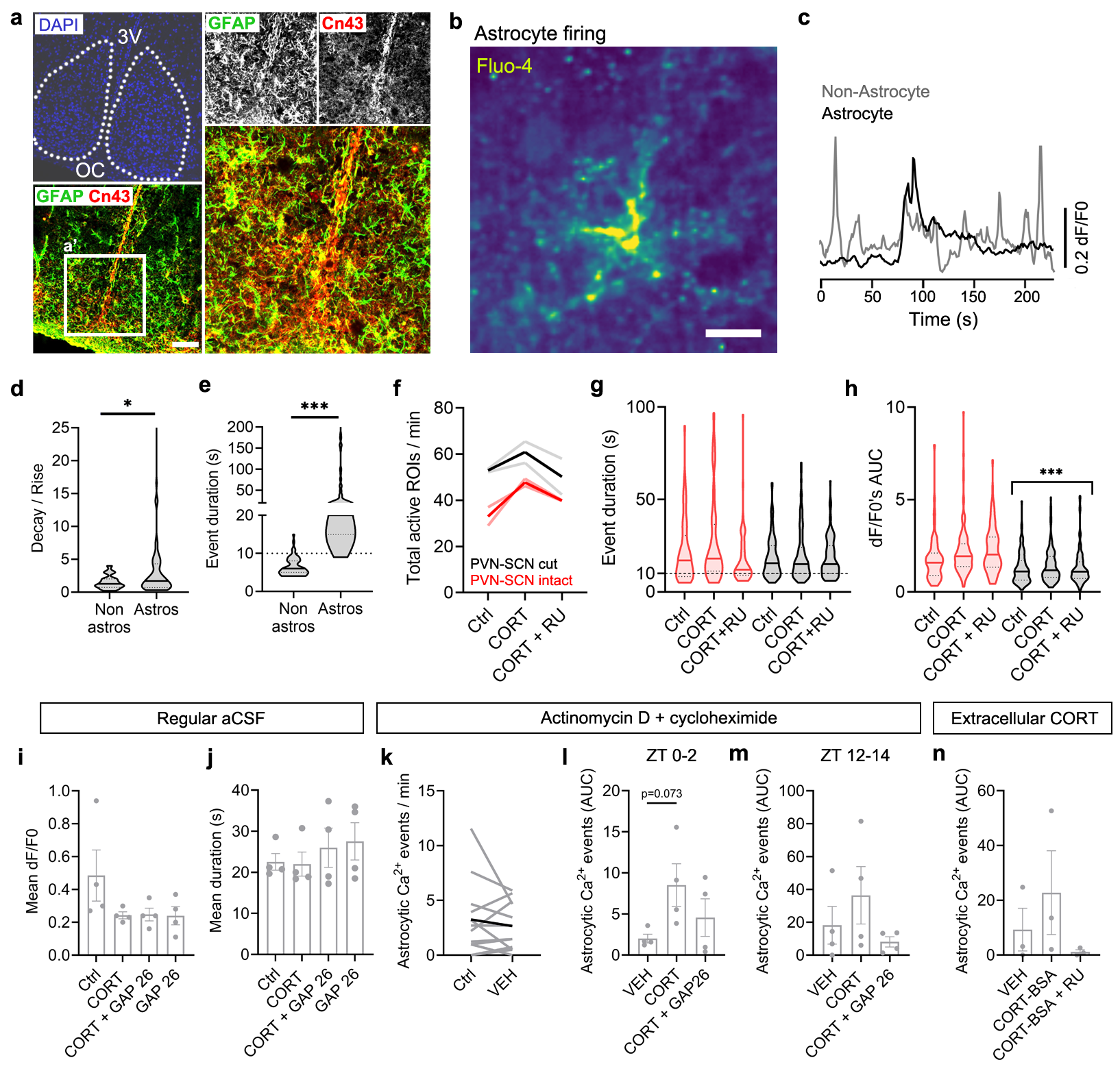

Supplement: S11 Fig — a) Representative immunohistochemistry confocal images of the SCN (DAPI), for Connexin 43 (Cn43 in red) and astrocytes (GFAP in green). The dotted white regions demarcate the SCN and the magnified area is indicated with a white square. Scale bar correspond to 100 µm. OC: optic chiasm, 3V: third ventricle. b) Astrocytes are identified based on their morphology. c) Further filtering was done at the analysis pipeline (i.e., cells with events shorter than 10 s are not considered). Representative overlapped astrocyte and non-astrocyte trace to better visualize the differential dynamics of the events included in the analysis. d) Decay (time from maximum to baseline)/rise (time from baseline to maximum) ratio of calcium events in astrocytes and non-astrocytes from the experiment in Fig 6g and 6h (n = 4 independent slices). Statistical difference was assessed by Mann–Whitney test, *p < 0.05. e) Duration of Ca2+ events from the experiment in Fig 6g and 6h. The dotted line indicates the duration threshold used to count astrocytic events (10 s). Statistical difference was assessed by Mann–Whitney test, ***p < 0.001 (n = 4 independent slices). f) Total number of active regions of interest (ROIs) in the SCN upon CORT treatment and when CORT binding to the GR is antagonized by co-incubation with RU486 1 μM (RU), in slices where the PVN–SCN connection was intact or cut (n = 3 independent slices per condition). g) Duration of Ca2+ events, the dotted line indicates the duration threshold used to count astrocytic events (10 s). h) area under the curve (AUC) of the amplitude of the fluorescence signal (dF/F0). Statistical differences were assessed by Kruskal–Walli’s test, ***p < 0.001 (n = 3 independent slices per condition). i,j) Mean amplitude and duration of astrocytic Ca2+ events from the experiment in Fig 6g and 6h (n = 4 independent slices). k) Number of astrocytic Ca2+ events comparison between baseline recording (Ctrl) and vehicle (aCSF containing 0.1% DMSO) is represented as [file pbio.3003870.s011.tif]
