# Supplementary material for: A developmental shift in glucocorticoid receptor expression preserves glucocorticoid sensitivity in the adult suprachiasmatic nucleus
Source: PLoS Biol. 2026 Jul 7;24(7):e3003870. doi: 10.1371/journal.pbio.3003870 (PMC13340777; doi:10.1371/journal.pbio.3003870)
Supplement: S1 Table — (DOCX) [file pbio.3003870.s014.docx]

**S1 Supplementary Table**

|  | **Description** | **Catalog #** | **Company** | **Country** |
| --- | --- | --- | --- | --- |
| **Antibodies** | | | | |
| Anti-GFAP | 1:200 | 14-9892-82 | Thermo Fisher Sci. | Massachusetts, USA |
| Anti-GR | 1:200 | AB183127 | Abcam | Cambridge, UK |
| Anti-Vimentin | 1:1000 | AB5733 | Sigma-Aldrich | Missouri, USA |
| Anti-Vgat | 1:200 | 131011 | SYSY Antibodies | Goettingen, Germany |
| Anti-Connexin 43 | 2 µg/mL | 71-0700 | Thermo Fisher Sci. | Massachusetts, USA |
| Anti-HSP90 | 1:100 | SC-13119 | Santa Cruz Biotech | California, USA |
| Anti-SOX9 conjugated with Alexa 647 | 1:100 | AB207677 | Abcam | Cambridge, UK |
| Donkey anti-Rabbit Alexa 555 | 1:500 | A32794 | Thermo Fisher Sci. | Massachusetts, USA |
| Donkey anti-Chicken FITC | 1:500 | SA1-72000 | Thermo Fisher Sci. | Massachusetts, USA |
| Goat anti-Mouse Alexa 488 | 1:500 | A11029 | Thermo Fisher Sci. | Massachusetts, USA |
| Chicken anti-GFAP | 1:1000 | PA1-10004 | Thermo Fisher Sci. | Massachusetts, USA |
| Donkey anti-chicken Alexa 488 | 1:1000 | 703-545-155 | Jackson ImmunoReseach | Cambridge, UK |
| **Kits** | | | | |
| RNAscope | RNAscope® Intro Pack for Multiplex Fluorescent Reagent Kit. Fresh Frozen (mouse) | 323130 | Bio-Techne | Oxfordshire, UK |
| TSA vivid fluorosphore | TSA Vivid Fluorophore Kit 650  TSA Vivid Fluorophore Kit 570  TSA Vivid Fluorophore Kit 520 | 7527  7526  7523 | Tocris | Bristol, UK |
| Chromium Next GEM Single Cell 3' Kit v3.1 | scRNA-Seq library preperation kit | 1000269 | 10X Genomics | California, USA |
| Qubit 1x dsDNA HS Assay | library concentration measurement | Q32851 | Invitrogen | Massachusetts, USA |
| Bioanalyzer Agilent High Sensitivity DNA Kit | library size distribution measurement | 5067-4626 | Agilent Technologies | California, USA |
| NextSeq 2000 P3 Reagents (100 Cycles) | sequencing reagents | 20040559 | Illumina | California, USA |
| PLA in situ red | Duolink in situ red detection reagents | DUO92008 | Merck | Missouri, USA |
| PLA anti-mouse | Duolink anti-rabbit PLUS | DUO92004 | Merck | Missouri, USA |
| PLA anti-rabbit | Duolink anti-mouse MINUS | DUO92002 | Merck | Missouri, USA |
| Corticosterone ELISA kit | Corticosterone quantification in mice plasma | ADI-900-097 | Enzo | Farmingdale, USA |
| **Reagents** |  |  |  |  |
| Prolong gold antifade reagent | Mounting media | P36930 | Thermo Fisher Sci. | Massachusetts, USA |
| Normal Goat Serum | Immunohistochemistry blocking | 5425S | Cell signaling | Massachusetts, USA |
| Normal rabbit serum | Immunohistochemistry blocking | 31883 | Thermo Fisher Sci. | Massachusetts, USA |
| DAPI (4',6-Diamidino-2-Phenylindole, Dihydrochloride) | DAPI staining | D1306 | Thermo Fisher Sci. | Massachusetts, USA |
| Low melting agarose | Vibratome block | A9414 | Sigma-Aldrich | Missouri, USA |
| HBSS | Buffer 1X | 24020-091 | Thermo Fisher Sci. | Massachusetts, USA |
| OCT Embedding Matrix | Cryostat block | 6478.1 | Carl-Roth | Karlsruhe, Germany |
| Paraformaldehyde | Cryo slices fixation | 0335.1 | Carl-Roth | Karlsruhe, Germany |
| Taq DNA polymerase | 50 UI/µL | A111103 | Ampliqon | Odense, Denmark |
| dNTP mix | 10 µM | R0192 | Thermo Fisher Sci. | Massachusetts, USA |
| IGPAL CA-630 | tissue lysis buffer | 56741-50ML-F | Sigma-Aldrich | Missouri, USA |
| Recombinant RNase Inhibitor | tissue lysis/nuclei suspension buffer | 2313B | Takara Bio | Japan |
| pluriStrainer Mini 20 µm | cell strainers | 43-10020-60 | pluriSelect Life Science | Germany |
| PLA blocking buffer | Included in DUO92002/4 | n/a | Merck | Missouri, USA |
| FluorSave | FluorSave Reagent | 345789 | Merck | Missouri, USA |
| D-Luciferin | Luminiscence recordings | L-2912 | Thermo Fisher Sci. | Massachusetts, USA |
| Millicell Cell culture inserts | Organotypic culture | PICM03050 | Merck | Missouri, USA |
| B27 | Serum free supplement | 17504044 | Thermo Fisher Sci. | Massachusetts, USA |
| Corticosterone | SCN slices treatment | 27840 | Sigma-Aldrich | Missouri, USA |
| PEG-400 | BioUltra, 400 | 91893 | Sigma-Aldrich | Missouri, USA |
| DMSO | Vehicle | 67-68-5 | Sigma-Aldrich | Missouri, USA |
| GAP26 | Connexin mimetic peptide inhibits connexin43 hemichannels | 197250-15-0 | Adooq Bioscience | Irvine, CA, USA |
| Pluronic | Permeabilizing agent | P6867 | Thermo Fisher Sci. | Massachusetts, USA |
| Fluo-4-AM | Calcium sensor | F14201 | Thermo Fisher Sci. | Massachusetts, USA |
| RU486 | GR antagonist | 475838 | Sigma-Aldrich | Missouri, USA |
| Actinomycin D | Transcription inhibitor | 1229/10 | Tocris | Bristol, UK |
| Cycloheximide | Translation inhibitor | 0970/100 | Tocris | Bristol, UK |
| CORT-BSA | Corticosterone conjugated with bovine serum albumin | 80-1062 | BioSynth | Staad, Switzerland |
