# Supplementary material for: A developmental shift in glucocorticoid receptor expression preserves glucocorticoid sensitivity in the adult suprachiasmatic nucleus
Source: PLoS Biol. 2026 Jul 7;24(7):e3003870. doi: 10.1371/journal.pbio.3003870 (PMC13340777; doi:10.1371/journal.pbio.3003870)
Supplement: Attachment — Submitted filename: Point-by-point response .docx [file pbio.3003870.s018.docx]

**Point-by-point response**

Reviewer’s and Editor’s comments (in black) are followed by the author’s responses (blue).

**Editorial comments:** Having discussed the reviews with the Academic Editor, we appreciate that the transcriptomic data are the strongest aspect of the study, and so we think the revision will largely need to focus on strengthening the physiological, mechanistic, and calcium imaging experiments.
While we think that some of the concerns should be relatively straightforward to address, other comments will require additional experiments and analyses. We think it will be essential to include the missing controls. For example, we agree with the reviewers that animal handling and injections could indeed induce corticosterone secretion, and naïve dams are not suitable controls. Similarly, changing the medium in astrocytic culture could trigger cellular responses, highlighting the need for vehicle controls in the Ca²⁺ experiments. The Ca²⁺ heatmap also appears problematic and will require more robust analysis and quantification. In addition, the bioluminescence assay requires substantial reinterpretation and reanalysis.

**Authors’ response:**

We thank the editors for the careful assessment of our work, below, we are providing responses for every comment. Following editors’ advice, we have focused the revision on strengthening the physiological, mechanistic, and calcium imaging experiments.

Specifically, we have:

1) Performed a new experiment to show that the animal handling and injection are not inducing any significant change in behavior. Please, see **response #2 and #43**.

2) Added vehicle controls to the Ca^2+^ experiments and compare to baseline controls. Please, see **response #12**

3) Performed three more experiments to confirm that CORT induces Ca^2+^ events in SCN astrocytes providing a better understanding on the mechanism underlying the effects. Please, see **response #15, #34 and #52**

4) Re-analyzed the data to show in a more robust way that synchronized Ca^2+^ events are more frequent upon CORT treatment. Please, see **response #14 and #33**.

5) Re-analyzed the data from the bioluminescence assay as suggested by reviewer#1. When data are analyzed this way, we still see changes in the same direction and we still find them to be statistically significant (please, see **response #16-20** and **#37**). The magnitude of the CORT effect, however, is markedly smaller with this analysis. An average phase delay of 2.12 ± 0.25 h (mean ± SEM) might not be biologically relevant. Due to a bottleneck in mouse supplies, we were unable to perform further bioluminescence experiments to increase the sample size. Moreover, during the period of revision, a paper was published showing that dexamethasone (an agonist of the GR) does not have an effect shifting the phase of the SCN clock in adults (PMID: 41314247).

Given these considerations, and in light of the strong and compelling molecular biology (and now the Ca^2+^ imaging) results, we would like to follow editors’ and reviewers’ advice regarding this data set. We would be inclined to remove figure 7 and associated supplementary material from the revised version. As reviewer #2 already commented, the manuscript would remain impactful even if bioluminescence data is deemphasized or removed.

Moreover, we have

6) Re-focused the discussion section for a better contextualization of our findings.

7) Added a paragraph explaining the limitations of our approaches in the discussion section (page 33 lines 700-713 and page 34 lines 714-734) of the revised manuscript.

8) Included all the references suggested by the reviewers.

9) Provided point-by-point responses to all reviewer’s comments.

We believe that the revised version of our manuscript has substantially improved, and we hope that you share our enthusiasm and consider our work for publication.

**Reviewer #1:**

Dear Dr. Smith,
Please find attached my review for the manuscript "The central circadian clock and the developmental timeline of glucocorticoids sensitivity" by Händler et al, submitted for consideration in PLoS Biology.
In the manuscript, Händler et al, detail cell-type specific changes in the expression of glucocorticoid receptors (GR) in the suprachiasmatic nuclei (SCN) of the hypothalamus through development.  The SCN, as the master mammalian coordinator of circadian rhythms, controls physiological and behavioural rhythms including the timing of glucocorticoid release from the which signals the beginning of the active phase.  It is largely accepted that GR activation during development and in early life helps to align the SCN to the correct environmental time, however, this sensitivity is thought to drop off as mice reach maturity to prevent feedback onto the SCN disrupting circadian rhythms preferentially aligned by the light-dark cycle and other cues. In the current manuscript, the authors seek to address whether this is true and present new evidence that the SCN may in-fact retain glucocorticoid sensitivity into adulthood.  Furthermore, they claim that this sensitivity is maintained by expression within the astrocytes of the SCN, and not the neurons.  A major technical tour-de-force in this manuscript is the developmental single-nuclear transcriptomic dataset (snRNA-seq), which will be of interest to the neuroscience community generally, but of particular interest to researchers interested in development, circadian rhythms and hypothalamic circuits.  However, the data that surrounds this dataset and the interpretation that the astrocytes alone mediate glucocorticoid responses in the adult SCN are less compelling.  It is this Reviewer's opinion, that the manuscript would benefit from substantial refocussing and restructuring, additional controls that have been omitted and additional analysis of existing data.  I would encourage the authors to reconsider their narrative and re-contextualise the dataset to present a balanced discussion of their findings.

**Response #1:**

We would like to thank the reviewer for the careful assessment of our work. We found all comments thoughtful and constructive and of great help to improve our manuscript.

**Main concerns**

Below, I have presented a detailed review:

**1) Developmental exposure to GCs**

**1.1)** The authors present an experiment where mice were given corticosterone either in-phase (in early morning) or out-of-phase (during the night) to stimulate circadian disruption and compare this to a control "naïve" condition where receive no manipulation.  The correct control here would be mice that had been injected with saline s.c. at the in-phase or out-of-phase timepoints.  This presents an important distinction, as all mice during pregnancy would have been handled and injected at the same gestational point.  Handling and injection would induce a basal stress response in the animals, including hormones beyond corticosterone that may be relevant to the phenotype observed i.e. can the phenotype be pinned to incorrect timing of a corticosterone surge, or are other hormonal factors at play here and (as is alluded to by the authors) would result in sleep disruption at certain points which need to be controlled for as well.  Data is presented later in the paper (Figure 4 b & e)
confirms that vehicle treatment in this way does not result in the same magnitude corticosterone elevation as exogenous corticosterone treatment.

**Response #2:**

We thank the reviewer for this important point (shared with reviewer #3). The two control groups suggested (injected with saline s.c./manipulated at the in-phase or out-of-phase timepoints) would have allowed us to dissect the effect of the manipulation from the effects of the CORT. However, with these experiments we aimed at assessing the long-term effects of the exact same manipulation at different times of day during the critical developmental window. The naïve group was generated with the only intention of comparing the response to jetlag/expression of the receptor to an undisturbed condition. In the revised version of our manuscript we have:

1) Checked that throughout the text we do not use any reference to a “control” group when referring to the naïve group and explained in a clearer way the reasons for comparing the prenatal manipulation to such a group. Please, find a comment in page 36 lines 768-770 of the revised manuscript.

2) Inspired by the reviewer’s comment, we have re-analyzed the data from Fig 4 (b and e) to directly compare the plasma corticosterone levels in naïve mice and in mice injected with vehicle (VEH) and corticosterone (CORT) at the same time of day (Fig 1R).

**Fig 1R: Level of corticosterone in naïve mice and in mice injected with VEH or CORT.** Plasma corticosterone levels in mice at ZT0 (grey), at ZT0 1 hr after being injected with VEH (red) and at ZT0 1 hr after being injected with CORT (dark red). Corticosterone measurements were done in duplicates using an ELISA kit and following manufacturers’ instructions (Enzo, Catalogue number ADI-900-097, USA). Data are expressed as mean ± SEM, normality was confirmed by Shapiro-Wilk test and analyzed by 1-way ANOVA. Significant differences were found F (2,15)=93.91, p<0.0001. Tukey’s multi-comparison test showed significant differences for ZT0 vs ZT0 CORT (****p<0.0001) and ZT0 VEH vs ZT0 CORT (****p<0.0001).

Statistical analysis shows that at ZT0 the naïve and the VEH-injected mice show comparable levels of corticosterone in plasma, suggesting that the VEH treatment does not result in a significant elevation of corticosterone levels. Although these data are informative, as the reviewer points out, we cannot exclude effects of other factors besides CORT.

3) Explored, as the reviewer suggested, the possibility that the injection/manipulation could cause changes beyond corticosterone such as disruption of sleep/wake cycles. We have always assumed that the effect of the CORT injection was probably not too impactful on the maternal sleep/wake cycles because the injections were done at ZT0 and ZT12, in both cases during at the transitions between the sleep and wake phases. However, since we never tested this before, we decided to perform an experiment to assess the effects of the injection/manipulation on the activity 6 h after. We used bouts of activity detected with infrared motion detectors as a sensitive readout for “other factors” that would disrupt sleep/wake phases. Please, note that this detection system is “noisier” than the running-wheel data.

**Fig 2R:**

**Fig 2R: Activity bouts before and after injection/manipulation at ZT0 and ZT12 compared to naïve mice.** We assessed the activity of 7 female mice kept in LD 12:12 and left undisturbed (naïve), injected with VEH (PEG-400 50% in PBS) or injected with CORT (5 mg/Kg b.w in PEG-400 50% in PBS) at ZT0 and ZT12. On top we showed the bouts of activity recorded over 4 days, the arrow indicates the injection time with VEH or CORT and the dotted area depicts the injection day. That day is zoomed at the bottom. In the bottom center we show the average of activity bouts the first 6 hours after the injection with VEH or CORT at ZT0 or at ZT12 in comparison to a naïve group. Data are expressed as mean ± SEM, normality was confirmed by Shapiro-Wilk test and analyzed by 2-way ANOVA using treatment (None, VEH or CORT) and time of day (ZT0 and ZT12) as factors. As expected, significant differences were found for the time of day F(1,36)=145.1, p<0.0001 but the treatments did not change significantly the activity at any of the time points. The activity did not show any difference days after the manipulation.

These new data provide evidence that the activity/rest patterns are comparable between the three groups suggesting that the injection/manipulation does not have a marked sleep effect in female mice. Therefore, we do not expect “other factors” to have a significant contribution on the offspring’s phenotype.

4) Explained our interpretation about the long-term effects of the prenatal manipulation on the offspring’s phenotype. Please, find changes in the introduction (page 6, lines 113-115), discussion (page 30 lines 646-647; page 31 lines 648-649; page 33 lines 700-706) of the revised manuscript.

**1.2)** In Figure 1b the activity profiles for mice whose mothers received corticosterone injections seem to entrain more weakly to the LD cycle before the jet-lag protocol is applied, especially when compared to the naïve mouse.  Is this consistent across the cohort?  Analysis should be presented as to how well the onsets are locked to the LD cycle across these animals i.e. are the mice properly entrained to the LD cycle at the beginning of the experiment or is this a feature of CORT administration during the gestational period?  Additional actograms should also be presented so that the precision of entrainment can be assessed.  Given the relatively modest slowing in response to the jet-lag protocol, it is critical that the error of entrainment to the LD cycle is assessed.  Could the modest slowing in the shift be that since the behaviour starts later in LD in these animals, it therefore takes slightly longer to catch up to the expected phase advance and represents a difference in entrainment rather than ability of the network to shift?  A minor point regarding this data is that time is presented as solar (Daytime, hrs) when it should conventionally be presented as zeitgeber time (ZT) relative to the LD cycle.

**Response #3:**

We thank the reviewer for this comment, we agree that a weak entrainment to the LD cycle could impact the speed of re-entrainment after a shift in the LD cycle (concern is shared by reviewer #3). In the revised version of our manuscript we have:

1) Added a new supplementary figure (S1 Fig) including the actograms of all the mice included in Fig 1. Each running-wheel actograms shows the locomotor activity before and after jetlag (naïve n=14, out-of-phase n=15 and in-phase n=16), both sexes are included. On the right we show the 24-hour profile of locomotor activity in LD 12:12 (S1a Fig) and a zoom-in on the onset of activity in the LD transition (S1b Fig). In both cases, the running-wheel activity of 10 days prior to jetlag was averaged for each individual animal, the patterns of activity nicely overlap, no differences are observed between the groups. We also assessed the onset of activity the day before of the jetlag (S1c Fig), again no differences were found at the onset suggesting that the speed of re-entrainment after jet-lag does not depend on a weaker entrainment to the LD prior to the shift. Please, find the new supplementary figure and changes in the results (page 8 lines 150-152) of the revised manuscript.

2) Changed, in the main figure one, of the selected actograms (in-phase group) to provide a more representative example of the whole cohort.

3) Presented the activity data as Zeitgeber time (ZT) relative to the LD cycle before the phase shift in both Fig 1 and S1 Fig, following the reviewer’s suggestion.

**1.3)** In Figure 1c, the data presented shows an asterisk, presumably demarking significance between the curves.  There is no mention in the text or legend as to the test performed here and the resulting significance.  Likely, this would come from a two-way ANOVA with gestational experience and time post-phase shift as factors.  The fit to the data is presented as a single line with error in the y-axis direction for the means.  Details as to the error of the curves fitted should be presented as a dashed CI or similar.

**Response #4:**

We thank the reviewer for this comment, we apologize for the mistake. The asterisk and the dotted line in Fig 1c referred to the PS50 shown in Fig 1d. In the revised version of our manuscript, we have deleted the asterisk and added PS50 in Fig 1c.

**1.4)** In Figures 1e & f, a critical point here is that the authors present evidence that there is a reduction in GR/GFAP co-localisation in the out-of-phase offspring. They use this as the first evidence that the astrocytes are what are important in carrying this change in phase-shift dynamics. However, they do not mention whether there is a general decrease in GR staining overall across the SCN of these animals, or whether it is specific to the astrocytes.  This is a critical point - is the modest phenotype that they measure here due to a reduction specifically in the astrocytes, or due to a general reduction in GR across the circuit as a whole?

**Response #5:**

We thank the reviewer for raising this important point. We have:

1) Quantified nuclear GR in the whole SCN in all the animals from Fig 1e and f. We did not find a significant difference between the groups (Fig 3R). We observed that the trend for the whole SCN follows the same direction as for SCN astrocytes alone, suggesting that astrocytes might be the main contributors of GR expression. This figure is included as S1e Fig in the revised manuscript.

**Fig 3R**

**Fig 3R: Quantification of the expression of GR in the whole SCN**. Data are expressed as mean ± SEM (n=8-12/group), passed normality test and statistical difference was assessed by one-way ANOVA, F(2,27)=2.447, p=0.1055, followed by Holm-Sidak’s multiple comparison test (naïve vs out-of-phase, p=0.1127, out-of-phase vs In-phase, p=0.2244).

2) Introduced changes throughout in the manuscript to tone down the claim that the astrocytes are the only cells on the adult SCN expressing GR.

**2) Single Nuclear Transcriptomics

2.1)** This dataset is the strongest part of this manuscript. The developmental snRNA-seq dataset will be a valuable tool for the wider community and the mapping back of the markers to the snRNAseq UMAP is a nice touch.  For the staining, the insets chosen focus on the periphery of the SCN, rather than highlighting expression within the SCN itself - it would be nice to see insets from elsewhere in the SCN as well.

**Response #6:**

We thank the reviewer for this positive assessment. In the revised version of the manuscript we are providing 4 zoomed-in images per developmental time point depicting the SCN, the insets are now numbered to allow an easy localization in the overview pictures (Fig 2e).

**3) GR expression is limited to astrocytes**

**3.1)** Beyond the general snRNAseq dataset itself, the authors focus on Nr3c1 (Gr) transcript expression which is mapped to their integrated UMAP plots in Figure 3a, revealing that as the SCN matures, Gr expression becomes more apparent in the astrocyte population.  This is confirmed by the ridge plot analysis in Figure 3b that shows that the percentage of astrocytes expressing Gr transcript increases along the trajectory of SCN development.  The authors allude to this representing a cell-type specific switch where the expression of GR goes up in the astrocytes and down in the neurons.  However, it appears that the ridge plot only shows how much the percentage within that cellular population changes, but not the actual numbers of cells expressing GR within the SCN.  Thus, even though it looks like there is a dramatic reduction in the proportion of Gr expressing cells in the dataset at PD30 (8.9% neurons expressing Gr versus 36.2% astrocytes expressing Gr), when you consider that
there are around 3x the number of neurons compared to astrocytes these numbers become much less dramatic when considering the absolute differences.  This is confirmed by the UMAP plots in Figure 3a, where the neurons appear to show a higher density of positive signal (albeit at a lower level of expression), which can also be seen in the pseudotime plots in Supplementary Figure 4.

**Response #7:**

We thank the reviewer for this comment and understand the concern. Therefore, we have:

1) Discussed the issue about the levels of expression of a transcript and the number of cells expressing it in the results (page13, lines 260-269) of the revised manuscript. We have also mentioned technical limitations of the experimental approach in the discussion (page 34, lines 721-725) of the revised manuscript.

2) Changed Fig 3a to include normalized *Gr (Nr3c1)* expression in microglia and oligodendrocytes clusters and, in consequence, made changes throughout the manuscript to tone down the claim that the astrocytes are the only cells in the adult SCN expressing *Gr*.

3) Extracted from the data set the number of cells expressing *Gr*. These numbers are now in brackets next to the percentages shown in Fig 3b, note that we have "corrected" to total numbers of cells per time point being 5,000 for all. **Interestingly, at PND10 the number of astrocytes expressing** Gr **exceeds the number of neurons expressing** Gr**, despite there being three times as many neurons as there are astrocytes in the SCN from this developmental time point on. In comparison, at PND30, while the absolute number of astrocytes expressing** Gr **is lower than that of neurons, the ones that do express *Gr* appears to have increased expression.** Please, find the changes in the results (page 13 lines 260-269) and in the figure caption of the revised manuscript.

4) Added, in S5a,b Fig, ridge/density plots of *Gr* expression in microglia and oligodendrocytes segregated by developmental stage. Analogous to the plots presented in Fig 3b, the percentages (and total numbers) of cells with expression above 0 is indicated for each cell cluster. Please, find changes in the results (page 13, lines 269-271) of the revised manuscript.

**3.2)** The data in Figure 3c shows a very dramatic reduction in the %GR-expressing cells co-stained for VGAT. This is higher than I would expect from the ridge plots and UMAP where it appears that there should be equal or only slightly fewer absolute numbers of neurons expressing the Gr transcript at PND30. It therefore presents a discontinuity within this dataset.  Has this analysis been done using the snRNAseq data i.e. co-expression analysis for % of cells with GR expression that also have these specific cell-type markers?  Do these follow the same trend?  This would bolster the argument that there is a cell-type specific switch.

**Response #8:**

We thank the reviewer for this comment. When comparing ridge/density plots from the snRNASeq data set and the immunohistochemistry we see a similar trend however, we cannot directly compare absolute numbers of cells expressing the receptor. We agree that the trend visually appears to be less dramatic in the ridge plot. Therefore, we have:

1) Explained the advantages and limitations of both approaches (snRNAseq and immunohistochemistry) to detect co-expression of *Gr* in specific cell-types. Please, find changes in the discussion (page 34, lines 721-725) of the revised manuscript.

2) Toned down the claim of a cell-type specific switch and focused instead on the trend of these changes along the developmental trajectory, which is going in the same direction independently of the approach used. Please, find the changes in the results (page 14 lines 281-284) of the revised manuscript.

2) Followed the reviewer’s suggestion and used the snRNAseq data set to assess the percentage of cells co-expressing *Gr/Vim+Gfap* and *Gr/Vagt*. We have added S5e Fig showing a similar trend of these changes along the developmental trajectory.

**3.3)** Regardless of whether more astrocytes or neurons express GR in the adult SCN, the main conclusion drawn from the dataset in the text is: "At the protein level, we also found GR co-localising with astrocytes (GFAP+, Fig 3e) but not with neurons (VGAT+, Fig 3f)."  This is in direct contradiction with the data presented in Fig. 3c, which shows that ~20% of GR is in neurons.  Furthermore, in the PLA experiment, as shown in Supplementary Figure 7, far more puncta are found in non-GFAP areas than are in GFAP areas.  This is confirmed by Figures 5a & c, where only between 20-30% GR signal is co-localized to astrocytes (SOX9) - suggesting that other cells in the SCN express GR more widely than the astrocytes.  From the snRNA-seq dataset, the best evidence is that it should be localised to the neurons as they are the only other cell-type that express Gr transcript, indeed this is confirmed by the GO term analysis (Supplementary Figure 4) where "…the GO biological term Cellular
response to glucocorticoid stimulus were maintained at low levels in neurons".

**Response #9:**

We thank the reviewer for this comment. In the revised version of our manuscript we have:

1) Changed the statement that now reads: “However, we did not observe co-expression with *Syt1* probes, a gene expressed exclusively in neurons (Fig 3d). At the protein level, we found a significantly higher percentage of GR^+^ cells co-expressing the astrocytic marker GFAP and VIM (Fig 3e) than co-expressing the neuronal marker VGAT (Fig 3f)”. Please, find the changes in the results (page 16, lines 325-328).

2) Introduced the changes already mentioned in **response #7 and 8**

3) Included a paragraph explaining this important point. As suggested by the reviewer, we have used two alternative approaches to assess the activation of GR by peripheral GCs. The PLA assay did not allow us to confirm that the activation of GR was astrocyte-specific because we found a significant difference in the number of PLA puncta in GFAP negative areas. Since only around 37% of the cells clustered as SCN astrocytes express *Gfap,* we used an alternative strategy to capture a larger percentage of SCN astrocytes. Please, find changes in the results (page 19 lines 386-390) of the revised manuscript.

Please, also find below Fig 4R showing feature plots and a table indicating the percentage of cells expressing the main astrocytic markers and their specificity. We have included this figure on the revised version of the manuscript (S8g Fig).

**Fig 4R:**

**Fig 4R:** Top: Expression of *Aldh1l1* (a), *Gfap* (b), *Sox9* (c) and *Sparcl1* (d) in the snRNA-seq dataset plotted in the same UMAP embedding as Fig 3a across different developmental stages (including only SCN neurons and astrocytes). Bottom: table shows the percentage of cells expressing each of the markers at PND30.

With these data we conclude that counterstaining astrocytes with ALDH1L1 would give us a similar result as GFAP because both markers are expressed in a similar percentage of SCN astrocytes. Moreover, others have shown that *Gfap* and *Aldh1l1* are co-expressed in SCN astrocytes (PMID: 28343966). Hevin (*Sparcl1*) could be a very good marker for astrocytes but its expression is not specific (PMID: 30656447). We found that *Sox9* is expressed in 40% of the cells clustered as SCN astrocytes in our data set at PND30 and that in combination, *Gfap* and *Sox9,* would allow us to detect around 80-90% of these cells in the SCN. Therefore, with a triple immunohistochemistry we were able to assess the percentage of GR+ nuclei that were also stained with either GFAP or SOX9 or both.

While revising the data, we realized that the way in which we labeled the Y axis in Fig 5a,c was misleading and that this could have contributed to the reviewer’s concern. What we represented is the percentage of all nuclei (DAPI^+^) that counterstain with GR to represent the activation of the receptor by its presence in the nuclei. To us it made sense that the percentage of GR^+^ nuclei was around 20-30% considering that it is mainly expressed in astrocytes and astrocytes represent around 20-30% of the SCN cells. However, we should have also shown the percentage of cells co-stained with GR^+^ and either GFAP or SOX9 or both astrocyte makers. This is, in average for all the groups and treatments around 90 % (Fig 5R).

**Fig 5R:**

**Fig 5R:** The percentage of GR^+^ nuclei co-stained either with GFAP, with SOX9 or with both astrocytic makers.

We apologize for this mistake and would like to thank the reviewer for the careful review that allowed us to correct this mistake at this stage. Please, find the changes in the results (page 19, lines 397-401) of the revised manuscript. We have also added the above figure as a supplementary figure (S8f Fig) and changed the Y axis label, it now reads “% of GR^+^ amongst all nuclei” in Fig 5a,c.

Despite these efforts, and in agreement with the reviewer’s concern, we still cannot attribute the responses to peripheral GCs to astrocytes alone. Therefore, we have toned down the original claim throughout the manuscript as suggested and focused on providing stronger evidence on the effects that GCs have on the SCN.

**3.4)** The claim that astrocytes are the sole important party to mediate GC responses in the adult SCN is therefore not backed up by the evidence presented across various experiments.  I see no reason that the authors need to discount the presence of extra-astrocytic expression of GR within the SCN.  The focus solely on astrocytes detracts from two important conclusions that could be drawn from the manuscript: 1) Astrocytes in the SCN express GR (in addition to the neurons), and 2) the SCN remains responsive to GC signals in adulthood. It would be better if the authors refocused the manuscript to acknowledge and explore these points.  This is of interest because the rest of the data shows SCN sensitivity to GC (as evidenced nicely in the PLA and GR-staining experiments in Figures 4 and 5 both in and outside astrocytes), suggesting that GR is functional within the SCN cells at this developmental timepoint, contrary to expectations from other published studies.  The inclusion of the neurons here allows for a nice compare-and-contrast as an internal control to show that the same activation response is seen in both cell-types.  This leads to further interesting questions: How might astrocytic- and neuronal-sensitivity to GCs differ or be similar? Is there cellular redundancy built in here, or is there a push-pull in the way astrocytes and neurons interact within the SCN network?

**Response #10:**

We thank the reviewer very much for this detailed, thoughtful and constructive criticism. We completely agree that the manuscript has benefited from re-structuring and re-focusing to stress our main findings. We hope the reviewer shares our enthusiasm about the new version of our manuscript.

**4) Calcium imaging in SCN slices**

**4.1)** The calcium imaging is a nice addition to try and understand the response of the astrocytes to GC exposure. The authors have been very careful to try and exclude the influence of neuronal activity within their dataset, however, they may be missing important astrocytic responses that make interpretation of the data is difficult.

**Response #11:**

We thank the reviewer for this comment. We are aware that astrocytes display a complex repertoire of Ca^2+^ events that evolve in space and time within single astrocytes and across astrocytic networks. Unfortunately, the experimental tools that we have used here have several limitations and do not allow us to describe the astrocytic physiology in full. We agree that we might be missing interesting data and therefore we are currently working on improving these tools for future experiments. In the revised version of our manuscript we have:

1) Performed experiments including missing controls

2) Performed experiments to support, with our own data, the reasoning behind the analysis strategy

3) Performed experiments to provide mechanistic details about the effects of CORT on the SCN

4) Added a paragraph in the discussion section to acknowledge the technical limitations of our approach.

Please, see the specific responses below.

**4.2)** First, no vehicle control application was performed, so the response of astrocytes in the presence of an intervention has not been quantified i.e. does manipulation of the recording medium change astrocyte responses relative to baseline?

**Response #12:**

We thank the reviewer for this comment. We did not include a vehicle control initially because we did not change the recording medium, the perfusion in the microscope chamber was constant at 0.5 mL/min and we just stopped the pump, changed the “in” tubing into different solutions containing the drugs at the final concentration and turned the pump on again immediately. Before each experiment, while setting up the perfusion system we calculated exactly how long it takes for the solution to reach the chamber. Recordings started after that time to ensure that the final concentration of the drug was constant in the chamber. We apologize for the mistake in the results and methods section of the original version of our paper. Please find the changes in the results (page 22 line 459-461) and in the methods (page 49 line 1051) of the revised manuscript.

Following the reviewer’s suggestion, in the new round of experiments performed during the revision of the paper (11 mice) we included a vehicle application and compare it with baseline. Baseline (Ctrl) recordings were performed with aCSF perfused in the recording chamber while vehicle (VEH) recordings were performed with aCSF containing 0.1% of DMSO (maximum concentration of DMSO across treatments).

We did not observe significant changes in the number of astrocytic events/min. Please, find changes in the methods (page 50 lines 1064-1066). We included the Fig 6R in the supplementary figures (S10k Fig).

**Fig 6R:**

**Fig 6R:** Number of astrocytic Ca^2+^ events is represented (independent experiments, n=11 mice, in grey) and as mean (in black). Data passed normality test and statistical differences were assessed by two-tailed paired Wilcoxon test, p=0.6533.

**4.3)** Second, the authors have set a very conservative threshold for detecting Ca2+ transients which limits most of these recordings to somatic Ca2+ activity, while missing Ca2+ activity in the astrocytic processes.  While I understand that the authors wanted to be conservative in their approach, the responses in the astrocyte soma and processes have been shown to be complex in their integration. Ca2+ activity in the processes, close to the membrane, contributes substantially to waves of activity across the astrocytes as well as between cells and is mediated differently to Ca2+ activity in the soma (e.g. PMID26814587, PMID36937570, PMID40419752).  As that is the focus of this experiment, I would encourage the authors to re-analyse this data with a view to better discriminating the response in the processes if possible.  This may increase the number of quantifiable Ca2+ events across the SCN as currently they are very low (5-15 total events, across ~5 astrocytes in a single recording interval) and therefore make it difficult to draw conclusion.

**Response #13:**

We thank the reviewer for this comment and we agree that with this conservative threshold, we are describing events in the soma and the main processes. Therefore, considering the reviewers’ comments we have re-focused the discussion on the effect of GCs on the SCN, acknowledge the possibility that SCN neurons, microglia and oligodendrocytes might also receive and respond to peripheral GCs and explain the technical limitations of our approach. Additionally, in the revised version we have:

1) Generated data to support the reasoning behind considering only events longer than 10 s. We run 3 experiments in which we incubated the acute slices with sulforhodamine 101 (SR101), a dye that preferentially labels astrocytes (PMID: 35199029). In this way we were able to identify events from astrocytes with more specificity and assess the length of the Ca^2+^ events in SR101-positive cells. As shown in Fig 7R, the astrocyte-like ROIs show Fluo-4 signal in the soma and the main processes, and we can confirm that our threshold of 10 sec detects almost 90% of the events in SR-101-positive cells.

**Fig 7R:**

**Fig 7R**: Astrocyte event identification: Left: Representative picture of an active (i.e.: high Fluo-4 signal in green) astrocyte that is labelled with Sulforhodamine 101 (1 μM in ACSF, S7635 Sigma Aldrich), scale bar 2 μm. Right: Dispersion of the length of the Ca^2+^ events in sulforhodamine + astrocytes, almost 90% of the astrocytes show events longer than 10s.

2) Explained our data in the context of the limitations of the experimental approach that does not allow us to describe the astrocytic physiology in full or to detect more astrocytes in the field of view. The main limitations are:

- **Ca^2+^ sensor**: We are aware that using genetically encoded Ca^2+^ sensors could have provided an increased sensitivity to detect events and specificity restricting its expression to astrocytes. However, for this set of experiments acute slices were prepared from wild-type mice of 20-30 days of age in LD12:12 conditions to match the transcriptional data. The introduction of a calcium sensor like GCaMP could have been done using a viral approach (i.e., introducing it with an AAV vector during early postnatal life) or using a mouse line (i.e., GFAP-GCaMP), in both approaches the chelating activity of the sensor could have had an impact on the development of the SCN. Therefore, we decided to use a cell permeable sensor. On the one hand, Fluo4-AM has several advantages such as: a high signal-noise ratio, low concentration requirement during the slice incubation and an excitation wavelength well-suited for our laser. On the other hand, Fluo4 has several limitations such as: it only detects events in the soma and in the large processes, it is not specific for astrocytes (although it tends to label a larger proportion of astrocytes compared to other cell types, see PMID: 22027012), it does not penetrate more than 60-70 μm from the surface of the slice.

- **Microscope:** We aimed at scanning the largest xy area possible of the SCN slice because the total number of astrocytes is low and we wanted to capture the global effects of CORT. Therefore, with our setup, 3D scanning was not possible because this would have increased significantly the total time to acquire a field of view, reducing even more the capacity to detect fast events. Thus, images were acquired at a speed of 1 Hz, events faster than 1 second could not be detected. Furthermore, without the use of a high-speed camera (PMID: 39804104), most neuronal fast events are lost due to the slow nature of the non-resonant scanner of our setup.

- **Expected number of astrocytes in the field of view:** We made a rough estimation of the number of astrocytes that we should see, and we cannot expect more than 5-10 astrocytes. We reasoned that the whole SCN has around 6 thousand astrocytes, in our slice (250 μm thick) we expect 3 thousand and in the half we imaged around 1.5 thousand. As explained above, pictures were taken in a single z-plane (1 μm), thus having between 5-10 astrocytes in the field of view is reasonable with this setup.

We have included a paragraph summarizing the technical limitations. Please, find the changes in the discussion (page 34 lines 728-734) of the revised manuscript.

**4.4)** Third, the heatmaps presented in Figure 6i to show Ca2+ wave propagation across the astrocytes are very weak evidence.  To be convincing, surely the wave should be shown to propagate in the same manner (at least through the same interconnected cells, not necessarily in the same order dependent on where the wave initiates) multiple times i.e. is this a stochastic process or reproducible?  To be more convinced of the initiation of intercellular calcium waves by GC treatment, some sort of correlation analysis should be performed.  However, it may be that the number of events in the slices are too few to answer this on a limited sample size or that SCN astrocytes don't produce these calcium waves in response to GC treatment.  Perhaps the authors could consider using a positive control treatment to reveal calcium waves in their preparation (e.g. mechanical stimulation, ATP or glutamate) so that the degree to which GC treatment induces this behaviour alongside a known initiator can be seen?  Re-analysis of the data using a less conservative threshold (as above) may improve the ability to detect correlated waves of activity.

**Response #14:**

We thank the reviewer for this comment. In the previous version of our manuscript, we used a heatmap to show that the Ca^2+^ propagation events induced by CORT, were blocked by the treatment with the inhibitor of astrocyte-astrocyte communication. We agree with the reviewer’s comment that although we observed these propagation events happening mostly with CORT, a proper quantification was necessary. To observe propagation events on the same slice our 200 s recordings are likely too short. Therefore, we have:

1) Analyzed the propagation of Ca^2+^ events through the astrocytic syncytia by detecting synchronous events from astrocyte-like ROIs. To this end, binary raster tables of individual astrocyte Ca^2+^ events were scanned in MATLAB using an expanding temporal window: an initial 3-second window identified timepoints when ≥2 astrocytes exhibited Ca^2+^ events; when additional active astrocytes were detected in a subsequent 3-second interval, the window expanded iteratively until no new participants were found. The “level” of the synchronous event (number of ROIs in sync) was defined as the total number of co-active ROIs within the expanded window and was assigned to the timepoint of the first detected event. This approach captures the full temporal extent and participation level of coordinated astrocytic Ca^2+^ episodes while preventing fragmentation of sustained synchronized events into multiple lower-level detections.

The code used for this analysis is deposited at: https://github.com/SoriaFN/GlioMatrixLab/blob/main/MATLAB/detect_multievents_v2.m

In the revised version of our manuscript we include a new figure 6l. All Ca^2+^ events from astrocyte-like ROIs were followed during the 200 s while perfusing VEH, CORT and CORT + GAP26 to the SCN slice. The heatmaps show a temporal sequence of activation with the color indicating the number of ROIs that fired in synchrony from a representative experiment. Fig 6m shows that from the 8 slices (experiments shown in Fig 6i,j), synchrony was detected in 7 of them. In 6 out of these 7 slices, CORT treatment was able to induce synchronous events. This synchrony seems to be generated by CORT and dependent on astrocyte-astrocyte communication, because in both, VEH and CORT + GAP26 treatments, we only observed synchronous events in 3 slices out of 7 (Fig 6m).

We have included the details of the analysis in the results (page 25 lines 535-537) and methods (page 51 lines 1087-1099).

**4.5)** Finally, given that the previous datasets show that a small number of neurons express the GR, have the authors explored their neuronal dataset in this context (some cells responding, some not responding for example)?  In other brain areas, calcium levels do not necessarily change immediately in neurons upon GC treatment (being delayed as transcriptional programmes control this) and their responses are complex (e.g. PMID23340218, PMID22153720, PMID39248182).  Could the authors discuss potential differences between the neuronal response versus the astrocytic response?

**Response #15:**

We thank the reviewer for this interesting comment on the puzzling effects of CORT inducing rapid Ca^2+^ events. During the revision process we expanded the literature revision and performed experiments to further characterize the observed effects. We have:

1) Introduced new references and discussed that while genomic effects of GCs are well described, and slow responses are expected, we here are observing fast responses in the range of minutes. In neurons, these rapid GC actions have been described in excitatory/inhibitory pre- and post-synapses, though activation of voltage-gated calcium and potassium channels or indirectly through the cannabinoid pathway (reviewed in PMID: 39004314). In astrocytes, these rapid actions are less well-described.

2) Performed **three new** experiments:

**2.1.** First, we used the same sequence of recordings in slices that were incubated before and during the recording in aCSF containing actinomycin D (a transcription inhibitor which intercalates into DNA, preventing the unwinding of the DNA double-helix and inhibiting the RNA polymerase activity) and cycloheximide (a translation inhibitor which interferes with the translocation step of translation elongation) for three main reasons:

2.1.1. To confirm that the rapid Ca^2+^ events induced by CORT in SCN astrocytes do not depend on transcriptional or post-transcriptional events.

2.1.2. To avoid having a mixture of CORT fast and slow actions. Since the whole sequence of recording can take more than 1 hour, in this experiment we confirm that slow actions are not part of the effect that we initially saw.

2.1.3. To repeat the experiments in more mice and obtain, independently of the technical limitations, confirmation that the effects we observed are reproducible.

Data are presented in Fig 6i and S10l

**2.2.** Second, we used the same sequence of recordings in slices obtained from the mouse at ZT12. For these experiments we also incubated the slices before and during the recording in aCSF containing actinomycin D and cycloheximide for three main reasons:

2.2.1. To align our data to previous findings from other labs showing that SCN astrocytes are more active during the dark phase (opposite phase as SCN neurons). This timepoint is physiologically more relevant because the SCN expects higher levels of GCs.

2.2.2. To align our functional data with the re-analysis shown in S9 Fig from previously published data sets. This analysis suggest that GR-related pathways are induced when physiological GC levels are (or expected to be) at their peak.

2.2.3. To further confirm that the effects we initially observed are reproducible.

Data are presented in Fig 6j and S10m

**2.3.** Third, we treated the slice with CORT-BSA (corticosterone conjugated with bovine serum albumin) to assess whether the rapid CORT effects were mediated by the action of the hormone on membrane-bound GR.

Data are presented in Fig 6k and S10n

In the revised version of our manuscript we have discussed, as the reviewer suggested, potential differences between neuronal and astrocytic responses to GCs. We have also introduced the three new experiments uncovering further mechanistic details on the CORT effect in the SCN. We could show that CORT induces rapid effects independent of transcription/translation, that the effect of CORT is stronger (i.e., leading to higher numbers of Ca^2+^ events/minute) at ZT12 in alignment with previously published data and that at both timepoint the effects are prevented by blocking the astrocyte-astrocyte communication. The third experiment suggests that the fast effects induced by CORT are mediated by membrane-bound GR because the effect is blocked by the GR antagonist RU486.

Of course, assessing cell-specific responses and the consequences of their interaction is already on our near future agenda. Please, find changes in the results (page 24 lines 502-515, page 25 lines 516-537) of the revised manuscript, Fig 6 and S10.

**5) Bioluminescence recordings**

**General response:**

During the revision, we have re-analyzed the data from the bioluminescence assay as suggested by reviewer#1. When data are analyzed this way, we still see changes in the same direction and we still find them to be statistically significant (please, see **response #16-20** below). The magnitude of the CORT effect, however, is markedly smaller with this analysis. An average phase delay of 2.12 ± 0.25 h (mean ± SEM) might not be biologically relevant. Statistical comparison revealed a significant period difference between pre- and post-treatment intervals. CORT induce a period lengthening compared to DMSO controls that might explain the reduction in the magnitude of the phase-shift with this new calculation strategy. We are reporting now an average phase delay of that might not be biologically relevant.

Due to a bottleneck in mouse supplies, we were unable to perform further bioluminescence experiments to increase the sample size. Moreover, during the period of revision, a paper was published showing that dexamethasone (an agonist of the GR) does not have an effect shifting the phase of the SCN clock in adults (PMID: 41314247).

Given these considerations, and in light of the strong and compelling molecular biology (and now the Ca^2+^ imaging) results, we would like to follow editors’ and reviewers’ advice regarding this data set. We have removed figure 7 and associated supplementary material from the revised version because we feel the data is not providing relevant evidence. As reviewer #2 already commented, the manuscript would remain impactful even if bioluminescence data is deemphasized or removed.

**5.1)** "Interestingly, the ascending phase of PER2::LUC expression typically occurred around the end of the subjective light phase and the beginning of the subjective dark phase…". It is well established that the peak (end of the ascending phase) of PER2 expression marks the transition from the light phase to the dark phase (e.g. PMID10719897, PMID11181971), am I missing something with the definition of the ascending phase?.
The authors describe the times of treatment as the "ascending phase" or the "descending phase", as evidenced above, this is ambiguous.  Precise treatment phases should be calculatable from the PER2::LUC oscillation.  It is well established in phase-response curves that there are "dead zones" where no shift occurs within both the ascending and descending intervals - are non-effective treatments hitting these phases?  Treatments should be compared using the appropriate model via comparisons within restricted within the same treatment time-windows.

**Response #16:**

We thank the reviewer for this relevant comment. We agree that the use of the terms “ascending” and “descending” phases may be ambiguous. The timing of our treatments were chosen to coincide with known phase-sensitive windows of the circadian cycle: in average 4.6 ± 0.35 h before the PER2::LUC peak (ascending phase) and in average 5.8 ± 1.01 h before the trough (descending phase), consistent with our own experience and previous studies that have used similar timings to induce phase shifts with diverse stimuli such as PACAP, glutamate, forskolin, and temperature pulses (e.g., PMID: 24656826, PMID: 30984034, PMID: 30710088, PMID: 30287844, PMID: 18487196, PMID: 20947768). In Fig 8R we compared all treatment times at the ascending and descending phase of PER2::LUC expression, to ensure that treatments were delivered at comparable phases.

**Fig 8R:** on the left: Treatment in the ascending phase of PER2::LUC, average of 4.6 h before the expression peak. On the right: Treatment in the descending phase of PER2::LUC, average of 5.8 h before the trough.

**5.2)** It is unclear how the phase-shift was calculated. In the methods, it is described as "the time when the fitted curve and the post-treatment curve crossed the zero-line of the first time after treatment" while in Figure 7a, it is indicated by an arrow pointing out the difference between the peaks of the PER2::LUC signal and the fitted curve.  In fact, looking at the first point that the two plotted data cross the zero-line, it looks like there is no phase-shift in the GC treated slice. It is also unclear how the CORT was administered to the slice - is this a transient droplet treatment to the slice representing an acute spike or pulsatile GR activation, or addition to the medium representing a chronic GR activation?  These two different administration methods represent two very different contexts for GR activation.

**Response #17:**

We thank the reviewer for this comment and the opportunity to clarify these two aspects:

1) In **response #18** (below) we show the results of the re-analysis of the phase-shift as the reviewer suggested.

2) We apologize for the lack of detail in the methods. In our experiments, all treatments were administered via addition to the culture medium, resulting in a sustained elevation of the concentration rather than a brief spike.

**5.3)** What are the error values associated with the fits used for the cosinor analysis?  The example fit in Figure 7a seems poor and the waveform appears to change shape as the recordings continues - becoming more asymmetrical. It is hard to tell if this is a consequence of GC treatment or something to do with the slice health.  Presenting other exemplar traces here would help to clear up this data set, as would the provision of additional traces for the other treatment and time conditions.  The use of two-cycles in the baseline to fit the projected curve seems quite low and may not give a robust period estimate. Is there a period change post-treatment that may need to be factored into the analysis?  If not, could the post-treatment interval be used to generate a more accurate curve that can be projected backwards from the treatment interval to the baseline interval?

**Response #18:**

We thank the reviewer for these helpful suggestions. We agree that the trace shown in Figure 7a is noisy and less symmetrical after treatment and that using only two pre-treatment cycles to estimate the baseline period may limit precision. Therefore, we have calculated the phase-shift as the reviewer suggested to minimize the influence of noise and cycle-to-cycle variability in the raw PER2::LUC signal. Then, phase was estimated using fitted oscillatory curves generated in GraphPad Prism rather than directly from raw bioluminescence traces. A forward fit was generated from two pre-treatment cycles and extrapolated beyond the treatment time point to represent the expected phase progression in the absence of treatment. In parallel, a backward fit was generated from the first two post-treatment cycles and extrapolated toward the treatment time point to represent the observed phase following perturbation.

In a first step, we re-analyzed the dataset using a corrected time window for goodness-of-fit estimation. Specifically, R² was calculated using two cycles prior to treatment and two cycles immediately following treatment, excluding the immediate treatment interval to avoid transient distortions and ensure comparability across conditions. All slices included in the analysis (i.e., all treatments at ascending and descending phase of PER2) showed robust and well-defined oscillations (pre-treatment R² > 80), indicating consistent tissue viability across conditions and small changes in ΔR², indicating preserved rhythm quality after treatment (Fig 9R).

**Fig 9R**

**Fig 9R:** Goodness-of-fit estimation for forward fit (before treatment) and backward fit (after treatment). R² was calculated excluding the immediate treatment interval to avoid transient distortions and ensure comparability across conditions. No significant differences were observed.

Together, these data indicate that the effects observed in phase-shift cannot be attributed to weakened rhythmicity or reduced tissue quality.

In a second step, phase shifts were quantified as the time difference between the first corresponding ascending zero crossing after the treatment time point in the two fitted curves (forward fit in the color of the treatment and backward fit in blue, Fig 10Ra-e). Positive values indicate a phase advance, whereas negative values indicate a phase delay. Period (Fig 10Rf) and amplitude (Fig 10Rg) were determined from the same two cycles before and after treatment.

With this analysis, we still see that slices treated at the ascending phase of PER2::LUC with CORT 1 µM showed a phase delay of PER2::LUC expression compared to DMSO controls (Fig 10Rb,e). This effect was prevented by specifically blocking astrocyte communication with GAP26 (Fig 10Rc,e). Compared to other specific Cn43 blockers, GAP26 alone had no effect on the period but significantly reduced the amplitude of PER2::LUC rhythms (Fig 10Rf,g). In contrast, CORT treatment induced no phase shift when slices were treated at the descending phase of PER2::LUC indicating a time dependent effect of CORT.

Statistical comparison revealed a significant period difference between pre- and post-treatment intervals. CORT induce a period lengthening compared to DMSO controls that might explain the reduction in the magnitude of the phase-shift with this new calculation strategy. We are reporting now an average phase delay of 2.12 ± 0.25 h (mean ± SEM) that might not be biologically relevant.

**Fig 10R:**

CORT

CORT + GAP26

a

b

GAP26

c

d

DMSO

e

f

g

**Fig 10R: SCN respond to GCs inducing a phase shift of the molecular clock. a-d)** Representative luminescence recordings of SCN slices collected from PER2::LUC mice (at PND 30-45) maintained in culture medium containing luciferin. Slices treated at the ascending phase of the third peak of PER2::LUC with CORT (1 µM, n=8), CORT + the inhibitor of Connexin 43, GAP26 (400 µM, n=7), the combination of the two and DMSO as vehicle control. In black the raw data, in the colors of the treatments the forward fit generated from two pre-treatment cycles and extrapolated and in blue the backward fit generated from the first two post-treatment cycles and extrapolated towards the treatment time point to represent the observed phase following perturbation. **e)** Phase shift was quantified as the time difference between the first corresponding ascending zero crossing after the treatment time point in the two fitted curves. Positive values indicate a phase advance, whereas negative values indicate a phase delay. Data are expressed as mean ± SEM and analyzed by 2-way ANOVA after testing normality by Shapiro-Wilk test. We found a significant effect of the treatment F(3,40)=5.32, p=0.0035, of the time of treatment F(1,40)=8.927, p=0.005 and a significant interaction between the two factors F(3,40)=3.303, p=0.029. Holm-Sidak’s multi comparison test detected a significant effect of the PER2::LUC phase only for the CORT treatment (CORT ascending vs CORT descending, **p=0.0031) and differences between treatments only at the ascending phase of PER2 (DMSO vs CORT, ***p=0.0004; CORT vs CORT + GAP26, **p=0.0086 and CORT vs GAP26, **p=0.0035. **f)** Period changes before and after the treatment are expressed as mean ± SEM and analyzed by 2-way ANOVA after testing normality by Shapiro-Wilk test. We found a significant effect of the treatment (F(3,39)=3.55, p=0.0228, of the time of treatment F(1,39)=7.130, p=0.011, no significant interaction between the two factors was found. Holm-Sidak’s multi comparison test detected a significant change of the PER2::LUC period length for DMSO vs CORT at the ascending phase, *p=0.0179. **g)** Amplitude changes after the treatment are expressed as mean ± SEM and analyzed by 2-way ANOVA after testing normality by Shapiro-Wilk test. We found a significant effect of the treatment (F(3,40)=16.41, p<0.0001, no effect of the time of treatment and no significant interaction between the two factors was found. Holm-Sidak’s multi comparison test detected a significant effect of the PER2::LUC amplitude only for the DMSO ascending vs GAP26 ascending, ***p<0.0001, CORT ascending vs GAP26 ascending, ***p=0.0002 and CORT descending vs GAP26 descending, *p=0.0264.

**5.4)** The data in Figure 7c should be analysed as a two-way ANOVA where treatment, phase and the interaction between the two are tested.

**Response #19:**

We thank the reviewer for this suggestion. A two-way ANOVA is the correct statistical test to apply in this case. Please, see Fig 9R above, the figure legend includes the results of the statistical test.

**5.5)** Finally, although on the face of it the pharmacological data is compelling, the authors cannot discount the response of neurons to the GC stimulation in driving the phase change in these slices.  The blockade of the response by GAP26 is evidence of astrocytic involvement, but does not prove that the GC stimulation is conducted via the astrocytes - what if blocking astrocytic communication via gap junctions prevents the neurons from generation a phase-shift regardless of the stimulus due to their influence on the network?  Are comparable results seen with slices from younger mice where (according to the data) the response of astrocytes to GCs would be lesser?  Are these responses still blocked by GAP26, representing the influence of astrocytes over neurons rather than their direct effects? The potential for astrocytic modulation of the neuronal response should be discussed and if possible explored.

**Response #20:**

We thank the reviewer for raising this important point. We agree that the fact that GAP26 blocks the effect of CORT only proves astrocytic involvement, but we cannot discount the response of neurons with the data we have. Therefore, in the revised version of the manuscript we have:

- Acknowledged the possibility that SCN neurons, microglia and oligodendrocytes might also receive and respond to peripheral GCs.

- Unfortunately, due to a bottleneck in mouse supplies, we were unable to perform further bioluminescence experiments in younger mice.

**Reviewer #1: minor points:**

**6)** The presentation of false-colored and merged images are difficult to assess. The images would be easier to digest if presented as split greyscale alongside merged images.

**Response #21:**

We thank the reviewer for this comment. We have done so in Fig 1, 3, 4 and 5 and also in Fig S5 and S9 (now Fig S6 and S10, respectively). We agree with the reviewer, it helps the visualization.

**7)** The in-situ hybridisation images are hard to assess, especially without the nuclear counterstain.  This should be provided so that cellular colocalization can be better visualised.

**Response #22:**

We thank the reviewer for this comment. We have done so in Fig 3 and again we agree with the reviewer, it helps the visualization.

**8)** What is the rationale for the choice of doses for the pharmacological agents administered?

**Response #23:**

We thank the reviewer for this comment. All the doses of the treatments we performed were chosen based on preliminary data (published and/or unpublished) from our laboratories and by comparison of doses used by others.

- The CORT dose given to the mice either prenatally or to assess the activation of GR in the SCN is the same (5 mg/kg b.w. in PEG-400 50% in PBS) and we have used it before (Astiz et al., 2020 Nat Comms). It could be translated to a pharmacological dose in humans (e.g. pregnant woman at risk of premature delivery). Please, find a comment in the methods (page 36, lines 761-764) of the revised manuscript.

- The CORT dose used in *in vitro* experiments (1 μM) has been chosen to mimic the *in vivo* plasma concentration between circadian peak (≈ 200-300 ng/mL) and the concentration reached after pharmacologic treatment (≈ 700-800 ng/mL). In addition, the decision on the concentration used is based on *in vitro* experiments in primary hypothalamic astrocytes in culture in which we have performed a dose-response curve assessing the up- or down-regulation of GR target genes. This concentration is also widely used by other authors (recently reviewed in PMID: 39004314).

- The GAP26 dose used in *in vitro* experiments (400 μM) has been used in similar experiments by some of the co-authors of this work (PMID: 38326974)

- Concentrations of other drugs (e.g. RU486, CORT-BSA) were selected according to the previous literature (recently reviewed in PMID: 39004314).

We have included the missing references on the list.

**9)** Figure 1f is not referenced in the text.

**Response #24:**

We thank the reviewer for this comment. Now Fig 1f is referenced in the revised version of our manuscript.

**10)** Figure 3e, the inset region chosen to highlight astrocytic expression of GR sits outside the SCN and mostly represents extra-SCN signal.

**Response #25:**

We thank the reviewer for this comment. In the revised version of the manuscript we are providing 2 zoomed-in images that are located within the SCN (Fig 3d-f).

**11)** In the figure legend for Figure 7c, CORT+GAP26 versus GAP26 gives a significant result on the Kruskal-Wallis test.  This should be marked on the plot.

**Response #26:**

According to a comment above, we now analyzed the data by 2-way ANOVA, please, see Fig 10R above.

**12)** In Supplementary Figure 1, scale bars are missing from the images.

**Response #27**

We thank the reviewer for this comment. We have added the scale bars to the images in S1 Fig (now S2 Fig).

**13)** Supplementary Figure 5, the inset regions presented should be highlighted on the full-field images.

**Response #28**

We thank the reviewer for this comment. We have highlighted the inset regions in the full-field image. In the revised version of our manuscript, this is S6 Fig now.

**14)** Supplementary Figure 8, the reanalysis of the publicly available datasets from Wen et al, 2020 and Morris et al, 2021 are nice, but I believe there is a misunderstanding regarding the Morris et al, 2021 dataset.  Unless I am mistaken, this was performed on free-running SCN organotypic explants, not harvested from animals acutely.  If this is the case, the interpretation of this data and detailing in the results (starting at line 398, page 19) and in the methods (starting at line 922, page 42) needs rewriting. If the analysis holds in the Morris et al, 2021 dataset, it means that even in the absence of circulating GCs the SCN still expects to receive the signal - it is a feature of the daily programme of the SCN.

**Response #29**

We thank the reviewer for this comment. We apologize for the mistake and very much appreciate the reviewer’s perspective. Indeed, the comparison of the scores holds even when GCs concentrations do not change, suggesting that the SCN astrocytes can anticipate and likely gate the response to GCs. We have rephrased the sentence in the results and in the methods to reflect this difference between both data sets.

Please, find the changes in the results (page 20 lines 427-428; page 21 lines 429-449; page 25 lines 524-527) and methods (page 47 lines 1004-1016) of the revised manuscript.

**Reviewer #2:** This manuscript by Handler, Sreenivasan, Pilorez et al. addresses an important and longstanding question in circadian neuroscience: whether the adult SCN retains sensitivity to glucocorticoids. Prior evidence indicates that the glucocorticoid receptor (GR) is expressed prenatally in the SCN but absent in the adult SCN, though this issue has not been fully resolved. The authors show that prenatal exposure to glucocorticoids at the "wrong" circadian phase alters re-entrainment dynamics to jet lag later in adulthood. They report reduced GR expression in SCN astrocytes in these "out-of-phase" animals. Through snRNA-seq, they demonstrate a developmental shift in Gr expression from neurons to astrocytes. Using PLA and triple-label IHC, they further show that GR activation in astrocytes varies across circadian time and in response to CORT injection. Finally, they present physiological evidence: CORT-evoked astrocytic calcium activity (sensitive to gap-junction blockade) and phase-shifting of PER2::LUC rhythms. Overall, the manuscript presents compelling molecular evidence that SCN astrocytes remain glucocorticoid-responsive in adulthood, though I find the physiological experiments less convincing and in need of further rigor.

**1)** Astrocyte identification criteria: the calcium imaging experiments rely on complex morphological and temporal parameters to classify Ca²⁺ events as astrocytic. While the cited references provide partial justification, I did not find a clear precedent for the specific "astrocyte isolation profile" the authors employ. As noted by the authors themselves (Line 445), these parameters may under-detect certain events, and the choice of thresholds seems somewhat arbitrary.

**Response #30:**

We thank the reviewer for this comment (shared with reviewer #1, response #13) and we agree that with this conservative threshold, we are describing events in the soma and the main processes. Therefore, considering the reviewers’ comments we have re-focused the discussion on the effect of GCs on the SCN, explaining the technical limitations of our approach. Additionally, we have:

1) Generated data to support the reasoning behind considering only events longer than 10 s. We run 3 experiments in which we incubated the acute slices with sulforhodamine 101 (SR101), a dye that preferentially labels astrocytes (PMID: 35199029). In this way we were able to identify events from astrocytes with more specificity and assess the length of the Ca^2+^ events in SR101-positive cells. As shown in Fig 7R, the astrocyte-like ROIs show Fluo-4 signal in the soma and the main processes, and we can confirm that our threshold of 10 sec detects almost 90% of the events in SR-101-positive cells.

**Fig 7R:**

**Fig 7R**: Astrocyte event identification: Left: Representative picture of an active (i.e.: high Fluo-4 signal in green) astrocyte that is labelled with Sulforhodamine 101 (1 μM in ACSF, S7635 Sigma Aldrich), scale bar 2 μm. Right: Dispersion of the length of the Ca^2+^ events in sulforhodamine + astrocytes, almost 90% of the astrocytes show events longer than 10s.

2) Explained our data in the context of the limitations of the experimental approach that does not allow us to describe the astrocytic physiology in full or to detect more astrocytes in the field of view. The main limitations are:

- **Ca^2+^ sensor**: We are aware that using genetically encoded Ca^2+^ sensors could have provided an increased sensitivity to detect events and specificity restricting its expression to astrocytes. However, for this set of experiments acute slices were prepared from wild-type mice of 20-30 days of age in LD12:12 conditions to match the transcriptional data. The introduction of a calcium sensor like GCaMP could have been done using a viral approach (i.e., introducing it with an AAV vector during early postnatal life) or using a mouse line (i.e., GFAP-GCaMP), in both approaches the chelating activity of the sensor could have had an impact on the development of the SCN. Therefore, we decided to use a cell permeable sensor. On the one hand, Fluo4-AM has several advantages such as: a high signal-noise ratio, low concentration requirement during the slice incubation and an excitation wavelength well-suited for our laser. On the other hand, Fluo4 has several limitations such as: it only detects events in the soma and in the large processes, it is not specific for astrocytes (although it tends to label a larger proportion of astrocytes compared to other cell types, see PMID: 22027012), it does not penetrate more than 60-70 μm from the surface of the slice.

- **Microscope:** We aimed at scanning the largest xy area possible of the SCN slice because the total number of astrocytes is low and we wanted to capture the global effects of CORT. Therefore, with our setup, 3D scanning was not possible because this would have increased significantly the total time to acquire a field of view, reducing even more the capacity to detect fast events. Thus, images were acquired at a speed of 1 Hz, events faster than 1 second could not be detected. Furthermore, without the use of a high-speed camera (PMID: 39804104), most neuronal fast events are lost due to the slow nature of the non-resonant scanner of our setup.

- **Expected number of astrocytes in the field of view:** We made a rough estimation of the number of astrocytes that we should see, and we cannot expect more than 5-10 astrocytes. We reasoned that the whole SCN has around 6 thousand astrocytes, in our slice (250 μm thick) we expect 3 thousand and in the half we imaged around 1.5 thousand. As explained above, pictures were taken in a single z-plane (1 μm), thus having between 5-10 astrocytes in the field of view is reasonable with this setup.

We have included a paragraph summarizing the technical limitations. Please, find the changes in the discussion (page 34 lines 728-734) of the revised manuscript.

**2)** Risk of neuronal contamination: the assertion (Line 447) that events longer than 10 s can be assumed to exclude neuronal activity is problematic. Calcium indicators are inherently slow, and Fluo-4 in particular is ill-suited to exclude the possibility that brief neuronal bursts could generate long-tailed transients. Thus, the >10 s threshold cannot guarantee clean separation between astrocytic and neuronal signals.

**Response #31:**

We thank the reviewer for this comment. We agree that our experimental approach is restrictive to events in the soma and the main processes. Therefore, considering the reviewers’ helpful comments we have re-focused the discussion on the effect of GCs on the SCN. In the revised version we have:

1) Described with further mechanistic detail the effects of GCs in the SCN (please, see below).

2) Acknowledged the possibility that other SCN cells might also receive and respond to peripheral GCs. Please, find changes in the discussion (page 34 lines 726-728) of the revised manuscript.

3) Explained the data in the context of the limitations of our experimental approach. Please, find changes in the discussion (page 34 lines 728-734) of the revised manuscript.

**3)** Statistical considerations: the number of recorded astrocytes is low, and it is not clear what constitutes the true statistical unit. A nested approach accounting for both cells and slices (animals) would strengthen the analysis. It would also be helpful to provide the exact number of contributing SCN slices for Fig. 6.

**Response #32:**

We thank the reviewer for this comment. We have always prepared 1 slice per animal, the number of slices (animals) from each experiment is indicated in the methods section, in the figures and in the figure legends now. We apologize for the confusion.

We thank the reviewer for the analysis suggestion. To apply a nested approach, we had to assess Ca^2+^ events differently as before. In the experiments done for the revision of the paper (Fig 6i-m and S10l-n) we have included all the ROIs that showed at least one event at any of the treatments. Although data are analyzed differently, we obtained the same result.

In this new set of experiments, we have used a negative binomial generalized linear model to assess statistical differences between treatments. This analysis accounts for ROIs nested within slices (animals) with a countable variable (number of Ca^2+^ events). In this way we can assess:

- slice-to-slice correlation avoiding pseudo replication (i.e., multiple ROIs from the same slice are not independent observations)

While controlling for

- overdispersion caused by including all ROIs that showed at least one event as well as experimental factors that contribute to variance between slices (animals) such as: dye loading efficiency, z-plane imaged, slice selection, slice preparation time.

The script used for this analysis is openly available at https://doi.org/10.5281/zenodo.19188776.

**4)** Calcium wave quantification: the claim of coordinated astrocytic calcium activation across ROIs (e.g., Fig. 6I) is not well supported by the heatmap. This should be quantified rather than described qualitatively.

**Response #33:**

We thank the reviewer for this comment (shared with reviewer #1, response #14). In the previous version of our manuscript, we used a heatmap to show that the Ca^2+^ propagation events induced by CORT, were blocked by the treatment with the inhibitor of astrocyte-astrocyte communication. We agree with the reviewer’s comment that although we observed these propagation events happening mostly with CORT, a proper quantification was necessary. To observe propagation events on the same slice our 200 s recordings are likely too short. Therefore, we have:

1) Analyzed the propagation of Ca^2+^ events through the astrocytic syncytia by detecting synchronous events from astrocyte-like ROIs. To this end, binary raster tables of individual astrocyte Ca^2+^ events were scanned in MATLAB using an expanding temporal window: an initial 3-second window identified timepoints when ≥2 astrocytes exhibited Ca^2+^ events; when additional active astrocytes were detected in a subsequent 3-second interval, the window expanded iteratively until no new participants were found. The “level” of the synchronous event (number of ROIs in sync) was defined as the total number of co-active ROIs within the expanded window and was assigned to the timepoint of the first detected event. This approach captures the full temporal extent and participation level of coordinated astrocytic Ca^2+^ episodes while preventing fragmentation of sustained synchronized events into multiple lower-level detections.

The code used for this analysis is deposited at: https://github.com/SoriaFN/GlioMatrixLab/blob/main/MATLAB/detect_multievents_v2.m

In the revised version of our manuscript we include a new figure 6l. All Ca^2+^ events from astrocyte-like ROIs were followed during the 200 s while perfusing VEH, CORT and CORT + GAP26 to the SCN slice. The heatmaps show a temporal sequence of activation with the color indicating the number of ROIs that fired in synchrony from a representative experiment. Fig 6m shows that from the 8 slices (experiments shown in Fig 6i,j), synchrony was detected in 7 of them. In 6 out of these 7 slices, CORT treatment was able to induce synchronous events. This synchrony seems to be generated by CORT and dependent on astrocyte-astrocyte communication, because in both, VEH and CORT + GAP26 treatments, we only observed synchronous events in 3 slices out of 7 (Fig 6m).

We have included the details of the analysis in the results (page 25 lines 535-537) and methods (page 51 lines 1087-1099).

**5)** Circadian timing: recordings were performed only at ZT 0-2. Given the well-established circadian modulation of both SCN physiology and glucocorticoid levels, repeating these experiments at additional circadian phases would provide much stronger support for the conclusions.

**Response #34:**

We thank the reviewer for this comment that encourage us to perform more experiments and explore further mechanistic details of the effects of CORT in the SCN.

For the revision of our manuscript we decided to perform **three new** experiments. Although experiment #2 is the one that the reviewer is specifically asking for, we think that the results of the other two are worth mentioning.

**1.** First, we used the same sequence of recordings in slices that were incubated before and during the recording in aCSF containing actinomycin D (a transcription inhibitor which intercalates into DNA, preventing the unwinding of the DNA double-helix and inhibiting the RNA polymerase activity) and cycloheximide (a translation inhibitor which interferes with the translocation step of translation elongation) for three main reasons:

1.1. To confirm that the rapid Ca^2+^ events induced by CORT in SCN astrocytes do not depend on transcriptional or post-transcriptional events.

1.2. To avoid having a mixture of CORT fast and slow actions. Since the whole sequence of recording can take more than 1 hour, in this experiment we confirm that slow actions are not part of the effect that we initially saw.

1.3. To repeat the experiments in more mice and obtain, independently of the technical limitations, confirmation that the effects we observed are reproducible.

Data are presented in Fig 6i and S10l

**2.** Second, we used the same sequence of recordings in slices obtained from the mouse at ZT12. For these experiments we also incubated the slices before and during the recording in aCSF containing actinomycin D and cycloheximide for three main reasons:

2.1. To align our data to previous findings from other labs showing that SCN astrocytes are more active during the dark phase (opposite phase as SCN neurons). This timepoint is physiologically more relevant because the SCN expects higher levels of GCs.

2.2. To align our functional data with the re-analysis shown in S9 Fig from previously published data sets. This analysis suggest that GR-related pathways are induced when physiological GC levels are (or expected to be) at their peak.

2.3. To further confirm that the effects we initially observed are reproducible.

Data are presented in Fig 6j and S10m

**3.** Third, we treated the slice with CORT-BSA (corticosterone conjugated with bovine serum albumin) to assess whether the rapid CORT effects were mediated by the action of the hormone on membrane-bound GR.

Data are presented in Fig 6k and S10n

In the revised version of our manuscript we have introduced the three new experiments uncovering further mechanistic details on the CORT effect in the SCN. We could show that CORT induces rapid effects independent of transcription/translation, that the effect of CORT is stronger (i.e., leading to higher numbers of Ca^2+^ events/minute) at ZT12 in alignment with previously published data and that at both timepoint the effects are prevented by blocking the astrocyte-astrocyte communication. The third experiment suggests that the fast effects induced by CORT are mediated by membrane-bound GR because the effect is blocked by the GR antagonist RU486.

We believe that this data provides a stronger support to our conclusions.

Please, find changes in the results (page 24 lines 502-515, page 25 lines 516-537) of the revised manuscript, Fig 6 and S10.

**6)** Event metrics: the chosen metrics (frequency, ΔF/F) are acceptable but incomplete. Considering the unusually long transients, integrated calcium (area under the curve) would be more meaningful, as it distinguishes brief events from prolonged ones.

**Response #35:**

We thank the reviewer for this comment, and we agree that the area under the curve provides an integrated way of measuring the effects that accounts for: number of events, length and amplitude. We have calculated the AUC for all the experiments (previous and new ones) and found a similar tendency than with the number of events/min although not enough to reach statistical significance (Fig 6g and S10l-n). All the experiments consistently showed that CORT increases the frequency of the Ca^2+^ events.

Please, find changes in page 23, lines 490-492 of the revised manuscript.

**7)** Methodological alternatives: while the use of Fluo-4 with morphological/kinetic criteria is creative, a more definitive approach would be astrocyte-specific GECI expression (e.g., AAV-astrocyte promoter-Cre with Cre-dependent GCaMP). Even accounting for potential drawbacks of promoter selectivity and infection efficiency, such an approach would yield clearer and higher-throughput data than the current method.

**Response #36:**

We thank the reviewer for this comment. We are aware that using a genetically coded Ca^2+^ sensors could have provided an increased sensitivity to detect events and specificity by restricting its expression to astrocytes. However, for this set of experiments acute slices were prepared from wild-type mice of 20-30 days of age in LD12:12 conditions to match the transcriptional data. The introduction of a calcium sensor like GCaMP could have been done using a viral approach (i.e.: introducing it with an AAVs during early postnatal life) or using a mouse line (i.e.: GFAP-GCaMP), in both approaches the chelating activity of the sensor could have an impact the development of the SCN. Therefore, we decided to use a cell-permeable sensor like Fluo-4.

We are currently working on improving our experimental approach by using an inducible genetically encoded sensor that can allow us to overcome these technical difficulties and explore aspects of astrocytic physiology with more detail.

This technical limitation is now better explained in the discussion (page 34 lines728-734) of the revised manuscript.

**8)** PER2::LUC phase-shifting: the finding that CORT can delay PER2::LUC rhythms around subjective dusk is intriguing but requires more discussion in the context of prior reports that the SCN is largely insensitive to dexamethasone-induced phase shifts (Balsalobre et al., 2000; van der Veen et al., 2012; Ota et al., 2020). Reconciling these findings would strengthen the manuscript's impact.

**Response #37:**

We thank the reviewer for this comment. In the discussion section of our revised manuscript, we have improved the contextualization of our findings. Please, find the changes in discussion (page 33 lines 706-711) of the revised manuscript.

During the revision, we have re-analyzed the data from the bioluminescence assay as suggested by reviewer#1. When data are analyzed this way, we still see changes in the same direction and we still find them to be statistically significant (please, see **response #16-20**). The magnitude of the CORT effect, however, is markedly smaller with this analysis. An average phase delay of 2.12 ± 0.25 h (mean ± SEM) might not be biologically relevant.

Due to a bottleneck in mouse supplies, we were unable to perform further bioluminescence experiments to increase the sample size. Moreover, during the period of revision, a paper was published showing that dexamethasone (an agonist of the GR) does not have an effect shifting the phase of the SCN clock in adults (PMID: 41314247).

Given these considerations, and in light of the strong and compelling molecular biology (and now the Ca^2+^ imaging) results, we would like to follow editors’ and reviewers’ advice regarding this data set. We have removed figure 7 and associated supplementary material from the revised version because we feel the data is not providing relevant evidence. As reviewer #2 already commented, the manuscript would remain impactful even if bioluminescence data is deemphasized or removed.

**9)** My overall assessment is that the molecular biology results are strong and compelling, particularly the evidence for GR expression and activation in SCN astrocytes. By contrast, the physiological experiments (Ca²⁺ imaging and slice bioluminescence) are less robust and, in their current form, somewhat detract from the otherwise elegant story. The manuscript would remain impactful even if these physiological data were deemphasized or removed. If retained, the experiments require additional rigor and clarification as noted above.

**Response #38:**

We thank the reviewer very much for the constructive criticism. We completely agree that further experiments and data analysis were necessary to back up our claims and that the manuscript has benefited from a sharper focus on our main findings.

As commented above, we believe that the manuscript would remain impactful even if bioluminescence data is deemphasized or removed.

**Reviewer #3: General impression:**

This is an interesting study addressing a topic of high translational potential. The manuscript presents an apparently solid set of data, but the way the experiments are introduced and have the results presented feels naïve and unsupervised.
The results are convincing, and the translational implications are touched upon, but the discussion ignores critical evidence from the literature.

**Response #39:**

We thank the reviewer for the careful and thoughtful assessment of our work. We have substantially re-structured, re-focused and provided additional experimental data strengthening our conclusions according to all three reviewers’ suggestions. We apologize for the missing references, ignoring the work of other laboratories was never our intention. We have included all the suggested references. We hope that this reviewer shares our enthusiasm about the revised version of our manuscript.

**1)** Introduction: Lines 65-70: The statement "One of the main hormonal outputs of the SCN are the "wakening" cues, glucocorticoids (GCs)…" is scientifically wrong. Please rephrase the sentence and split it into several clauses for clarity. Similarly, the last sentence of the paragraph is too vague and the wording is misleading: "Unlike other neuroendocrine systems, it is still unclear whether GCs target GR in the SCN and the influence on the circuit."

**Response #40:**

We thank the reviewer for this comment. We have rephrased those sentences to improve clarity and scientific accuracy in the revised version of our manuscript. Please, find changes in the page 4 lines 65-76.

**2)** Reference 12 is cited wrong repeatedly bundled with references 10 and 11. While the latter do refer to effects of GCs on SCN during development, the experiments described in reference 12 refer to coupling between SCN and peripheral oscillators, albeit in mice exposed to GCs during early developmental stages.

**Response #41:**

We thank the reviewer for this comment and for the careful revision of our manuscript at this stage. We apologize for the mistake with this reference, in fact, we exchanged it for the right reference from the same group and placed the reference later in the introduction (now [37]).

**3)** The reference used for estimation of astrocyte:neuron ratio is rather old (1983). Are there more recent studies estimating the density of astrocytes in the SCN? How does this ratio tally with the authors' observations of astrocytes in the SCN?
Lines 112-113: please rephrase the following sentence for clarity: "Unlike in neurons, GR remains expressed in astrocytes also in the adult circuit."

**Response #42:**

We thank the reviewer for this comment. We would like to keep this reference because although it is old, it is the first to report the proportion of neurons and astrocytes with an extremely careful anatomical assessment. As suggested by the reviewer, we have added other two recent references that also report this ratio in adult SCN using modern technical approaches. Moreover, in the new Fig 3b we are also showing that this proportion is reached at PND30.

**4)** Experimental model: Control animals, denoted as "naïve", are not exposed to experimental procedures ("sham"), therefore the outcome can be the effect of combined manipulation (i.e. stress) and administration of CORT (as acknowledged in the Results, lines 142-143). Not having the controls exposed to sham procedures undermines the statement of translational relevance of the results (line 666).

**Response #43:**

We thank the reviewer for this important point (shared with reviewer #1, response #2). The two control groups suggested (injected with saline s.c./manipulated at the in-phase or out-of-phase timepoints) would have allowed us to dissect the effect of the manipulation from the effects of the CORT. However, with these experiments we aimed at assessing the long-term effects of the exact same manipulation at different times of day during the critical developmental window. The naïve group was generated with the only intention of comparing the response to jetlag/expression of the receptor to an undisturbed condition. In the revised version of our manuscript we have:

1) Checked that throughout the text we do not use any reference to a “control” group when referring to the naïve group and explained in a clearer way the reasons for comparing the prenatal manipulation to such a group. Please, find a comment in page 36 lines 768-770 of the revised manuscript.

2) Inspired by the reviewers’ comment, we have re-analyzed the data from Fig 4 (b and e) to directly compare the plasma corticosterone levels in naïve mice and in mice injected with vehicle (VEH) and corticosterone (CORT) at the same time of day (Fig 1R).

**Fig 1R: Level of corticosterone in naïve mice and in mice injected with VEH or CORT.** Plasma corticosterone levels in mice at ZT0 (grey), at ZT0 1 hr after being injected with VEH (red) and at ZT0 1 hr after being injected with CORT (dark red). Corticosterone measurements were done in duplicates using an ELISA kit and following manufacturers’ instructions (Enzo, Catalogue number ADI-900-097, USA). Data are expressed as mean ± SEM, normality was confirmed by Shapiro-Wilk test and analyzed by 1-way ANOVA. Significant differences were found F (2,15)=93.91, p<0.0001. Tukey’s multi-comparison test showed significant differences for ZT0 vs ZT0 CORT (****p<0.0001) and ZT0 VEH vs ZT0 CORT (****p<0.0001).

Statistical analysis shows that at ZT0 the naïve and the VEH-injected mice show comparable levels of corticosterone in plasma, suggesting that the VEH treatment does not result in a significant elevation of corticosterone levels. Although these data are informative, as the reviewer points out, we cannot exclude effects of other factors besides CORT.

3) Explored, as the reviewers suggested, the possibility that the injection/manipulation could cause changes beyond corticosterone such as disruption of sleep/wake cycles. We have always assumed that the effect of the CORT injection was probably not too impactful on the maternal sleep/wake cycles because the injections were done at ZT0 and ZT12, in both cases during at the transitions between the sleep and wake phases. However, since we never tested this before, we decided to perform an experiment to assess the effects of the injection/manipulation on the activity 6 h after. We used bouts of activity detected with infrared motion detectors as a sensitive readout for “other factors” that would disrupt sleep/wake phases. Please, note that this detection system is “noisier” than the running-wheel data.

**Fig 2R:**

**Fig 2R: Activity bouts before and after injection/manipulation at ZT0 and ZT12 compared to naïve mice.** We assessed the activity of 7 female mice kept in LD 12:12 and left undisturbed (naïve), injected with VEH (PEG-400 50% in PBS) or injected with CORT (5 mg/Kg b.w in PEG-400 50% in PBS) at ZT0 and ZT12. On top we showed the bouts of activity recorded over 4 days, the arrow indicates the injection time with VEH or CORT and the dotted area depicts the injection day. That day is zoomed at the bottom. In the bottom center we show the average of activity bouts the first 6 hours after the injection with VEH or CORT at ZT0 or at ZT12 in comparison to a naïve group. Data are expressed as mean ± SEM, normality was confirmed by Shapiro-Wilk test and analyzed by 2-way ANOVA using treatment (None, VEH or CORT) and time of day (ZT0 and ZT12) as factors. As expected, significant differences were found for the time of day F(1,36)=145.1, p<0.0001 but the treatments did not change significantly the activity at any of the time points. The activity did not show any difference days after the manipulation.

These new data provide evidence that the activity/rest patterns are comparable between the three groups suggesting that the injection/manipulation does not have a marked sleep effect in female mice. Therefore, we do not expect “other factors” to have a significant contribution on the offspring’s phenotype.

4) Explained our interpretation about the long-term effects of the prenatal manipulation on the offspring’s phenotype. Please, find changes in the introduction (page 6, lines 113-115), discussion (page 30 lines 646-647; page 31 lines 648-649; page 33 lines 700-706) of the revised manuscript.

5) Regarding the translational relevance, the naïve group is the best “control” that can be used to compare the effects of the prenatal administration of GCs in humans because this treatment is given to 90% of pregnant women at risk of premature delivery. That is the main reason why we decided to perform treatments in- and out-of-phase.

**5)** Please elaborate on the design of exposure to CORT: justify why the exposure covered only GD15 and GD16? How was the exposure dose (5 mg/kg/day) selected?

**Response #44:**

We thank the reviewer for this comment and the possibility to explain the reasoning behind the experimental design.

1) The exposure was done only at GD15.5 and GD16.5 to improve as much as possible the translational value of our model in 2 aspects.

- In pregnant women antenatal GCs are indicated when there is a risk of premature delivery between gestational week 24 and 34, this period represents the 65-90% of a human pregnancy. The injections of pregnant dams at GD15 and 16 represent around 70-90% of a mouse pregnancy. Of course, we are aware that we work on experimental models and the extrapolation to human situation is not that simple.

- In the clinics, one of the most used courses are 2 injections with synthetic GCs 24 h apart from the moment the elevated risk of premature delivery is diagnosed.

2) The dose was selected to override the capacity of the placenta that in physiological conditions inactivates maternal CORT through the activity of 11β-hydroxysteroid dehydrogenase. In a previous paper from our laboratory (Astiz et al., Nat Comms 2020) we have shown that one hour after injection, the levels of CORT in the fetal blood increase to a concentration comparable to the maternal GC circadian peak. The dose of 5 mg/kg b.w we used here is comparable to a pharmacological dose in humans.

Please, find the changes in the method section (page 36 lines 759-764) of the revised manuscript.

**6)** 300 lux and assumed instantaneous transition between phases is a strong entrainer for the jetlag experiment. The same information is relevant for the experiments involving timed administration of CORT and sample collection. What was the light intensity in the holding facility? Where the transitions between light and dark abrupt, or gradual?

**Response #45:**

We thank the reviewer for this comment and the possibility to explain further our experimental design. Mice were always exposed to a light intensity of around 300 lux at the animal facility where we did the tissue collection for snRNA-seq (Fig 2 and 3), the timed administration of CORT (Fig 4 and 5), during the prenatal manipulation and the jetlag experiment in the offspring (Fig 1) and before preparing the SCN slices for the Ca^2+^ experiments (Fig 6). The transition from light to dark and dark to light was abrupt in all cases, unfortunately we do not have the possibility to model dawn and dusk. Therefore, we kept the same lighting conditions for all cohorts of mice included in this study. Please, find the information of this important environmental condition in the methods section (page 35 lines 745-748) of the revised manuscript.

**7)** Sex-related differences: the authors do not disclose the gender of the animals used in the jetlag experiment. The sex is particularly relevant in this context, provided sex-related differences in the outcome of prenatal exposure to GCs have been reported earlier. For jetlag experiments, see e.g. Elberling et al., Mol Neurobiol 2023.

**Response #46:**

We thank the reviewer for this comment. In the revised version of our manuscript, we have:

1) Added a new supplementary figure (Fig S1) including the actograms of all the mice included in Fig 1. Each running-wheel actograms shows the locomotor activity before and after jetlag and the sex of the mice. The distribution of male and female offspring is even for all the three groups. We assessed the phase-shift 50 (PS50) defined as the time at which half of the phase shift is completed in the three groups separated by sex (S1d Fig and Fig 11R below). Two-way ANOVA analysis with sex and prenatal treatment as variables showed a significant effect of the treatment (F2,39)=5.017, p=0.0115), no effect of sex (F1,39)=0.039, p=0.8427) and no interaction.

**Fig 11R:**

**Fig 11R: Sex comparison for the phase-shift 50 (PS50)** defined as the time at which half of the phase shift is completed. Data are expressed as mean ± SEM (naïve n=14, 8 males and 6 females, out-of-phase n=15, 7 males and 8 females and in-phase n=16, 8 males and 8 females). Data was 2-way ANOVA with sex and prenatal treatment as factors. The statistical analysis showed, no interaction of both variables (F(2,39)=0.0081, p=0.9919), no significant effect of sex (F(1,39) = 0.039, p=0.842) and a significant effect of the treatment (F2,39)=5.017, p=0.0115).

Please, find a comment about the new supplementary figure (S1 Fig) and a new sentence contextualizing our findings in the results (page 8, lines 150-152) of the revised manuscript.

**8)** Lines 787-790: Which clock genes have high expression levels in SCN neurons? Is this related to the time of collection (ZT)?

**Response #47:**

We thank the reviewer for this comment. While adult SCN express high levels of all clock genes in a circadian manner, the SCN during development express lower levels with a lower circadian amplitude. Compared with other hypothalamic nuclei the SCN is expressing higher overall levels of clock genes even during development and that is why we used them to check the dissection. We used a list of genes that are either members of the molecular clock machinery or well-known clock-controlled genes (*Per1/2/3, Cry1/2, Rorα/β/γ, Nr1d1/2, Arntl, Dpb, Npas2/3/4, Bhlhe40/41, Clock, Tsc22d3/4/5, Usp2*)*.* To avoid any kind of influence of the time of day in the expression of clock genes or clock-controlled genes (i.e.: from 20-40% of the cell transcriptome) we have collected all the samples for the transcriptome analysis always between ZT 3-5 (i.e.: *Zeitgeber time*, 3 to 5 hours after lights on). Please, find a comment in page 37 lines 779-781 of the revised manuscript.

**9)** Results: Did the authors note alterations in developmental milestones, such as decreased body weight? Did the authors observe correlations between early postnatal effects and PS50? One can see that the difference between out-of-phase exposure and controls id driven by 4 extreme values. Were these recorded in males or in females?

**Response #48:**

We thank the reviewers for this comment. We measured the body weight in the offspring of another cohort of the same experiment (in this case 4 males and 4 females). We found no difference in the weight due to the treatment, however, the females were in general lighter than the males (Fig 12R).

**Fig 12R:**

**Fig 12R: Body weight of male and female offspring** (8 weeks old). Data are expressed as mean ± SEM (naïve n=8, 4 males and 4 females, out-of-phase n=8, 4 males and 4 females and in-phase n=8, 4 males and 4 females). Data are expressed as mean ± SEM and analyzed by 2-way ANOVA with sex and prenatal treatment as factors. The statistical analysis showed, no interaction of both variables (F(2,18)=0.205, p=0.8166), significant effect of sex (F(1,18) = 25.42, p<0.0001) and no effect of the treatment (F2,18)=0.2083, p=0.8139).

Unfortunately, we did not measure early postnatal effects to correlate with PS50. However, we will consider this interesting aspect in future experiments. As observed in Fig 11R (above), the 4 values the reviewer is concerned about are coming from two males and two females, the variability observed is in our opinion intrinsic to the readout.

**10)** The illustration of activity recorded for the jetlag experiment contains several remarkable differences: (1) the amount of activity recorded in controls is obviously higher than in the other experimental groups; (2) circadian masking is obvious in controls, but totally absent in the other two groups; (3) unlike controls, both groups exposed to CORT display polyphasic circadian patterns of activity. This reviewer recommends commenting on the differences between circadian patterns, if they are consistent beyond the recordings used for illustration and across sexes (e.g., may illustrate hypoactivity induced by developmental exposure to CORT).

**Response #49:**

We thank the reviewer for this comment, this concern is shared by reviewer #1 (response #3). We agree that a weak entrainment to the LD cycle could impact the speed of re-entrainment after a shift in the LD cycle. In the revised version of our manuscript, we have:

1) Added a new supplementary figure (S1 Fig) including the actograms of all the mice included in Fig 1. Each running-wheel actograms shows the locomotor activity before and after jetlag (naïve n=14, out-of-phase n=15 and in-phase n=16), both sexes are included. On the right we show the 24-hour profile of locomotor activity in LD 12:12 (S1a Fig) and a zoom-in on the onset of activity in the LD transition (S1b Fig). In both cases, the running-wheel activity of 10 days prior to jetlag was averaged for each individual animal, the patterns of activity nicely overlap, no differences are observed between the groups. We also assessed the onset of activity the day before of the jetlag (S1c Fig), again no differences were found at the onset suggesting that the speed of re-entrainment after jet-lag does not depend on a weaker entrainment to the LD prior to the shift. Please, find the new supplementary figure (S1 Fig) and changes in the results (page 8 lines 150-152) of the revised manuscript.

2) Changed, in the main figure one, of the selected actograms (in-phase group) to provide a more representative example of the whole cohort.

**11)** Discussion: Line 565-567: "…evidence showing the nature of GCs influence on the circuit" Please rephrase. The use of "circuit" is not justified provided there is no recording of neuronal activity.

**Response #50:**

We thank the reviewer for this comment. We have rephrased this sentence.

**12)** Line 570-571: "GCs, one hormonal output pathway of the system" This sentence must be rephrased, because it is misleading. GCs are the output of HPA axis, which is regulated by AVP released from SCN in the PVN. The mechanisms downstream in the HPA axis are more complex, because the internal clock in adrenal glands regulate the response to ACTH, so that the sensitivity of adrenal cortex to ACTH has circadian fluctuations, and even constant ACTH stimulation will result in circadian rhythms in GC secretion.

**Response #51:**

We thank the reviewer for this comment. We have rephrased those sentences to improve clarity and scientific accuracy. Please, find changes in the introduction (page 4 lines 65-76) and the discussion (page 29, lines 623-624) of the revised manuscript.

**13)** Missing: there are 3 main processes with circadian oscillations in the SCN: clock gene expression; neuronal firing frequency; and Calcium waves. How do the results integrate in this context?

**Response #52:**

We thank the reviewer for this interesting comment that is shared with reviewer’s #1 and #2 and encouraged us to perform more experiments and explore further mechanistic details on the effects of CORT in the SCN.

For the revision of our manuscript we decided to perform **three new** experiments.

**1.** We performed the same sequence of Ca^2+^ events recordings in slices that were incubated before and during the recording in aCSF containing actinomycin D (a transcription inhibitor) and cycloheximide (a translation inhibitor). We then confirm that the increased Ca^2+^ events induced by CORT in SCN do not depend on transcriptional or post-transcriptional events.

Data are presented in Fig 6i and S10l

**2.** We performed the same sequence of Ca^2+^ events recordings in slices obtained from the mouse at ZT12. For these experiments we also incubated the slices before and during the recording in aCSF containing actinomycin D and cycloheximide. We then confirm that the effect of CORT is stronger (i.e.: higher number of Ca^2+^ events/minute) at ZT12 in alignment with previously published data showing that at that timepoint the SCN expects higher concentration of circulating GCs, when neuronal activity is low and astrocytic activity high. Interestingly, it also aligns with the re-analysis of previously published data suggesting that GR-related pathways are induced when physiological GC levels are at their peak (S9 Fig), suggesting that both fast and slow events are induced.

Data are presented in Fig 6j and S10m

**3.** We performed a treatment with CORT-BSA (corticosterone conjugated with bovine serum albumin) unable to cross the membrane. We show here that fast effects mediated by CORT are mediated by GR on the membrane, the effect is blocked by the GR antagonist RU486, again suggesting a different downstream pathway than the expected genomic effects.

Data are presented in Fig 6k and S10n

Although we are providing novel insights on the effect of CORT in the SCN, this is just the beginning, further experiments will be necessary to fully understand the complex interaction of gene expression, neuronal firing and Ca^2+^ waves. Please, find changes in the discussion (page 32 lines 681-691, page 33 lines 692-696 and 706-713 and page 34 lines 714-720)

**14)** The fact that SCN astrocytes react to GR activation explains the finding of Ono, Honma and Honma (SciRep 2015) that activity bouts drive bursts of clock gene expression in the SCN. This is particularly obvious for Per1, which also has hypersensitive GRE sequences in its promoter region (Reddy et al., MolCellBiol 2012), and can account for circadian entrainment by physical activity and meals.

**Response #53:**

We thank the reviewer very much for this comment. We completely agree, this is possible, and it may be an interesting idea to test the role of GCs in linking SCN gene expression and behavioral output. We have included Ono et al., 2015 paper and others in which we believe that the assessment of direct GC effects might be relevant.

**General comment about bioluminescence recordings:**

During the revision, we have re-analyzed the data from the bioluminescence assay as suggested by reviewer#1. When data are analyzed this way, we still see changes in the same direction and we still find them to be statistically significant (please, see **response #16-20**). The magnitude of the CORT effect, however, is markedly smaller with this analysis. An average phase delay of 2.12 ± 0.25 h (mean ± SEM) might not be biologically relevant.

Due to a bottleneck in mouse supplies, we were unable to perform further bioluminescence experiments to increase the sample size. Moreover, during the period of revision, a paper was published showing that dexamethasone (an agonist of the GR) does not have an effect shifting the phase of the SCN clock in adults (PMID: 41314247).

Given these considerations, and in light of the strong and compelling molecular biology (and now the Ca^2+^ imaging) results, we would like to follow editors’ and reviewers’ advice regarding this data set. We have removed figure 7 and associated supplementary material from the revised version because we feel the data is not providing relevant evidence. As reviewer #2 already commented, the manuscript would remain impactful even if bioluminescence data is deemphasized or removed.
