# Supplementary material for: A developmental shift in glucocorticoid receptor expression preserves glucocorticoid sensitivity in the adult suprachiasmatic nucleus
Source: PLoS Biol. 2026 Jul 7;24(7):e3003870. doi: 10.1371/journal.pbio.3003870 (PMC13340777; doi:10.1371/journal.pbio.3003870)
Supplement: Attachment — Submitted filename: Response to reviewers.docx [file pbio.3003870.s019.docx]

**PBIOLOGY-D-25-02226R2**

**Point-by-point response**

Reviewer’s comments (in black) are followed by the author’s responses (blue).

**Reviewer #1:**

Dear Dr. Smith, the revised manuscript by Handler et al. has addressed almost all of my previous comments, and with the new analyses and experiments is a much stronger package and I now find myself on balance agreeing with the majority of the claims put forth.  I thank the authors for their careful consideration and comprehensive response to my concerns.  The re-emphasis of the narrative and the addition of more calcium experiments has strengthened the relevance of the transcriptomic analyses.  I particularly like the inclusion of the Actinomycin D/cyclohexamide and CORT-BSA/RU data which confirms and extends the mechanism of the observation.

**Author’s response:** We would like to thank the constructive and thoughtful revision of our work. We agree with the reviewer that our paper has considerably improved.

**Minor Points**

**1)** It is a shame that the authors have not chosen to include data from the response to reviewers (Fig 1R, 2R and 7R) which make strong cases for some of the technical choices that they have made - I would encourage their inclusion in the supplementary data if possible.

**Author’s response:** We would like to thank the reviewer’s comment. We have included Fig 1R, 2R and 7R as supplementary material in the revised version of our manuscript. Please, find changes in the results (page 9, lines 193-195).

**2)** I appreciate that in the calcium data, the authors have weakened the statement that there is a wave of astrocytic activation in response to CORT - instead terming these synchronous events.  However, I take it that the lack of mention of statistical test in the figure legend for Fig. 6m means that there was no significance detected in the factorial test?  Either way, this should be reported so that the effect size can be assessed if the authors are asking the reader to believe that CORT treatment increases synchronous activity rather than just generically increasing activity.  At present, my interpretation is that the data is consistent with the latter - an observation supported by the Fig. 6g-h and 6m, especially as GAP26 treatment alone reduces activity. 

**Author’s response:** We appreciate the reviewer’s comment and apologize for the omission of the statistical test used in the legend of Figure 6m, which has now been corrected.

Briefly, we considered a factorial approach such as a two-way ANOVA (using treatment and event type as factors) but it was not appropriate for this dataset because test assumptions were not met. Instead, we used a Poisson GLMM with log(total events) as an offset and experiment as a random intercept, which directly models the *rate* of synchronized events per total events, while respecting both the count distribution and the paired experimental design. While a Poisson GLMM on raw counts confirmed a significant increase in synchronic events during CORT treatment (p = 0.037), the offset model revealed no significant effect of the treatment on the synchronization rate (Wald χ² = 1.00, df = 2, p = 0.608). Pairwise comparisons confirmed that no condition differed significantly from another (all Tukey-adjusted p > 0.5). Two complementary model specifications, one including total events as a covariate (p = 0.536) and one adding a Treatment × Total Events interaction (p = 0.357), yielded consistent results. Overdispersion was not a concern (Pearson dispersion ratio = 0.937).

We therefore agree with the reviewer that the observed increase in synchronized events cannot be dissociated from the overall increase in calcium event frequency. Please, find changes in the results (page 26 lines 546-55), methods (page 52 lines 1100-01, page 53 lines 1223-30) sections of the revised manuscript as well as in Fig 6 caption (page 29, lines 610-16). Synchronized events are now presented as an observation that parallels the global rise in activity under CORT treatment, rather than as evidence of a treatment-specific effect on astrocytic network synchronization.

**Reviewer #2:**

The authors have satisfactorily addressed all my concerns. I endorse publication of the manuscript in PLOS Biology.

**Author’s response:** We would like to thank the constructive and thoughtful revision of our work. We agree with the reviewer that our paper has considerably improved.

**Reviewer #4:** Stefan Spulber (note, reviewer 4 has signed this review):

The authors have addressed the points raised by this reviewer in a satisfactory manner. The inclusion of proper controls is a significant improvement and is relevant from the point of view of optimization of experimental design and use of animals. The contextualization of reported findings has also improved considerably.
I have no further comments and I endorse publishing the manuscript.

**Author’s response:** We would like to thank the constructive and thoughtful revision of our work. We agree with the reviewer that our paper has considerably improved.
