# Supplementary material for: A developmental shift in glucocorticoid receptor expression preserves glucocorticoid sensitivity in the adult suprachiasmatic nucleus
Source: PLoS Biol. 2026 Jul 7;24(7):e3003870. doi: 10.1371/journal.pbio.3003870 (PMC13340777; doi:10.1371/journal.pbio.3003870)
Supplement: Peer review history

# Editor report: Decision Letter 0

**DOI:** 10.1371/journal.pbio.3003870.r001

**Authors:** Lucas Smith

23 Jul 2025

Dear Dr Astiz,

Thank you for submitting your manuscript entitled "The central circadian clock and the developmental timeline of glucocorticoids sensitivity" for consideration as a Research Article by PLOS Biology.

Your manuscript has now been evaluated by the PLOS Biology editorial staff as well as by an academic editor with relevant expertise and I am writing to let you know that we would like to send your submission out for external peer review.

However, before we can send your manuscript to reviewers, we need you to complete your submission by providing the metadata that is required for full assessment. To this end, please login to Editorial Manager where you will find the paper in the 'Submissions Needing Revisions' folder on your homepage. Please click 'Revise Submission' from the Action Links and complete all additional questions in the submission questionnaire.

Once your full submission is complete, your paper will undergo a series of checks in preparation for peer review. After your manuscript has passed the checks it will be sent out for review. To provide the metadata for your submission, please Login to Editorial Manager (https://www.editorialmanager.com/pbiology) within two working days, i.e. by Jul 25 2025 11:59PM.

If your manuscript has been previously peer-reviewed at another journal, PLOS Biology is willing to work with those reviews in order to avoid re-starting the process. Submission of the previous reviews is entirely optional and our ability to use them effectively will depend on the willingness of the previous journal to confirm the content of the reports and share the reviewer identities. Please note that we reserve the right to invite additional reviewers if we consider that additional/independent reviewers are needed, although we aim to avoid this as far as possible. In our experience, working with previous reviews does save time.

If you would like us to consider previous reviewer reports, please edit your cover letter to let us know and include the name of the journal where the work was previously considered and the manuscript ID it was given. In addition, please upload a response to the reviews as a 'Prior Peer Review' file type, which should include the reports in full and a point-by-point reply detailing how you have or plan to address the reviewers' concerns.

During the process of completing your manuscript submission, you will be invited to opt-in to posting your pre-review manuscript as a bioRxiv preprint. Visit http://journals.plos.org/plosbiology/s/preprints for full details. If you consent to posting your current manuscript as a preprint, please upload a single Preprint PDF.

Feel free to email us at plosbiology@plos.org if you have any queries relating to your submission.

Kind regards,

Luke

Lucas Smith, Ph.D.

Senior Editor

PLOS Biology

lsmith@plos.org

# Peer review: Decision Letter 1

**DOI:** 10.1371/journal.pbio.3003870.r002

**Authors:** Lucas Smith

8 Sep 2025

Dear Dr Astiz,

Thank you for your patience while your manuscript " The central circadian clock and the developmental timeline of glucocorticoids sensitivity " was peer-reviewed at PLOS Biology. Your manuscript has been evaluated by the PLOS Biology editors, an Academic Editor with relevant expertise, and by several independent reviewers.

While the reviewers are quite positive about the study and its potential value to the community, they have raised a number of important concerns and suggestions to strengthen the manuscript further. Based on their specific comments and following discussion with the Academic Editor, it is clear that a substantial amount of work would be required to meet the criteria for publication in PLOS Biology. However, given our and the reviewer interest in your study, we would be open to inviting a comprehensive revision of the study that thoroughly addresses the reviewers' comments. Given the extent of revision that would be needed, we cannot make a decision about publication until we have seen the revised manuscript and your response to the reviewers' comments. Your revised manuscript would need to be seen by the reviewers again, but please note that we would not engage them unless their main concerns have been addressed.

Having discussed the reviews with the Academic Editor, we appreciate that the transcriptomic data are the strongest aspect of the study, and so we think the revision will largely need to focus on strengthening the physiological, mechanistic, and calcium imaging experiments.

While we think that some of the concerns should be relatively straightforward to address, other comments will require additional experiments and analyses. We think it will be essential to include the missing controls. For example, we agree with the reviewers that animal handling and injections could indeed induce corticosterone secretion, and naïve dams are not suitable controls. Similarly, changing the medium in astrocytic culture could trigger cellular responses, highlighting the need for vehicle controls in the Ca²⁺ experiments. The Ca²⁺ heatmap also appears problematic and will require more robust analysis and quantification. In addition, the bioluminescence assay requires substantial reinterpretation and reanalysis.

In addition to these revisions, you will need to complete some formatting changes, which you will receive in a follow up email. A member of our team will be in touch with a set of requests shortly.

We appreciate that addressing all the reviewer requests represents a great deal of extra work, and we are willing to relax our standard revision time to allow you 6 months to revise your study. Please email us (plosbiology@plos.org) if you have any questions or concerns, or envision needing a (short) extension.

At this stage, your manuscript remains formally under active consideration at our journal; please notify us by email if you do not intend to submit a revision so that we may withdraw it.

**IMPORTANT - SUBMITTING YOUR REVISION**

Your revisions should address the specific points made by each reviewer. Please submit the following files along with your revised manuscript:

1. A 'Response to Reviewers' file - this should detail your responses to the editorial requests, present a point-by-point response to all of the reviewers' comments, and indicate the changes made to the manuscript.

*NOTE: In your point by point response to the reviewers, please provide the full context of each review. Do not selectively quote paragraphs or sentences to reply to. The entire set of reviewer comments should be present in full and each specific point should be responded to individually, point by point.

You should also cite any additional relevant literature that has been published since the original submission and mention any additional citations in your response.

2. In addition to a clean copy of the manuscript, please also upload a 'track-changes' version of your manuscript that specifies the edits made. This should be uploaded as a "Revised Article with Changes Highlighted " file type.

*Resubmission Checklist*

When you are ready to resubmit your revised manuscript, please refer to this resubmission checklist: https://plos.io/Biology_Checklist

To submit a revised version of your manuscript, please go to https://www.editorialmanager.com/pbiology/ and log in as an Author. Click the link labelled 'Submissions Needing Revision' where you will find your submission record.

Please make sure to read the following important policies and guidelines while preparing your revision:

*Published Peer Review*

Please note while forming your response, if your article is accepted, you may have the opportunity to make the peer review history publicly available. The record will include editor decision letters (with reviews) and your responses to reviewer comments. If eligible, we will contact you to opt in or out. Please see here for more details:

https://blogs.plos.org/plos/2019/05/plos-journals-now-open-for-published-peer-review/

*PLOS Data Policy*

Please note that as a condition of publication PLOS' data policy (http://journals.plos.org/plosbiology/s/data-availability) requires that you make available all data used to draw the conclusions arrived at in your manuscript. If you have not already done so, you must include any data used in your manuscript either in appropriate repositories, within the body of the manuscript, or as supporting information (N.B. this includes any numerical values that were used to generate graphs, histograms etc.). For an example see here: http://www.plosbiology.org/article/info%3Adoi%2F10.1371%2Fjournal.pbio.1001908#s5

*Blot and Gel Data Policy*

We require the original, uncropped and minimally adjusted images supporting all blot and gel results reported in an article's figures or Supporting Information files. We will require these files before a manuscript can be accepted so please prepare them now, if you have not already uploaded them. Please carefully read our guidelines for how to prepare and upload this data: https://journals.plos.org/plosbiology/s/figures#loc-blot-and-gel-reporting-requirements

*Protocols deposition*

To enhance the reproducibility of your results, we recommend that if applicable you deposit your laboratory protocols in protocols.io, where a protocol can be assigned its own identifier (DOI) such that it can be cited independently in the future. Additionally, PLOS ONE offers an option for publishing peer-reviewed Lab Protocol articles, which describe protocols hosted on protocols.io. Read more information on sharing protocols at https://plos.org/protocols?utm_medium=editorial-email&utm_source=authorletters&utm_campaign=protocols

Thank you again for your submission to our journal. We hope that our editorial process has been constructive thus far, and we welcome your feedback at any time. Please don't hesitate to contact us if you have any questions or comments.

Sincerely,

Luke

Lucas Smith, Ph.D.

Senior Editor

PLOS Biology

lsmith@plos.org

-----------------------------------------

REVIEWS:

Reviewer #1: Dear Dr. Smith,

Please find attached my review for the manuscript "The central circadian clock and the developmental timeline of glucocorticoids sensitivity" by Händler et al, submitted for consideration in PLoS Biology.

In the manuscript, Händler et al, detail cell-type specific changes in the expression of glucocorticoid receptors (GR) in the suprachiasmatic nuclei (SCN) of the hypothalamus through development. The SCN, as the master mammalian coordinator of circadian rhythms, controls physiological and behavioural rhythms including the timing of glucocorticoid release from the which signals the beginning of the active phase. It is largely accepted that GR activation during development and in early life helps to align the SCN to the correct environmental time, however, this sensitivity is thought to drop off as mice reach maturity to prevent feedback onto the SCN disrupting circadian rhythms preferentially aligned by the light-dark cycle and other cues. In the current manuscript, the authors seek to address whether this is true and present new evidence that the SCN may in-fact retain glucocorticoid sensitivity into adulthood. Furthermore, they claim that this sensitivity is maintained by expression within the astrocytes of the SCN, and not the neurons. A major technical tour-de-force in this manuscript is the developmental single-nuclear transcriptomic dataset (snRNA-seq), which will be of interest to the neuroscience community generally, but of particular interest to researchers interested in development, circadian rhythms and hypothalamic circuits. However, the data that surrounds this dataset and the interpretation that the astrocytes alone mediate glucocorticoid responses in the adult SCN are less compelling. It is this Reviewer's opinion, that the manuscript would benefit from substantial refocussing and restructuring, additional controls that have been omitted and additional analysis of existing data. I would encourage the authors to reconsider their narrative and re-contextualise the dataset to present a balanced discussion of their findings.

Below, I have presented a detailed review:

Developmental exposure to GCs

The authors present an experiment where mice were given corticosterone either in-phase (in early morning) or out-of-phase (during the night) to stimulate circadian disruption and compare this to a control "naïve" condition where receive no manipulation. The correct control here would be mice that had been injected with saline s.c. at the in-phase or out-of-phase timepoints. This presents an important distinction, as all mice during pregnancy would have been handled and injected at the same gestational point. Handling and injection would induce a basal stress response in the animals, including hormones beyond corticosterone that may be relevant to the phenotype observed i.e. can the phenotype be pinned to incorrect timing of a corticosterone surge, or are other hormonal factors at play here and (as is alluded to by the authors) would result in sleep disruption at certain points which need to be controlled for as well. Data is presented later in the paper (Figure 4 b & e) confirms that vehicle treatment in this way does not result in the same magnitude corticosterone elevation as exogenous corticosterone treatment.

In Figure 1b the activity profiles for mice whose mothers received corticosterone injections seem to entrain more weakly to the LD cycle before the jet-lag protocol is applied, especially when compared to the naïve mouse. Is this consistent across the cohort? Analysis should be presented as to how well the onsets are locked to the LD cycle across these animals i.e. are the mice properly entrained to the LD cycle at the beginning of the experiment or is this a feature of CORT administration during the gestational period? Additional actograms should also be presented so that the precision of entrainment can be assessed. Given the relatively modest slowing in response to the jet-lag protocol, it is critical that the error of entrainment to the LD cycle is assessed. Could the modest slowing in the shift be that since the behaviour starts later in LD in these animals, it therefore takes slightly longer to catch up to the expected phase advance and represents a difference in entrainment rather than ability of the network to shift? A minor point regarding this data is that time is presented as solar (Daytime, hrs) when it should conventionally be presented as zeitgeber time (ZT) relative to the LD cycle.

In Figure 1c, the data presented shows an asterisk, presumably demarking significance between the curves. There is no mention in the text or legend as to the test performed here and the resulting significance. Likely, this would come from a two-way ANOVA with gestational experience and time post-phase shift as factors. The fit to the data is presented as a single line with error in the y-axis direction for the means. Details as to the error of the curves fitted should be presented as a dashed CI or similar.

In Figures 1e & f, a critical point here is that the authors present evidence that there is a reduction in GR/GFAP co-localisation in the out-of-phase offspring. They use this as the first evidence that the astrocytes are what are important in carrying this change in phase-shift dynamics. However, they do not mention whether there is a general decrease in GR staining overall across the SCN of these animals, or whether it is specific to the astrocytes. This is a critical point - is the modest phenotype that they measure here due to a reduction specifically in the astrocytes, or due to a general reduction in GR across the circuit as a whole?

Single Nuclear Transcriptomics

This dataset is the strongest part of this manuscript. The developmental snRNA-seq dataset will be a valuable tool for the wider community and the mapping back of the markers to the snRNAseq UMAP is a nice touch. For the staining, the insets chosen focus on the periphery of the SCN, rather than highlighting expression within the SCN itself - it would be nice to see insets from elsewhere in the SCN as well.

GR expression is limited to astrocytes

Beyond the general snRNAseq dataset itself, the authors focus on Nr3c1 (Gr) transcript expression which is mapped to their integrated UMAP plots in Figure 3a, revealing that as the SCN matures, Gr expression becomes more apparent in the astrocyte population. This is confirmed by the ridge plot analysis in Figure 3b that shows that the percentage of astrocytes expressing Gr transcript increases along the trajectory of SCN development. The authors allude to this representing a cell-type specific switch where the expression of GR goes up in the astrocytes and down in the neurons. However, it appears that the ridge plot only shows how much the percentage within that cellular population changes, but not the actual numbers of cells expressing GR within the SCN. Thus, even though it looks like there is a dramatic reduction in the proportion of Gr expressing cells in the dataset at PD30 (8.9% neurons expressing Gr versus 36.2% astrocytes expressing Gr), when you consider that there are around 3x the number of neurons compared to astrocytes these numbers become much less dramatic when considering the absolute differences. This is confirmed by the UMAP plots in Figure 3a, where the neurons appear to show a higher density of positive signal (albeit at a lower level of expression), which can also be seen in the pseudotime plots in Supplementary Figure 4.

The data in Figure 3c shows a very dramatic reduction in the %GR-expressing cells co-stained for VGAT. This is higher than I would expect from the ridge plots and UMAP where it appears that there should be equal or only slightly fewer absolute numbers of neurons expressing the Gr transcript at PND30. It therefore presents a discontinuity within this dataset. Has this analysis been done using the snRNAseq data i.e. co-expression analysis for % of cells with GR expression that also have these specific cell-type markers? Do these follow the same trend? This would bolster the argument that there is a cell-type specific switch.

Regardless of whether more astrocytes or neurons express GR in the adult SCN, the main conclusion drawn from the dataset in the text is: "At the protein level, we also found GR co-localising with astrocytes (GFAP+, Fig 3e) but not with neurons (VGAT+, Fig 3f)." This is in direct contradiction with the data presented in Fig. 3c, which shows that ~20% of GR is in neurons. Furthermore in the PLA experiment, as shown in Supplementary Figure 7, far more puncta are found in non-GFAP areas than are in GFAP areas. This is confirmed by Figures 5a & c, where only between 20-30% GR signal is co-localised to astrocytes (SOX9) - suggesting that other cells in the SCN express GR more widely than the astrocytes. From the snRNA-seq dataset, the best evidence is that it should be localised to the neurons as they are the only other cell-type that express Gr transcript, indeed this is confirmed by the GO term analysis (Supplementary Figure 4) where "…the GO biological term Cellular response to glucocorticoid stimulus were maintained at low levels in neurons".

The claim that astrocytes are the sole important party to mediate GC responses in the adult SCN is therefore not backed up by the evidence presented across various experiments. I see no reason that the authors need to discount the presence of extra-astrocytic expression of GR within the SCN. The focus solely on astrocytes detracts from two important conclusions that could be drawn from the manuscript: 1) Astrocytes in the SCN express GR (in addition to the neurons), and 2) the SCN remains responsive to GC signals in adulthood. It would be better if the authors refocused the manuscript to acknowledge and explore these points. This is of interest because the rest of the data shows SCN sensitivity to GC (as evidenced nicely in the PLA and GR-staining experiments in Figures 4 and 5 both in and outside astrocytes), suggesting that GR is functional within the SCN cells at this developmental timepoint, contrary to expectations from other published studies. The inclusion of the neurons here allows for a nice compare-and-contrast as an internal control to show that the same activation response is seen in both cell-types. This leads to further interesting questions: How might astrocytic- and neuronal-sensitivity to GCs differ or be similar? Is there cellular redundancy built in here, or is there a push-pull in the way astrocytes and neurons interact within the SCN network?

Calcium imaging in SCN slices

The calcium imaging is a nice addition to try and understand the response of the astrocytes to GC exposure. The authors have been very careful to try and exclude the influence of neuronal activity within their dataset, however, they may be missing important astrocytic responses that make interpretation of the data is difficult.

First, no vehicle control application was performed, so the response of astrocytes in the presence of an intervention has not been quantified i.e. does manipulation of the recording medium change astrocyte responses relative to baseline?

Second, the authors have set a very conservative threshold for detecting Ca2+ transients which limits most of these recordings to somatic Ca2+ activity, while missing Ca2+ activity in the astrocytic processes. While I understand that the authors wanted to be conservative in their approach, the responses in the astrocyte soma and processes have been shown to be complex in their integration. Ca2+ activity in the processes, close to the membrane, contributes substantially to waves of activity across the astrocytes as well as between cells and is mediated differently to Ca2+ activity in the soma (e.g. PMID26814587, PMID36937570, PMID40419752). As that is the focus of this experiment, I would encourage the authors to re-analyse this data with a view to better discriminating the response in the processes if possible. This may increase the number of quantifiable Ca2+ events across the SCN as currently they are very low (5-15 total events, across ~5 astrocytes in a single recording interval) and therefore make it difficult to draw conclusion.

Third, the heatmaps presented in Figure 6i to show Ca2+ wave propagation across the astrocytes are very weak evidence. To be convincing, surely the wave should be shown to propagate in the same manner (at least through the same interconnected cells, not necessarily in the same order dependent on where the wave initiates) multiple times i.e. is this a stochastic process or reproducible? To be more convinced of the initiation of intercellular calcium waves by GC treatment, some sort of correlation analysis should be performed. However, it may be that the number of events in the slices are too few to answer this on a limited sample size or that SCN astrocytes don't produce these calcium waves in response to GC treatment. Perhaps the authors could consider using a positive control treatment to reveal calcium waves in their preparation (e.g. mechanical stimulation, ATP or glutamate) so that the degree to which GC treatment induces this behaviour alongside a known initiator can be seen? Re-analysis of the data using a less conservative threshold (as above) may improve the ability to detect correlated waves of activity.

Finally, given that the previous datasets show that a small number of neurons express the GR, have the authors explored their neuronal dataset in this context (some cells responding, some not responding for example)? In other brain areas, calcium levels do not necessarily change immediately in neurons upon GC treatment (being delayed as transcriptional programmes control this) and their responses are complex (e.g. PMID23340218, PMID22153720, PMID39248182). Could the authors discuss potential differences between the neuronal response versus the astrocytic response?

Bioluminescence recordings

"Interestingly, the ascending phase of PER2::LUC expression typically occured around the end of the subjective light phase and the beginning of the subjective dark phase…". It is well established that the peak (end of the ascending phase) of PER2 expression marks the transition from the light phase to the dark phase (e.g. PMID10719897, PMID11181971), am I missing something with the definition of the ascending phase?

The authors describe the times of treatment as the "ascending phase" or the "descending phase", as evidenced above, this is ambiguous. Precise treatment phases should be calculatable from the PER2::LUC oscillation. It is well established in phase-response curves that there are "dead zones" where no shift occurs within both the ascending and descending intervals - are non-effective treatments hitting these phases? Treatments should be compared using the appropriate model via comparisons within restricted within the same treatment time-windows.

It is unclear how the phase-shift was calculated. In the methods, it is described as "the time when the fitted curve and the post-treatment curve crossed the zero-line of the first time after treatment" while in Figure 7a, it is indicated by an arrow pointing out the difference between the peaks of the PER2::LUC signal and the fitted curve. In fact, looking at the first point that the two plotted data cross the zero-line, it looks like there is no phase-shift in the GC treated slice. It is also unclear how the CORT was administered to the slice - is this a transient droplet treatment to the slice representing an acute spike or pulsatile GR activation, or addition to the medium representing a chronic GR activation? These two different administration methods represent two very different contexts for GR activation.

What are the error values associated with the fits used for the cosinor analysis? The example fit in Figure 7a seems poor and the waveform appears to change shape as the recordings continues - becoming more asymmetrical. It is hard to tell if this is a consequence of GC treatment or something to do with the slice health. Presenting other exemplar traces here would help to clear up this data set, as would the provision of additional traces for the other treatment and time conditions. The use of two-cycles in the baseline to fit the projected curve seems quite low and may not give a robust period estimate. Is there a period change post-treatment that may need to be factored into the analysis? If not, could the post-treatment interval be used to generate a more accurate curve that can be projected backwards from the treatment interval to the baseline interval?

The data in Figure 7c should be analysed as a two-way ANOVA where treatment, phase and the interaction between the two are tested.

Finally, although on the face of it the pharmacological data is compelling, the authors cannot discount the response of neurons to the GC stimulation in driving the phase change in these slices. The blockade of the response by GAP26 is evidence of astrocytic involvement, but does not prove that the GC stimulation is conducted via the astrocytes - what if blocking astrocytic communication via gap junctions prevents the neurons from generation a phase-shift regardless of the stimulus due to their influence on the network? Are comparable results seen with slices from younger mice where (according to the data) the response of astrocytes to GCs would be lesser? Are these responses still blocked by GAP26, representing the influence of astrocytes over neurons rather than their direct effects? The potential for astrocytic modulation of the neuronal response should be discussed and if possible explored.

Minor points:

The presentation of false-colored and merged images are difficult to assess. The images would be easier to digest if presented as split greyscale alongside merged images.

The in-situ hybridisation images are hard to assess, especially without the nuclear counterstain. This should be provided so that cellular colocalization can be better visualised.

What is the rationale for the choice of doses for the pharmacological agents administered?

Figure 1f is not referenced in the text.

Figure 3e, the inset region chosen to highlight astrocytic expression of GR sits outside the SCN and mostly represents extra-SCN signal.

In the figure legend for Figure 7c, CORT+GAP26 versus GAP26 gives a significant result on the Kruskal-Wallis test. This should be marked on the plot.

In Supplementary Figure 1, scale bars are missing from the images.

Supplementary Figure 5, the inset regions presented should be highlighted on the full-field images.

Supplementary Figure 8, the reanalysis of the publicly available datasets from Wen et al, 2020 and Morris et al, 2021 are nice, but I believe there is a misunderstanding regarding the Morris et al, 2021 dataset. Unless I am mistaken, this was performed on free-running SCN organotypic explants, not harvested from animals acutely. If this is the case, the interpretation of this data and detailing in the results (starting at line 398, page 19) and in the methods (starting at line 922, page 42) needs rewriting. If the analysis holds in the Morris et al, 2021 dataset, it means that even in the absence of circulating GCs the SCN still expects to receive the signal - it is a feature of the daily programme of the SCN.

Reviewer #2: This manuscript by Handler, Sreenivasan, Pilorez et al. addresses an important and longstanding question in circadian neuroscience: whether the adult SCN retains sensitivity to glucocorticoids. Prior evidence indicates that the glucocorticoid receptor (GR) is expressed prenatally in the SCN but absent in the adult SCN, though this issue has not been fully resolved. The authors show that prenatal exposure to glucocorticoids at the "wrong" circadian phase alters re-entrainment dynamics to jet lag later in adulthood. They report reduced GR expression in SCN astrocytes in these "out-of-phase" animals. Through snRNA-seq, they demonstrate a developmental shift in Gr expression from neurons to astrocytes. Using PLA and triple-label IHC, they further show that GR activation in astrocytes varies across circadian time and in response to CORT injection. Finally, they present physiological evidence: CORT-evoked astrocytic calcium activity (sensitive to gap-junction blockade) and phase-shifting of PER2::LUC rhythms. Overall, the manuscript presents compelling molecular evidence that SCN astrocytes remain glucocorticoid-responsive in adulthood, though I find the physiological experiments less convincing and in need of further rigor.

1) Astrocyte identification criteria: the calcium imaging experiments rely on complex morphological and temporal parameters to classify Ca²⁺ events as astrocytic. While the cited references provide partial justification, I did not find a clear precedent for the specific "astrocyte isolation profile" the authors employ. As noted by the authors themselves (Line 445), these parameters may under-detect certain events, and the choice of thresholds seems somewhat arbitrary.

2) Risk of neuronal contamination: the assertion (Line 447) that events longer than 10 s can be assumed to exclude neuronal activity is problematic. Calcium indicators are inherently slow, and Fluo-4 in particular is ill-suited to exclude the possibility that brief neuronal bursts could generate long-tailed transients. Thus, the >10 s threshold cannot guarantee clean separation between astrocytic and neuronal signals.

3) Statistical considerations: the number of recorded astrocytes is low, and it is not clear what constitutes the true statistical unit. A nested approach accounting for both cells and slices (animals) would strengthen the analysis. It would also be helpful to provide the exact number of contributing SCN slices for Fig. 6.

4) Calcium wave quantification: the claim of coordinated astrocytic calcium activation across ROIs (e.g., Fig. 6I) is not well supported by the heatmap. This should be quantified rather than described qualitatively.

5) Circadian timing: recordings were performed only at ZT 0-2. Given the well-established circadian modulation of both SCN physiology and glucocorticoid levels, repeating these experiments at additional circadian phases would provide much stronger support for the conclusions.

6) Event metrics: the chosen metrics (frequency, ΔF/F) are acceptable but incomplete. Considering the unusually long transients, integrated calcium (area under the curve) would be more meaningful, as it distinguishes brief events from prolonged ones.

7) Methodological alternatives: while the use of Fluo-4 with morphological/kinetic criteria is creative, a more definitive approach would be astrocyte-specific GECI expression (e.g., AAV-astrocyte promoter-Cre with Cre-dependent GCaMP). Even accounting for potential drawbacks of promoter selectivity and infection efficiency, such an approach would yield clearer and higher-throughput data than the current method.

8) PER2::LUC phase-shifting: the finding that CORT can delay PER2::LUC rhythms around subjective dusk is intriguing but requires more discussion in the context of prior reports that the SCN is largely insensitive to dexamethasone-induced phase shifts (Balsalobre et al., 2000; van der Veen et al., 2012; Ota et al., 2020). Reconciling these findings would strengthen the manuscript's impact.

My overall assessment is that the molecular biology results are strong and compelling, particularly the evidence for GR expression and activation in SCN astrocytes. By contrast, the physiological experiments (Ca²⁺ imaging and slice bioluminescence) are less robust and, in their current form, somewhat detract from the otherwise elegant story. The manuscript would remain impactful even if these physiological data were deemphasized or removed. If retained, the experiments require additional rigor and clarification as noted above.

Reviewer #3: General impression:

This is an interesting study addressing a topic of high translational potential. The manuscript presents an apparently solid set of data, but the way the experiments are introduced and have the results presented feels naïve and unsupervised.

The results are convincing, and the translational implications are touched upon, but the discussion ignores critical evidence from the literature.

Introduction:

Lines 65-70: The statement "One of the main hormonal outputs of the SCN are the "wakening" cues, glucocorticoids (GCs)…" is scientifically wrong. Please rephrase the sentence and split it into several clauses for clarity. Similarly, the last sentence of the paragraph is too vague and the wording is misleading: "Unlike other neuroendocrine systems, it is still unclear whether GCs target GR in the SCN and the influence on the circuit."

Reference 12 is cited wrong repeatedly bundled with references 10 and 11. While the latter do refer to effects of GCs on SCN during development, the experiments described in reference 12 refer to coupling between SCN and peripheral oscillators, albeit in mice exposed to GCs during early developmental stages.

The reference used for estimation of astrocyte:neuron ratio is rather old (1983). Are there more recent studies estimating the density of astrocytes in the SCN? How does this ratio tally with the authors' observations of astrocytes in the SCN?

Lines 112-113: please rephrase the following sentence for clarity: "Unlike in neurons, GR remains expressed in astrocytes also in the adult circuit."

Experimental model:

Control animals, denoted as "naïve", are not exposed to experimental procedures ("sham"), therefore the outcome can be the effect of combined manipulation (i.e. stress) and administration of CORT (as acknowledged in the Results, lines 142-143). Not having the controls exposed to sham procedures undermines the statement of translational relevance of the results (line 666).

Please elaborate on the design of exposure to CORT: justify why the exposure covered only GD15 and GD16? How was the exposure dose (5 mg/kg/day) selected?

300 lux and assumed instantaneous transition between phases is a strong entrainer for the jetlag experiment. The same information is relevant for the experiments involving timed administration of CORT and sample collection. What was the light intensity in the holding facility? Where the transitions between light and dark abrupt, or gradual?

Sex-related differences: the authors do not disclose the gender of the animals used in the jetlag experiment. The sex is particularly relevant in this context, provided sex-related differences in the outcome of prenatal exposure to GCs have been reported earlier. For jetlag experiments, see e.g. Elberling et al., Mol Neurobiol 2023.

Lines 787-790: Which clock genes have high expression levels in SCN neurons? Is this related to the time of collection (ZT)?

Results:

Did the authors note alterations in developmental milestones, such as decreased body weight? Did the authors observe correlations between early postnatal effects and PS50? One can see that the difference between out-of-phase exposure and controls id driven by 4 extreme values. Were these recorded in males or in females?

The illustration of activity recorded for the jetlag experiment contains several remarkable differences: (1) the amount of activity recorded in controls is obviously higher than in the other experimental groups; (2) circadian masking is obvious in controls, but totally absent in the other two groups; (3) unlike controls, both groups exposed to CORT display polyphasic circadian patterns of activity. This reviewer recommends commenting on the differences between circadian patterns, if they are consistent beyond the recordings used for illustration and across sexes (e.g., may illustrate hypoactivity induced by developmental exposure to CORT).

Discussion:

Line 565-567: "…evidence showing the nature of GCs influence on the circuit" Please rephrase. The use of "circuit" is not justified provided there is no recording of neuronal activity.

Line 570-571: "GCs, one hormonal output pathway of the system" This sentence must be rephrased, because it is misleading. GCs are the output of HPA axis, which is regulated by AVP released from SCN in the PVN. The mechanisms downstream in the HPA axis are more complex, because the internal clock in adrenal glands regulate the response to ACTH, so that the sensitivity of adrenal cortex to ACTH has circadian fluctuations, and even constant ACTH stimulation will result in circadian rhythms in GC secretion.

Missing: there are 3 main processes with circadian oscillations in the SCN: clock gene expression; neuronal firing frequency; and Calcium waves. How do the results integrate in this context?

The fact that SCN astrocytes react to GR activation explains the finding of Ono, Honma and Honma (SciRep 2015) that activity bouts drive bursts of clock gene expression in the SCN. This is particularly obvious for Per1, which also has hypersensitive GRE sequences in its promoter region (Reddy et al., MolCellBiol 2012), and can account for circadian entrainment by physical activity and meals.

# Author comment: Author response to Decision Letter 2

**DOI:** 10.1371/journal.pbio.3003870.r003

30 Mar 2026

Attachment Submitted filename: Point-by-point response .docx

# Peer review: Decision Letter 2

**DOI:** 10.1371/journal.pbio.3003870.r004

**Authors:** Lucas Smith

19 May 2026

Dear Dr Astiz,

Thank you for your patience while we considered your revised manuscript "The central circadian clock and the developmental timeline of glucocorticoid sensitivity" for publication as a Research Article at PLOS Biology. This revised version of your manuscript has been evaluated by the PLOS Biology editors, the Academic Editor and the original reviewers (note, that we enlisted the help of a new reviewer 4 to assess the response to reviewer 3 from the last round of review).

As you will see, below, all 3 reviewers are largely satisfied by the revision although reviewer 1 has a couple lingering requests that we think should be addressed. Based on the reviews assessment, we are likely to accept this manuscript for publication. However, before we can do so we need you to address the last reviewer comments, as well as a number of data and other policy-related requests, which I detail below.

**IMPORTANT - please address the following editorial requests:

1) TITLE: We would like to propose a tweak to your title, to try to include more of the specific findings of your paper. If you agree, we suggest you change your title to

"A developmental shift in glucocorticoid receptor expression from neurons to astrocytes preserves glucocorticoid sensitivity in the adult suprachiasmatic nucleus."

^we are happy to discuss this further.

2) TERMINOLOGY: We would like to suggest that you replace the word 'master' with 'central' (or something equivalent) when describing the SCN as a circadian regulator... The term 'master' has unfortunate historical connotations, and we think it would be better to use a less charged word, if possible. (if you are interested, we actually published a piece that touches on this, although in an ecology/evolutionary context... : https://journals.plos.org/plosbiology/article?id=10.1371/journal.pbio.3002933)

3) ETHICS STATEMENT: Thank you for providing an ethics statement in your methods section. Please update this to include the specific national or international regulations/guidelines to which your animal care and use protocol adhered. Please note that institutional or accreditation organization guidelines (such as AAALAC) do not meet this requirement.

4) DATA: Thank you for providing the underlying data for your paper as a deposition to GEO and a supplemental excel file. To be fully compliant with our policy (http://journals.plos.org/plosbiology/s/data-availability), we need a few more things here:

a. please remove the following from your data and code availability statement, in our online system: 'A previous version of this manuscript was deposited as a preprint in bioRxiv (10.1101/2024.03.04.583323). The copyright holder for this preprint is the author, who has granted bioRxiv a license to display the preprint in perpetuity. All rights reserved. No reuse allowed without permission.'

b. please update the data and code availability statement to say 'Raw transcriptomic data have been deposited in Gene Expression Omnibus under accession number GSE240803. Other underlying data can be found within the manuscript and supplemental files.'

c. please rename the raw data file as 'S1_data'

d. please update your raw data file to include the numerical data underlying the supplemental figures presented in your paper (if the data for those figures was not already included in your GEO deposition)

e. please add a sentence to each figure legend pointing readers to where the underlying data can be found.

5) CODE: Thank you for depositing your code on Github and including that in the 'data and code availability' statement. I see that you have provided a zenodo DOI for one set of scripts. Please also provide a Zenodo DOI for your https://github.com/SpielmannLab/SCN_development_glucocorticoid_haendler deposition. See the process for doing this here: https://docs.github.com/en/repositories/archiving-a-github-repository/referencing-and-citing-content

As you address these items, please take this last chance to review your reference list to ensure that it is complete and correct. If you have cited papers that have been retracted, please include the rationale for doing so in the manuscript text, or remove these references and replace them with relevant current references. Any changes to the reference list should be mentioned in the cover letter that accompanies your revised manuscript.

In addition to these revisions, you may need to complete some formatting changes, which you will receive in a follow up email. A member of our team will be in touch with a set of requests shortly. If you do not receive a separate email within a few days, please assume that checks have been completed, and no additional changes are required.

We expect to receive your revised manuscript within two weeks.

To submit your revision, please go to https://www.editorialmanager.com/pbiology/ and log in as an Author. Click the link labelled 'Submissions Needing Revision' to find your submission record. Your revised submission must include the following:

- a cover letter that should detail your responses to any editorial requests, if applicable, and whether changes have been made to the reference list

- a Response to Reviewers file that provides a detailed response to the reviewers' comments (if applicable, if not applicable please do not delete your existing 'Response to Reviewers' file.)

- a track-changes file indicating any changes that you have made to the manuscript.

NOTE: If Supporting Information files are included with your article, note that these are not copyedited and will be published as they are submitted. Please ensure that these files are legible and of high quality (at least 300 dpi) in an easily accessible file format. For this reason, please be aware that any references listed in an SI file will not be indexed. For more information, see our Supporting Information guidelines:

https://journals.plos.org/plosbiology/s/supporting-information

*Published Peer Review History*

Please note that you may have the opportunity to make the peer review history publicly available. The record will include editor decision letters (with reviews) and your responses to reviewer comments. If eligible, we will contact you to opt in or out. Please see here for more details:

https://plos.org/published-peer-review-history/

*Press*

Should you, your institution's press office or the journal office choose to press release your paper, please ensure you have opted out of Early Article Posting on the submission form. We ask that you notify us as soon as possible if you or your institution is planning to press release the article.

*Protocols deposition*

To enhance the reproducibility of your results, we recommend that if applicable you deposit your laboratory protocols in protocols.io, where a protocol can be assigned its own identifier (DOI) such that it can be cited independently in the future. Additionally, PLOS ONE offers an option for publishing peer-reviewed Lab Protocol articles, which describe protocols hosted on protocols.io. Read more information on sharing protocols at https://plos.org/protocols?utm_medium=editorial-email&utm_source=authorletters&utm_campaign=protocols

Please do not hesitate to contact me should you have any questions.

Sincerely,

Luke

Lucas Smith, Ph.D.

Senior Editor

lsmith@plos.org

PLOS Biology

------------------------------------------------------------------------

Reviewer remarks:

Reviewer #1: Dear Dr. Smith,

The revised manuscript by Handler et al. has addressed almost all of my previous comments, and with the new analyses and experiments is a much stronger package and I now find myself on balance agreeing with the majority of the claims put forth. I thank the authors for their careful consideration and comprehensive response to my concerns. The re-emphasis of the narrative and the addition of more calcium experiments has strengthened the relevance of the transcriptomic analyses. I particularly like the inclusion of the Actinomycin D/cyclohexamide and CORT-BSA/RU data which confirms and extends the mechanism of the observation.

Minor Points

It is a shame that the authors have not chosen to include data from the response to reviewers (Fig 1R, 2R and 7R) which make strong cases for some of the technical choices that they have made - I would encourage their inclusion in the supplementary data if possible.

I appreciate that in the calcium data, the authors have weakened the statement that there is a wave of astrocytic activation in response to CORT - instead terming these synchronous events. However, I take it that the lack of mention of statistical test in the figure legend for Fig. 6m means that there was no significance detected in the factorial test? Either way, this should be reported so that the effect size can be assessed if the authors are asking the reader to believe that CORT treatment increases synchronous activity rather than just generically increasing activity. At present, my interpretation is that the data is consistent with the latter - an observation supported by the Fig. 6g-h and 6m, especially as GAP26 treatment alone reduces activity.

Reviewer #2: The authors have satisfactorily addressed all my concerns. I endorse publication of the manuscript in PLOS Biology.

Reviewer #4, Stefan Spulber (note, reviewer 4 has signed this review): The authors have addressed the points raised by this reviewer in a satisfactory manner. The inclusion of proper controls is a significant improvement and is relevant from the point of view of optimization of experimental design and use of animals. The contextualization of reported findings has also improved considerably.

I have no further comments and I endorse publishing the manuscript.

# Author comment: Author response to Decision Letter 3

**DOI:** 10.1371/journal.pbio.3003870.r005

3 Jun 2026

Attachment Submitted filename: Response to reviewers.docx

# Editor report: Decision Letter 3

**DOI:** 10.1371/journal.pbio.3003870.r006

**Authors:** Lucas Smith

5 Jun 2026

Dear Dr Astiz,

Thank you for the submission of your revised Research Article "A developmental shift in glucocorticoid receptor expression preserves glucocorticoid sensitivity in the adult suprachiasmatic nucleus" for publication in PLOS Biology and thank you for addressing the last reviewer and editorial requests in this revision. On behalf of my colleagues and the Academic Editor, Sebastien G Bouret, I am pleased to say that we can in principle accept your manuscript for publication, provided you address any remaining formatting and reporting issues. These will be detailed in an email you should receive within 2-3 business days from our colleagues in the journal operations team; no action is required from you until then. Please note that we will not be able to formally accept your manuscript and schedule it for publication until you have completed any requested changes.

Please take a minute to log into Editorial Manager at http://www.editorialmanager.com/pbiology/, click the "Update My Information" link at the top of the page, and update your user information to ensure an efficient production process.

PRESS

We frequently collaborate with press offices. If your institution or institutions have a press office, please notify them about your upcoming paper at this point, to enable them to help maximise its impact. If the press office is planning to promote your findings, we would be grateful if they could coordinate with biologypress@plos.org. If you have previously opted in to the early version process, we ask that you notify us immediately of any press plans so that we may opt out on your behalf.

We also ask that you take this opportunity to read our Embargo Policy regarding the discussion, promotion and media coverage of work that is yet to be published by PLOS. As your manuscript is not yet published, it is bound by the conditions of our Embargo Policy. Please be aware that this policy is in place both to ensure that any press coverage of your article is fully substantiated and to provide a direct link between such coverage and the published work. For full details of our Embargo Policy, please visit http://www.plos.org/about/media-inquiries/embargo-policy/.

Thank you again for choosing PLOS Biology for publication and supporting Open Access publishing. We look forward to publishing your study.

Sincerely,

Luke

Lucas Smith, Ph.D.

Senior Editor

PLOS Biology

lsmith@plos.org
